# Adaptation of flea beetles to Brassicaceae: host plant associations and geographic distribution of *Psylliodes* Latreille and *Phyllotreta* Chevrolat (Coleoptera, Chrysomelidae)

**DOI:** 10.3897/zookeys.856.33724

**Published:** 2019-06-17

**Authors:** Matilda W. Gikonyo, Maurizio Biondi, Franziska Beran

**Affiliations:** 1 Research Group Sequestration and Detoxification in Insects, Max Planck Institute for Chemical Ecology, Hans-Knöll-Str. 8, 07745 Jena, Germany Max Planck Institute for Chemical Ecology Jena Germany; 2 Department of Health, Life and Environmental Sciences, University of L’Aquila, 67100 Coppito-L’Aquila, Italy University of L’Aquila Coppito-L’Aquila Italy

**Keywords:** Alticini, chemical plant defence, detoxification, glucosinolates, plant-insect interaction, secondary plant metabolites, sequestration

## Abstract

The cosmopolitan flea beetle genera *Phyllotreta* and *Psylliodes* (Galerucinae, Alticini) are mainly associated with host plants in the family Brassicaceae and include economically important pests of crucifer crops. In this review, the host plant associations and geographical distributions of known species in these genera are summarised from the literature, and their proposed phylogenetic relationships to other Alticini analysed from published molecular phylogenetic studies of Galerucinae. Almost all *Phyllotreta* species are specialised on Brassicaceae and related plant families in the order Brassicales, whereas *Psylliodes* species are associated with host plants in approximately 24 different plant families, and 50% are specialised to feed on Brassicaceae. The current knowledge on how *Phyllotreta* and *Psylliodes* are adapted to the characteristic chemical defence in Brassicaceae is reviewed. Based on our findings we postulate that *Phyllotreta* and *Psylliodes* colonised Brassicaceae independently from each other.

## Introduction

Plant-feeding insects are often classified as specialists or generalists according to their food plant range. While generalist insect herbivores are able to feed on plants that belong to distantly related plant families, specialist insect herbivores feed selectively on one or a few closely related plant species (Schoonhoven et al. 2005). Many phytophagous insects, including numerous leaf beetle species, have a narrow food plant range (Jolivet and Hawkeswood 1995; Forister et al. 2015), which is at least partially determined by toxic and deterrent plant secondary metabolites. Plants produce more than 200,000 different secondary metabolites, and many of them are involved in defence against herbivores (Mithöfer and Boland 2012). The distribution of secondary metabolites in related plant species often correlates with the food plant range of specialised insect herbivores, which evolved strategies to avoid, tolerate, or detoxify these defence compounds (Heckel 2014). Such adaptations presumably played an important role in the species diversification of plant-feeding insects (Ehrlich and Raven 1964; Futuyma and Agrawal 2009), but the specific molecular mechanisms underlying host plant adaptation, and their role in insect ecology and speciation, are largely unknown.

Several genera in the family Chrysomelidae include species that are specialised to feed on plants in the family Brassicaceae (Table 1). In the subfamily Chrysomelinae, the genera *Colaphellus*, *Entomoscelis*, and *Microtheca* feed primarily on Brassicaceae (Jolivet and Petitpierre 1976b; Nielsen 1988), whereas the genus *Phaedon* is associated with several different plant families, e.g. Asteraceae, Brassicaceae, Scrophulariaceae, and Ranunculaceae (Table 1). In the subfamily Galerucinae, the flea beetle genera *Phyllotreta*, *Psylliodes*, *Leptophysa*, *Caeporis*, and *Hemiglyptus* utilise Brassicaceae as host plants (Furth 1979; Nielsen 1988; Jolivet 1991; Nadein 2010). In addition, many other polyphagous chrysomelid genera feed occasionally on this plant family. However, within Chrysomelidae, the genera *Psylliodes* and *Phyllotreta* comprise the highest number of crucifer specialists.

**Table 1.** Overview of Chrysomelidae genera that are associated with Brassicaceae hosts plants.

**Genus**	**Approx. no. of species**	**Major host plant families**	**Known species feeding on Brassicaceae**	**References**
**Subfamily Chrysomelinae**				
*Chrysolina* Motschulsky, 1860	450	Lamiaceae	*C.cavigera*, *C.colasi*	Jolivet and Petitpierre 1976a, 1976b; Clark et al. 2004; Jurado-Rivera and Petitpierre 2015
*Colaphellus* Weise, 1916	15	Brassicaceae	*C.bowringi*, *C.hoeftii*, *C.sophiae*	Döberl 2010; Gavrilović et al. 2014; Bieńkowski and Orlova-Bienkowskaja 2015; Rheinheimer and Hassler 2018
*Entomoscelis* Chevrolat, 1836	14	Brassicaceae	*E.adonidis*, *E.americana*, *E.berytensis*, *E.nigriventris*, *E.orientalis*, *E.pilula*	Mohr 1966; Gerber 1994; Ge et al. 2009
*Microtheca* Dejean, 1835	15	Brassicaceae	*M.ochroloma*, *M.picea*, *M.punctigera*, *M.semilaevis*	Jolivet 1951; Balsbaugh 1978; Jolivet and Hawkeswood 1995; Ameen 1996; Clark et al. 2004; Menezes et al. 2005; Balusu et al. 2017
*Phaedon* Latreille, 1829	80	Brassicaceae, Ranunculaceae, Plantaginaceae, Asteraceae	*P.brassicae*, *P.cochleariae*, *P.laevigatus*, *P.prasinellus*, *P.viridis*	Ge et al. 2003, 2013, 2015; Clark et al. 2004; Lopatin 2005; Rheinheimer and Hassler 2018
*Timarcha* Latreille, 1829	316	Rubiaceae, Plantaginaceae	*T.intermedia*, *T.lugens*, *T.strangulata*	Jolivet and Petitpierre 1973; Gómez-Zurita et al. 2000a, 2000b; González-Megías and Gómez 2001
**Subfamily Galerucinae, Alticini**				
*Caeporis* Dejean, 1837	1	Brassicaceae	* C. stigmula *	Jolivet and Hawkeswood 1995; Cabrera and Rocca 2012; Nadein 2012
*Hemiglyptus* Horn, 1889	1	Brassicaceae, Hydrophyllaceae	* H. basalis *	Clark et al. 2004; Nadein 2012
*Leptophysa* Baly, 1877	15	Brassicaceae, Cleomaceae	*L.batesi*, *L.bordoni*, *L.littoralis*	Jolivet 1991; Jolivet and Hawkeswood 1995; Bechyné 1997; Flowers and Janzen 1997
*Phyllotreta* Chevrolat, 1836	242	Brassicaceae	see Suppl. material 3	This study; Heikertinger 1943; Furth 1979; Smith 1985; Clark et al. 2004
*Psylliodes* Latreille, 1829	207	Brassicaceae, Poaceae	see Suppl. material 1	This study; Furth 1983; Cox 1998; Clark et al. 2004; Nadein 2010; Baviera and Biondi 2015

Glucosinolates are the characteristic secondary metabolites of Brassicaceae and other families in the order Brassicales (Agerbirk and Olsen 2012). Upon herbivory, glucosinolates are hydrolysed by β-thioglucosidase enzymes (myrosinases) to unstable aglucones, which can generate various hydrolysis products such as isothiocyanates, thiocyanates, and nitriles (Wittstock et al. 2016). Isothiocyanates, the most toxic glucosinolate hydrolysis products, are primarily reactive towards thiol- (-SH) and amino- (-NH_2_) groups in peptides and proteins (Brown and Hampton 2011). Previous studies revealed that insects developed different strategies to overcome this plant defence (reviewed in Winde and Wittstock (2011) and Jeschke et al. (2016)). For example, *Plutellaxylostella* larvae (Lepidoptera, Plutellidae) prevent glucosinolate breakdown by rapidly converting ingested glucosinolates to stable desulfo-glucosinolates (Ratzka et al. 2002), while *Pierisrapae* larvae (Lepidoptera, Pieridae) express a nitrile specifier protein (NSP) in their gut, which promotes the formation of less toxic nitriles instead of isothiocyanates (Wittstock et al. 2004). The evolution of NSP activity in Pierinae butterflies is regarded as an evolutionary key innovation that enabled a host shift from Fabalesplants to the glucosinolate-containing Brassicales. As predicted by the coevolutionary 'escape and radiate' hypothesis, speciation rates were higher in the clade that colonised Brassicales plants compared to their sister taxon (Wheat et al. 2007; Edger et al. 2015). In contrast, the host shift of Ceutorhynchini weevils from the plant family Lamiaceae to Brassicaeae was not associated with a speciation rate shift (Letsch et al. 2018).

Glucosinolates and their hydrolysis products are well known to affect the behavior of crucifer-feeding Chrysomelidae (reviewed in Mitchell (1988, 1994), and Nielsen (1988)). Volatile isothiocyanates, for example, attracted high numbers of *Phyllotreta* spp. and *Psylliodeschrysocephala* in field trapping experiments, indicating that isothiocyanates might play a role in host plant localisation (Görnitz 1956; Bartlet et al. 1992; Pivnick et al. 1992; Tóth et al. 2007). Glucosinolates, on the other hand, stimulated feeding of *Phyllotreta* spp., *Ps.chrysocephala*, *Phaedoncochleariae*, and *Entomoscelisamericana* in laboratory experiments (Hicks 1974; Mitchell 1978; Nielsen 1978; Bartlet et al. 1994; Reifenrath and Müller 2008). Although these specialists are adapted to the glucosinolate-myrosinase defence system, both glucosinolate levels and myrosinase activity affected herbivory by *Phyllotretacruciferae* in the field. The highest flea beetle damage was observed on *Brassicarapa* plants with intermediate glucosinolate levels (Siemens and Mitchell-Olds 1996), and *B.rapa* lines selected for high myrosinase activity displayed significantly less feeding damage (ca. 10%) than those with low enzyme activity (Mitchell-Olds et al. 1996). In contrast, studies with *Ps.chrysocephala* did not reveal a correlation between glucosinolate levels and feeding damage (Bartlet et al. 1996; Bartlet et al. 1999).

Here, we provide an overview on the host plants, diet breadth, and geographic distribution of known *Phyllotreta* and *Psylliodes* species, as well as their proposed relationships to other genera of Alticini. Diet breadth was classified according to Biondi (1996). Species feeding on one or two closely related botanical genera are considered as monophagous, species feeding on more plant genera of one or two closely related families are defined as oligophagous, and species feeding on many distantly related plant species are considered as polyphagous. For species with limited information on food plants, we did not specify the diet breadth. Data on the geographical distribution of the Palearctic *Psylliodes* and *Phyllotreta* species was primarily obtained from Döberl (2010) and is described according to Löbl and Smetana (2010). The zoogeographical regions are abbreviated as follows: Afrotropical Region (AFR), Australian Region (AUR), Nearctic Region (NAR), Neotropical Region (NTR), Oriental Region (ORR), Palearctic Region (PAR). In the second part of this review, we summarise the knowledge on the adaptations of *Phyllotreta* and *Psylliodes* spp. to the glucosinolate-myrosinase defence system and other defences in their host plants.

## Host plant associations of *Psylliodes* and *Phyllotreta* flea beetles

The genus *Psylliodes* Latreille, 1829 comprises over 200 species (Suppl. material 1). Adult *Psylliodes* beetles are distinguished from other flea beetle genera based on their 10-segmented antennae and tarsi inserted pre-apically on the metatibia of the hind legs. Most other Alticini genera have 11-segmented antennae except for *Psylliodes*, *Decaria*, and *Monotalla* with ten segments and *Nonarthra* with nine segments (Konstantinov and Vandenberg 1996; Nadein and Bezděk 2014). The genus comprises five subgenera: *Psylliodes* s. str. (194 species), *Semicnema* Weise (5 species), *Eupus* Wollaston (5 species), *Minicnema* Nadein (2 species) and *Psyllobactra* Lopatin (1 species) (Nadein 2007a, 2010). A subdivision of the subgenusPsylliodes s. str. based on morphological features was proposed by Leonardi (1970) and Nadein (2006, 2007a, 2007b) (Suppl. material 2).

According to the literature, host plants of 107 *Psylliodes* species have been reported, and these belong to 24 plant families (Suppl. material 1). Most *Psylliodes* species have a restricted host plant range (35% are monophagous and 51% are oligophagous), and only 14% are polyphagous. For instance, *Psylliodestoelgi* feeds only on *Biscutellalaevigata* (Brassicaceae), whereas *Psylliodesluteola* has been recorded on Poaceae, Fagaceae, Salicaceae, Ulmaceae, and Solanaceae.

**Figure 1.**
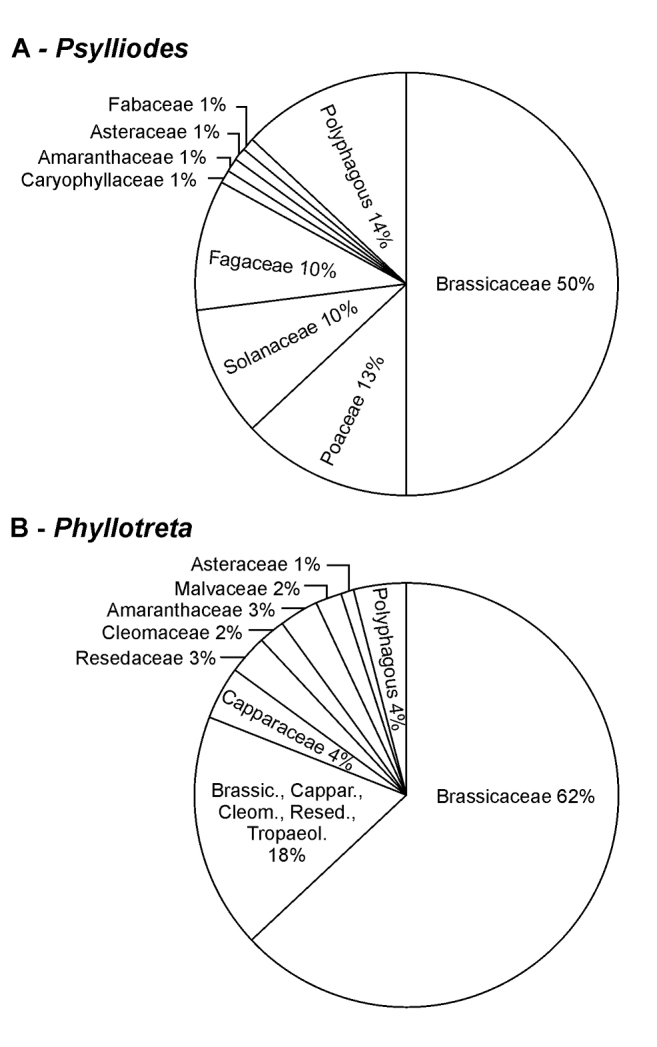
Host plant associations of the genera *Psylliodes* (**A**) and *Phyllotreta* (**B**). The host plants of 107 *Psylliodes* species and 117 *Phyllotreta* species have been reported in the literature. The numbers of species which feed on plants in one plant family (monophagous and oligophagous), and the number of polyphagous species are given as percentages. 18% of the *Phyllotreta* species feed on more than one family in the order Brassicales (Brassic., Brassicaceae; Cappar., Capparaceae; Cleom., Cleomaceae; Resed., Resedaceae; Tropaeol., Tropaeolaceae). For detailed information, refer to Suppl. material 1 (*Psylliodes*) and 3 (*Phyllotreta*).

Of all *Psylliodes* species with known host plants, 50% are specialised on Brassicaceae, followed by 13% feeding on Poaceae, 10% on Solanaceae and 10% on Fagaceae (Fig. 1A). Previous surveys of host plant associations of *Psylliodes* spp. focused on specific countries or regions and thus included a much smaller total number of *Psylliodes* species (Furth 1983; Cox 1998; Döberl 2010; Baviera and Biondi 2015). Interestingly, host plant use often correlates with the proposed *Psylliodes* s. str. species groups, which indicates that presumably closely related *Psylliodes* species feed on closely related host plants (Suppl. material 2). For example, *Psylliodes* species in the *chrysocephala* and *pyritosa* groups are specialised to feed on Brassicaceae, while species in the *luteola* group are mainly associated with Fagaceae.

The genus *Phyllotreta* Chevrolat, 1836 comprises about 242 species and host plant information is available for 117 species (Suppl. material 3). Most *Phyllotreta* species are specialised on glucosinolate-containing plants in the order Brassicales (Fig. 1B). An analysis of the diet breadth of *Phyllotreta* species revealed that 31% are monophagous, 64% are oligophagous, and 5% are polyphagous. In *Phyllotreta*, 63% are specialised on Brassicaceae, whereas 18% feed on plants in more than one family in the order Brassicales (Fig. 1B).Very few *Phyllotreta* species feed on plant families, which do not contain glucosinolates, for instance, *Phyllotretacruralis* is specialised on Amaranthaceae.

Several *Psylliodes* and *Phyllotreta* species are of economic importance. The cabbage stem flea beetle, *Ps.chrysocephala* is a serious pest of winter oilseed rape in Northern Europe (Zimmer et al. 2014), whereas *Phyllotretastriolata* and *Ph.cruciferae* are oilseed rape pests in Canada where their damage causes losses of tens of millions of US dollars annually (Lamb 1989; Hill 2008; Knodel 2017). On the other hand, the Palearctic species *Psylliodeschalcomera* (feeding on Asteraceae) was introduced to North America in 1997 as a control agent for the invasive weed *Carduusnutans* (musk thistle), but it likely did not establish in the Nearctic region (Antonini et al. 2008).

## Geographic distribution of *Psylliodes* and *Phyllotreta* flea beetles

**Figure 2.**
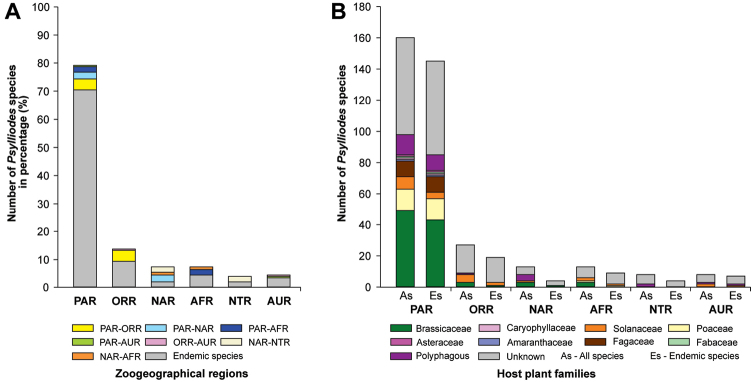
Distribution of 207 *Psylliodes* species in the different zoogeographical regions (**A**), and host plant associations of all species (As) and endemic species (Es) for each zoogeographical region (**B**). For detailed information, refer to Suppl. material 1.

The genus *Psylliodes* has a worldwide distribution (Biondi and D’Alessandro 2018). The highest number of species occurs in the Palearctic region (160 species, 145 endemic species), followed by the Oriental region (27 species, 19 endemic species), the Nearctic region (13 species, 4 endemic species), the Afrotropical region (13 species, 9 endemic species), the Neotropical region (8 species, 4 endemic species), and the Australian region (8 species, 7 endemic species; Suppl. material 1). A graphical overview of the species distribution is shown in Figure 2A; the host plant associations of all species and endemic species in each zoogeographical region are shown in Figure 2B. Some species are wide-spread in more than one zoogeographical region such as *Ps.brettinghami* (feeding on Solanaceae), which is found in the Australian, Oriental, and Palearctic regions, while others are strictly endemic to very limited areas, e.g. *Ps.tarsata*, which is only found on Madeira (Portugal). *Psylliodes* species that are endemic to the Palearctic region account for 83% of those associated with Brassicaceae. All other Brassicaceae-feeding species are found in other zoogeographical regions except for Australia (Fig. 2B).

**Figure 3.**
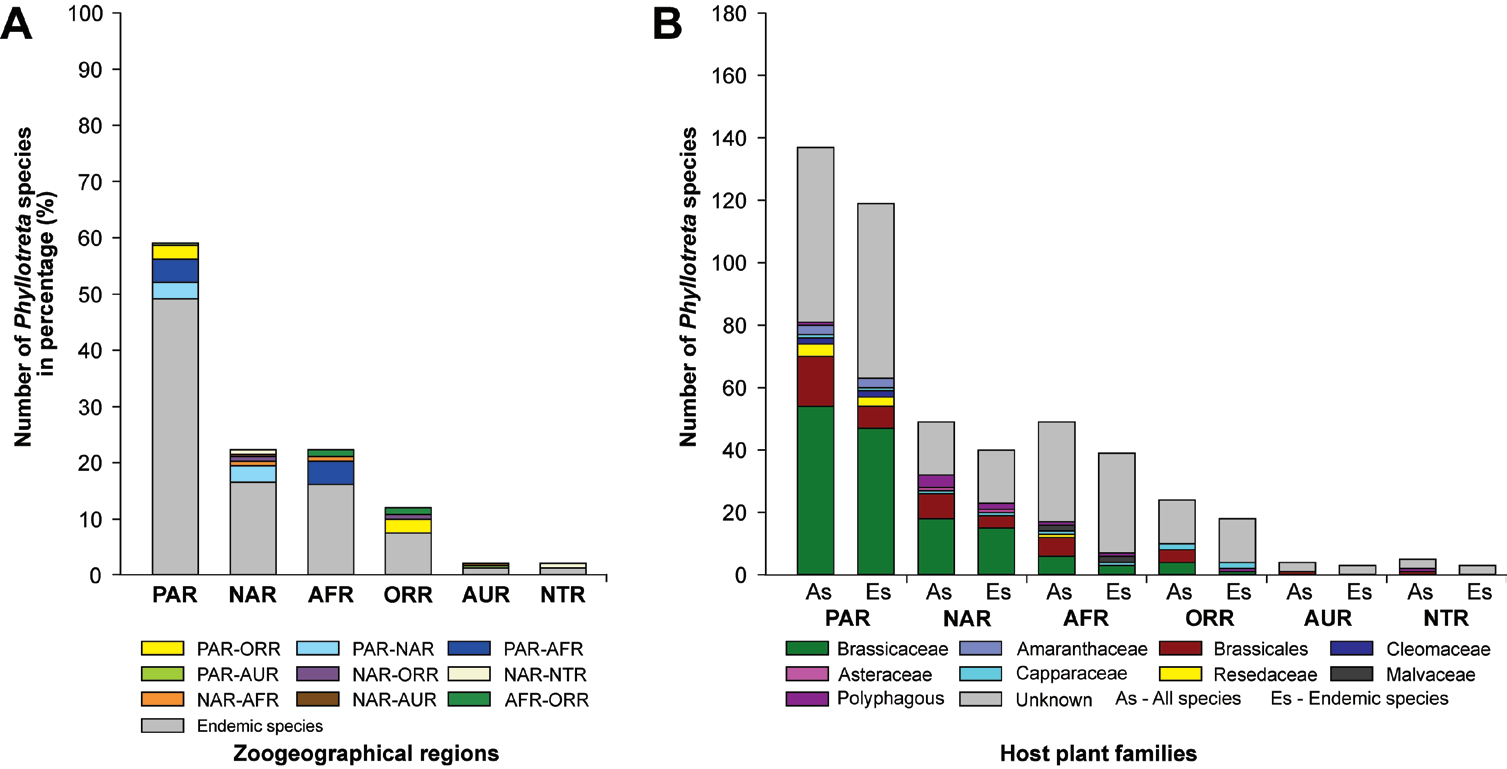
Distribution of 242 *Phyllotreta* species in the different zoogeographical regions (**A**), and host plant associations of all species (As) and endemic species (Es) for each zoogeographical region (**B**). For detailed information, refer to Suppl. material 3.

The geographic distribution of the genus *Phyllotreta* shows the highest number of species in the Palearctic region (137 species, 118 endemic species) followed by the Afrotropical region (49 species, 39 endemic species), the Nearctic region (49 species, 40 endemic species), the Oriental region (25 species, 18 endemic species), the Neotropical Region (5 species, 3 endemic species), and the Australian Region (4 species, 3 endemic species; Suppl. material 3). The species distribution is shown in Figure 3A, and the host plant associations of all species and endemic species in each zoogeographical region are shown in Figure 3B. In general, a high percentage of endemic *Phyllotreta* species is found in all geographical regions (≥ 60%) with highest values in the Palearctic, Afrotropical, and Nearctic regions (≥ 80%). In some areas, especially in the Nearctic region, several species of *Phyllotreta* are not native and have been introduced from other regions (Milliron 1953; Smith 1985). Most species feeding on Brassicaceae are found in the Palearctic and Nearctic regions. The host plants of a large proportion of the species endemic to the Afrotropical, Australian, and Neotropical regions are unknown (Fig. 3B; Suppl. material 3).

## Phylogenetic relationships of *Psylliodes* and *Phyllotreta* to other Alticini

The most comprehensive phylogenetic analyses of the subfamily Galerucinae*sensu lato* are those of Ge et al. (2011, 2012) and Nie et al. (2018), which included about 80 and 70 genera of Alticini (including problematic genera), respectively. Ge et al. (2011, 2012) used two mitochondrial (16S rRNA and cytochrome oxidase (cox) 1) and two nuclear genes (18S and 28S rRNA) to infer phylogenetic relationships, while Nie et al. (2018) used the mitochondrial genome and nuclear rRNA genes. In these analyses, *Psylliodes* and *Phyllotreta* were never retrieved as sister genera, but instead clustered in distinct clades with other Alticini as summarised in Table 2. All three studies suggest a close phylogenetic relationship of *Psylliodes* to *Chaetocnema* and *Crepidodera* (see Table 2 for Bayesian posterior probability values and/or Maximum Likelihood bootstrap support values). Surprisingly, two different *Crepidodera* species included in the analysis of Nie et al. (2018) were not monophyletic. *Crepidoderapluta* clustered in the *Chaetocnema* group with *Psylliodes*, while the second *Crepidodera* sp. clustered together with two *Phyllotreta* species in a distant clade. However, the proposed relationships of *Phyllotreta* to other Alticini differ among the studies, and are usually less supported than those suggested for *Psylliodes*. None of the genera with proposed close phylogenetic relationships to *Phyllotreta* and *Psylliodes* are associated with Brassicaceae plants (Table 2).

**Table 2.** Phylogenetic relationships of *Psylliodes* and *Phyllotreta* to other Alticini genera.

Study	* Psylliodes *	* Phyllotreta *
Ge et al. (2011)	Sister genus: *Chaetocnema* (Poaceae)**^1^**	Sister genus: *Batophila* (Rosaceae)
	Phylogenetic support (B/ML): 0.84/67	Phylogenetic support (B/ML): 0.79/<50
	Clade: *Crepidodera* (Salicaceae),	Clade: *Lipromela* (unknown),
	*Epitrix* (Solanaceae)	*Syphrea* (Euphorbiaceae),
	Phylogenetic support (B/ML): 0.52/<50	*Altica* (Onagraceae, Lythraceae),
	Taxonomic group: Unspecified	*Macrohaltica* (Gunneraceae)
		Phylogenetic support (B/ML): 0.98/<50
Ge et al. (2012)	**Bayesian and Maximum-Likelihood phylogenies**	**Bayesian phylogeny**
	Sister genus: *Chaetocnema* (Poaceae)	Sister genus: *Epitrix* (Solanaceae)
	Phylogenetic support (B/ML): 0.95/67	Phylogenetic support (B): 0.95
	Clade: *Crepidodera* (Salicaceae),	Clade: *Diphaltica* (Aquifoliaceae),
	*Epitrix* (Solanaceae), *Syphrea* (Euphorbiaceae), *Altica* (Onagraceae, Lythraceae),	*Agasicles* (Amaranthaceae), *Disonycha* (Amaranthaceae)
	*Macrohaltica* (Gunneraceae)	Phylogenetic support (B): 0.81
	Phylogenetic support (B/ML): 0.89/<50	**Maximum-Likelihood phylogeny**
	Taxonomic group: *Chaetocnema*	Clade: *Lanka* (Piperaceae),
		*Longitarsus* (Boraginaceae),
		*Tegyrius* (Piperaceae)
		Phylogenetic support (ML): <50
Nie et al. (2018)	Sister genus: *Chaetocnema* (Poaceae),	Sister genus and clade:
	*Epitrix* (Solanaceae)	*Crepidodera* (Salicaceae)
	Phylogenetic support (B): 0.48	Phylogenetic support (B): 0.83
	Clade: *Crepidodera* (Salicaceae),	
	*Xuthea* (Urticaceae)	
	Phylogenetic support (B): 0.89	
	Taxonomic group: *Chaetocnema*	

^1^The major host-plant family for each genus according to Jolivet and Hawkeswood (1995) is given in parentheses. ML – Maximum Likelihood bootstrap value; B – Bayesian posterior probability

## Adaptations of crucifer-feeding flea beetles to chemical plant defences

An unexpected observation revealed that *Ph.striolata* adults emit low amounts of toxic isothiocyanates, which are derived from glucosinolates that are stored at high concentrations of up to 50 µmol/g fresh weight (ca. 2% of the body weight) in adults (Beran 2011; Beran et al. 2014). When adults were transferred to different crucifer species, they selectively accumulated mainly aliphatic glucosinolates from their food plants, e.g. allyl glucosinolate from *Brassicajuncea*, and 4-methylsulfinylbutyl (4MSOB) glucosinolate from *Arabidopsisthaliana*. In contrast, adults sequestered only low amounts of the benzenic 4-hydroxybenzyl glucosinolate from *Sinapisalba*. The glucosinolate accumulation pattern depended both on glucosinolate structure and on the host plant background, suggesting that the plants’ glucosinolate composition affects sequestration in *Ph.striolata*. The ability to accumulate high glucosinolate amounts demonstrates that *Ph.striolata* can at least partially prevent activation of ingested glucosinolates. However, quantitative feeding studies, for instance with radiolabeled glucosinolates, are needed to determine to which degree ingested glucosinolates are sequestered intact.

**Figure 4.**
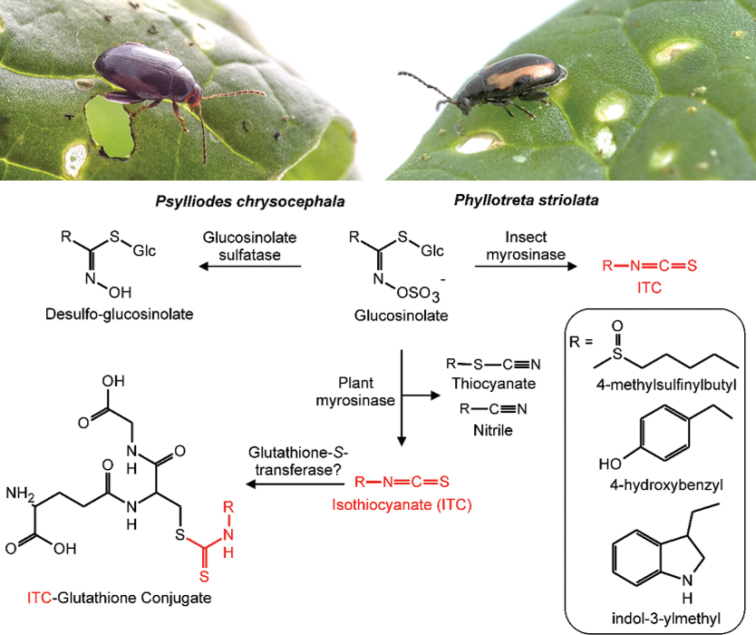
Metabolism of glucosinolates in *Psylliodeschrysocephala* and *Phyllotretastriolata*. Upon herbivory, glucosinolates are usually hydrolysed by the plant enzyme myrosinase to an unstable aglucone, which spontaneously rearranges to a toxic isothiocyanate. In the presence of plant specifier proteins, other hydrolysis products such as thiocyanates and nitriles are formed. Both flea beetle species sequester glucosinolates in their bodies, suggesting that not all glucosinolates are hydrolysed in feeding-damaged plant tissue. Sequestered glucosinolates may be activated for defensive purposes by an insect myrosinase in *Ph.striolata*, but not in *Ps.chrysocephala*. In addition, *Ps.chrysocephala* partially detoxifies glucosinolates by desulfation, whereas no glucosinolate sulfatase activity was found in *Ph.striolata*. According to a quantitative feeding study performed with *Ps.chrysocephala*, most ingested glucosinolates are activated, and isothiocyanates are detoxified by conjugation to glutathione. The isothiocyanate-glutathione conjugate is metabolized via the mercapturic acid pathway to several cyclic metabolites in *Ps.chrysocephala* adults (Beran et al. 2018). Examples of three structurally different glucosinolate side-chains are shown in the box. Beetle photos: Anna Schroll.

To activate sequestered glucosinolates, *Ph.striolata* possesses an insect myrosinase with high activity towards aliphatic glucosinolates, which evolved from insect β-*O*-glucosidases (Figure 4; Beran et al. 2014). To investigate how *Ph.striolata* activate sequestered glucosinolates and prevent autointoxication, dissected tissues from adults were analysed for the presence of glucosinolates and myrosinase activity, respectively. Interestingly, both glucosinolates and myrosinase were mainly localised in the hemolymph and elytra (Beran and Ahn, unpublished), but whether both components are stored separately in hemoplasma and hemocytes as previously reported for cyanogenic glycosides and the cyanogenic β-glucosidase in *Zygaenafilipendulae* larvae (Lepidoptera, Zygaenidae; Pentzold et al. 2017), is not yet known.

In the genus *Psylliodes*, the cabbage stem flea beetle, *Ps.chrysocephala*, selectively sequesters glucosinolates as well, but compared to *Ph.striolata*, glucosinolate concentrations are much lower (ca. 4 µmol/g fresh weight; Beran et al. 2018). Although glucosinolates are present in all life stages of *Ps.chrysocephala*, a defensive function is unlikely, as neither larvae nor adults possess endogenous myrosinase activity (Beran et al. 2018). An analysis of the metabolic fate of ingested 4MSOB glucosinolate in *Ps.chrysocephala* adults revealed that adults utilise at least three strategies to prevent isothiocyanate formation and toxicity. *Ps.chrysocephala* sequester intact glucosinolates, detoxify glucosinolates by desulfation, and detoxify dietary isothiocyanates by conjugation to glutathione. The isothiocyanate-glutathione conjugate is metabolised via the conserved mercapturic acid pathway to three different cyclic cysteine conjugates, which are excreted. These three strategies accounted for the metabolic fate of 18.5%, 8%, and 17% of the total ingested glucosinolates, respectively. The amounts of other glucosinolate breakdown products (4MSOB-isothiocyanate, -cyanide, -amine, and –acetamide) corresponded to 17.5% of the total ingested glucosinolate (Figure 4; for details, refer to Beran et al. 2018). However, the metabolic fate of about 39% of the total ingested glucosinolate remained unknown in this study.

The detoxification of isothiocyanates in *Ps.chrysocephala* comes at the expense of the amino acid cysteine. Therefore, interference with protein digestion, for instance by plant proteinase inhibitors or other digestibility reducers, might affect the detoxification capacity for isothiocyanates by limiting the availability of cysteine for glutathione biosynthesis. Interestingly, there is evidence that *Ps.chrysocephala* can compensate for the ingestion of plant proteinase inhibitors. *Ps.chrysocephala* larvae reared on a transgenic *Brassicanapus* line that overexpressed the cysteine proteinase inhibitor oryzacystatin I showed doubled proteolytic activity and were heavier than those reared on the corresponding *B.napus* wild type (Girard et al. 1998). This unexpected result shows that *Ps.chrysocephala* is not only adapted to glucosinolates but also to plant proteinase inhibitors.

Specialist chrysomelids are well known for discriminating between crucifer species (Feeny et al. 1970; Nielsen 1977; Bartlet and Williams 1991; Pachagounder and Lamb 1998; Pachagounder et al. 1998), but the factors that determine host suitability and preference are often not understood. Although leaf beetles recognise and differentially respond to individual glucosinolates when offered in isolation, there is little evidence that host plant preference relies on specific glucosinolate profiles (Nielsen 1988). Instead, the presence of other toxic secondary metabolites such as cucurbitacins and cardenolides was shown to affect host suitability for *Phyllotreta* spp. and *Phaedoncochleariae* (abbreviated *Phaedon*) (Nielsen 1978). Toxic cucurbitacins B, E, and I present in *Iberis* spp. deterred feeding of *Phyllotretanemorum* but not of *Phaedon*, an effect that correlated with their feeding behavior towards *Iberis* plants. On the other hand, *Phaedon*, *Phyllotretaundulata*, and *Phyllotretatetrastigma* did not feed on cardenolide-containing *Cheiranthus* and *Erysimum* spp., which are accepted as food plants by *Ph.nemorum* (Nielsen 1978).

The oligophagous species *Ph.nemorum* is used as a model to study the genetic basis of host plant adaptation. The common wild crucifer, Barbareavulgarisssp.arcuata (abbreviated *B.vulgaris*), is an atypical host plant for *Ph.nemorum*. However, the discovery of two different flea beetle populations using *B.vulgaris* as natural host plant suggests that *Ph.nemorum* is extending its host plant range to include *B.vulgaris* in Denmark (Nielsen 1996; de Jong et al. 2000). There are two distinct types of *B.vulgaris*. The so-called P-type with pubescent leaves is susceptible to all *Ph.nemorum* genotypes, whereas the G-type with glabrous leaves is resistant to most *Ph.nemorum* genotypes (Nielsen 1997b). The flea beetle-resistant G-type represents the common *B.vulgaris* genotype in Western Europe, while the P-type is rare (Hauser et al. 2012; Christensen et al. 2014).

The two *B.vulgaris* types differ not only morphologically but also regarding their chemical defences, i.e. glucosinolates and saponins. Feeding assays showed that susceptible *Ph.nemorum* larvae started to mine into the leaves of the G-type, but then either left and refused to feed or died in the mine, showing that the G-type is toxic for them (Nielsen 1997a, 1997b). Resistance of the G-type to *Ph.nemorum* is linked to the presence of the triterpenoid saponins hederagenin cellobioside, oleanolic acid cellobioside, gypsogenin cellobioside, and 4-epihederagenin cellobioside, and not to distinct glucosinolate profiles (Agerbirk et al. 2001; Kuzina et al. 2009; Nielsen et al. 2010). The toxicity of saponins is at least partially due to their interactions with cell membranes, which can cause cell death (Augustin et al. 2011). The activity of isolated hederagenin cellobioside and oleanolic acid cellobioside was tested separately in no-choice feeding assays with *Ph.nemorum* adults from five different near-isogenic lines (Nielsen et al. 2010). In these experiments, hederagenin cellobioside had a much stronger negative effect on adult feeding than oleanolic acid cellobioside, whereas the corresponding aglycones of both saponins were not active. An even stronger negative effect on some *Ph.nemorum* lines was observed for α-hederin, a saponin which is not present in *B.vulgaris*, and only differs from hederagenin cellobioside in its glycosylation pattern (Nielsen et al. 2010). These results show that aglycone structure as well as glycosylation pattern affect the biological activity of saponins towards *Ph.nemorum*.

Although the saponin-based defence of *B.vulgaris* is a dead-end for most *Ph.nemorum* genotypes, resistant individuals that performed well on the G-type were found at varying frequencies in all sampled populations (Nielsen and de Jong 2005; Nielsen 2012; Vermeer et al. 2012). The ability to use the G-type as a host plant clearly shows that resistant individuals can tolerate or detoxify saponins by an unknown mechanism. In genetic analyses, Nielsen and de Jong identified the presence of dominant resistance-conferring genes (R-genes) in all resistant individuals, but divergent modes of inheritance of these R-genes (autosomal and sex-linked) between populations (Nielsen 1997a; de Jong et al. 2000; de Jong and Nielsen 2002; Nielsen 2012). For example, in the resistant population from Ejby (Denmark), two major R-genes were linked to the sex chromosomes with additional autosomal R-genes. In a resistant population from Kværkeby (Denmark), most individuals were homozygous for a single autosomal R-gene (Nielsen 1997a; de Jong et al. 2000). In crossing experiments with resistant males from a Swiss population, an autosomal R-gene was inherited only to female offspring due to non-random segregation. The most likely explanation for this non-random segregation of the autosomal R-gene together with the X chromosome is the fusion of an autosome carrying the susceptible allele to the Y-chromosome in Swiss males (Nielsen 2012). When this R-gene was introduced into the genetic background of the susceptible *Ph.nemorum* line, it showed a normal Mendelian inheritance pattern (Nielsen 2012). These results strongly suggest that the genetic architecture of *Ph.nemorum* males differs among flea beetle populations, and that this polymorphism affects the inheritance of R-genes that enable the offspring to use the otherwise toxic *B.vulgaris* G-type as a host plant. Interestingly, attempts to generate *Ph.nemorum* lines that are homozygous for an autosomal R-gene resulted in very low survival rated of the homozygous larvae (de Jong and Nielsen 2000; Breuker et al. 2007). This observation was surprising as the homozygous resistant genotype was common at least in the *B.vulgaris*-feeding population from Kværkeby, which suggests that co-adapted genes present in the field population counteract the fitness cost of R-genes (de Jong et al. 2000; de Jong and Nielsen 2002). The genetic diversity and population structure of *Ph.nemorum* makes this species an ideal model to study the genetic basis of host range expansion in an oligophagous herbivore.

## Conclusions and future directions

The flea beetle genera *Psylliodes* and *Phyllotreta* are closely associated with glucosinolate-containing plants mainly in the family Brassicaceae. Nevertheless, they differ remarkably in their overall host plant use and their adaptations to glucosinolates, the characteristic defence metabolites in Brassicaceae. While *Ph.striolata* can utilise sequestered glucosinolates for its defence against predators, *Ps.chrysocephala* apparently does not possess endogenous myrosinase activity and accumulates much lower amounts of glucosinolates compared to *Ph.striolata*. In addition, both species differ regarding their ability to detoxify glucosinolates by desulfation (Beran et al. 2014, 2018).

Despite this progress, our knowledge on the adaptations of *Phyllotreta* and *Psylliodes* to the glucosinolate-myrosinase defence is far from complete. It is unknown, for instance, whether *Phyllotreta* rapidly sequester glucosinolates to prevent their breakdown to toxic isothiocyanates, and whether *Phyllotreta* gain protection from natural enemies by activating sequestered glucosinolates using their own myrosinase. In *Ps.chrysocephala*, the importance of the various detoxification strategies and their evolution needs to be investigated. To this end, a robust phylogenetic tree of the genus and comparative studies on how other *Psylliodes* species are processing dietary glucosinolates are necessary.

A future goal is to place adaptations of *Phyllotreta* and *Psylliodes* to their glucosinolate-containing host plants into a broader evolutionary context. While recent phylogenetic studies support the hypothesis that both genera adapted independently to Brassicaceae, their relationships to other genera of Alticini remain largely unresolved (Ge et al. 2011; Ge et al. 2012; Nie et al. 2018). At this background, a comprehensive and well-resolved phylogenetic tree of the tribe Alticini will enable studies on interactions with plants in general and adaptations to plant chemical defences, and how they contributed to the evolutionary success of this megadiverse lineage.

## Supplementary materials

Supplementary material 1 Table S1 Data type: (Species, host plants, diet breadth, geographic distribution) Explanation note: List of *Psylliodes* species according to their subgenera, including their food plants, diet breadth and geographical distribution. This dataset is made available under the Open Database License (http://opendatacommons.org/licenses/odbl/1.0/). The Open Database License (ODbL) is a license agreement intended to allow users to freely share, modify, and use this Dataset while maintaining this same freedom for others, provided that the original source and author(s) are credited.Matilda W. Gikonyo, Maurizio Biondi, Franziska Beran

Supplementary material 2 Table S2 Data type: (Species, host plant families) Explanation note: Species groups of *Psylliodes* s. str. and their associated host plant families. This dataset is made available under the Open Database License (http://opendatacommons.org/licenses/odbl/1.0/). The Open Database License (ODbL) is a license agreement intended to allow users to freely share, modify, and use this Dataset while maintaining this same freedom for others, provided that the original source and author(s) are credited.Matilda W. Gikonyo, Maurizio Biondi, Franziska Beran

Supplementary material 3 Table S3 Data type: (Species, host plants, diet breadth, geographic distribution) Explanation note: List of *Phyllotreta* species including their food plants, diet breadth and geographical distribution. This dataset is made available under the Open Database License (http://opendatacommons.org/licenses/odbl/1.0/). The Open Database License (ODbL) is a license agreement intended to allow users to freely share, modify, and use this Dataset while maintaining this same freedom for others, provided that the original source and author(s) are credited.Matilda W. Gikonyo, Maurizio Biondi, Franziska Beran

## References

[B1] Agerbirk N Olsen CE (2012) Glucosinolate structures in evolution. Phytochemistry 77: 16–45. 10.1016/j.phytochem.2012.02.005 22405332

[B2] Agerbirk N Olsen CE Nielsen JK (2001) Seasonal variation in leaf glucosinolates and insect resistance in two types of Barbareavulgarisssparcuata. Phytochemistry 58: 91–100. 10.1016/S0031-9422(01)00151-0 11524118

[B3] Ameen AO (1996) The biology and ecology of the yellow-margined leaf beetle, *Microthecaochroloma* Stål, (Coleoptera: Chrysomelidae) on crucifers. LSU Historical Dissertations and Theses. 6173: https://digitalcommons.lsu.edu/gradschool_disstheses/6173

[B4] Antonini G Audisio P Biase AD Mancini E Rector BG Cristofaro M Biondi M Korotyaev BA Bon MC Konstantinov A Smith L (2008) The importance of molecular tools in classical biological control of weeds: two case studies with yellow starthistle candidate biocontrol agents. In: Julien MH Sforza R Bon MC Evans HC Hatcher PE Hinz HL Rector BG (Eds) Proceedings of the XII International Symposium on Biological Control of Weeds, La Grande Motte, France, 22–27 April 2007.C.A.B. International, Wallingford, Oxfordshire, UK, 263–269.

[B5] Augustin JM Kuzina V Andersen SB Bak S (2011) Molecular activities, biosynthesis and evolution of triterpenoid saponins. Phytochemistry 72: 435–457. 10.1016/j.phytochem.2011.01.015 21333312

[B6] Balsbaugh EU (1978) A second species of *Microtheca* Stål (Coleoptera: Chrysomelidae found in North America. The Coleopterist Bulletin 32: 219–222.

[B7] Balusu RR Rhodes EM Majumdar A Cave RD Liburd OE Fadamiro HY (2017) Biology, ecology, and management of *Microthecaochroloma* (Coleoptera: Chrysomelidae) in organic crucifer production. Journal of Integrated Pest Management 8: 1–10. 10.1093/jipm/pmx007

[B8] Bartlet E Kiddle G Williams I Wallsgrove R (1999) Wound-induced increases in the glucosinolate content of oilseed rape and their effect on subsequent herbivory by a crucifer specialist. Entomologia Experimentalis et Applicata 91: 163–167. 10.1046/j.1570-7458.1999.00479.x

[B9] Bartlet E Mithen R Clark SJ (1996) Feeding of the cabbage stem flea beetle *Psylliodeschrysocephala* on high and low glucosinolate cultivars of oilseed rape. Entomologia Experimentalis et Applicata 80: 87–89. 10.1111/j.1570-7458.1996.tb00892.x

[B10] Bartlet E Parsons D Williams IH Clark SJ (1994) The influence of glucosinolates and sugars on feeding by the cabbage stem flea beetle, *Psylliodeschrysocephala*. Entomologia Experimentalis et Applicata 73: 77–83. 10.1111/j.1570-7458.1994.tb01841.x

[B11] Bartlet E Williams IH (1991) Factors restricting the feeding of the cabbage stem flea beetle (*Psylliodeschrysocephala*). Entomologia Experimentalis et Applicata 60: 233–238. 10.1111/j.1570-7458.1991.tb01543.x

[B12] Bartlet E Williams IH Blight MM Hick AJ (1992) Responses of the oilseed rape pests, *Ceutorhynchusassimilis* and *Psylliodeschrysocephala*, to a mixture of isothiocyanates. In: Menken SBJ Visser JH Harrewijn P (Eds) Proceedings of the 8th International Symposium on Insect-Plant Relationships.Kluwer Academic Publishers, Dordrecht, 103–104. 10.1007/978-94-011-1654-1_29

[B13] Baviera C Biondi M (2015) The Alticini (Coleoptera: Chrysomelidae, Galerucinae) of Sicily: Recent records and updated checklist. Atti della Accademia Peloritana dei Pericolanti, Classe di Scienze Fisiche, Matematiche e Naturali 93: A2-A50. 10.1478/AAPP.932A2

[B14] Bechyné J (1997) Evaluación de los datos sobre los Phytophaga dañinos en Venezuela (Coleoptera). Parte II. In: Savini V (Ed.) Boletín de Entomología Venezolana. Serie Monografias No 1. Aragua, Venezuela, 459 pp.

[B15] Beran F (2011) Host preference and aggregation behavior of the striped flea beetle, *Phyllotretastriolata*. Berliner ökophysiologische und phytomedizinische Schriften. Der Andere Verlag, Uelvesbüll, 143 pp.

[B16] Beran F Pauchet Y Kunert G Reichelt M Wielsch N Vogel H Reinecke A Svatos A Mewis I Schmid D Ramasamy S Ulrichs C Hansson BS Gershenzon J Heckel DG (2014) *Phyllotretastriolata* flea beetles use host plant defense compounds to create their own glucosinolate-myrosinase system. Proceedings of the National Academy of Sciences of the United States of America 111: 7349–7354. 10.1073/pnas.1321781111 24799680 PMC4034198

[B17] Beran F Sporer T Paetz C Ahn SJ Betzin F Kunert G Shekhov A Vassao DG Bartram S Lorenz S Reichelt M (2018) One pathway is not enough: The cabbage stem flea beetle *Psylliodeschrysocephala* uses multiple strategies to overcome the glucosinolate-myrosinase defense in its host plants. Frontiers in Plant Science 9. 10.3389/fpls.2018.01754 PMC6292997 30581445

[B18] Bieńkowski AO Orlova-Bienkowskaja MJ (2015) Trophic specialization of leaf beetles (Coleoptera, Chrysomelidae) in the Volga Upland. Biology Bulletin 42: 863–869. 10.1134/S1062359015100015

[B19] Biondi M (1996) Proposal for an ecological and zoogeographical categorization of the Mediterranean species of the flea beetle genus *Longitarsus* Berthold. In: Jolivet PHA Cox ML (Eds) Chrysomelidae Biology Volume 3: General Studies.SPB Academic Publishing bv, Amsterdam, 13–35.

[B20] Biondi M D’Alessandro P (2018) Two new species of the flea beetle genus *Psylliodes* Latreille of the *montana* species-group from Eastern Africa (Coleoptera: Chrysomelidae). Fragmenta Entomologica 50: 87–93. 10.4081/fe.2018.305

[B21] Breuker CJ de Jong PW Victoir K Vrieling K Brakefield PM (2007) Pleiotropic effects associated with an allele enabling the flea beetle *Phyllotretanemorum* to use *Barbareavulgaris* as a host plant. Evolutionary Ecology 21: 13–26. 10.1007/s10682-006-9121-0

[B22] Brown KK Hampton MB (2011) Biological targets of isothiocyanates. Biochimica Et Biophysica Acta-General Subjects 1810: 888–894. 10.1016/j.bbagen.2011.06.004 21704127

[B23] Cabrera N Rocca M (2012) First records of Chrysomelidae (Insecta, Coleoptera) on blueberries in Argentina: new associations between native chrysomelids and an exotic crop. Revista de la Sociedad Entomológica Argentina 71: 45–55.

[B24] Christensen S Heimes C Agerbirk N Kuzina V Olsen CE Hauser TP (2014) Different geographical distributions of two chemotypes of *Barbareavulgaris* that differ in resistance to insects and a pathogen. Journal of Chemical Ecology 40: 491–501. 10.1007/s10886-014-0430-4 24777484

[B25] Clark SM LeDoux DG Seeno TN Riley EG Gilbert AJ Sullivan JM (2004) Host plants of leaf beetle species occurring in the United States and Canada (Coleoptera: Orsodacnidae, Megalopodidae, Chrysomelidae exclusive of Bruchinae). Coleopterist Society, Special Publication no. 2, 476 pp 10.1603/0013-8746(2005)098[0243:HPOLBS]2.0.CO;2

[B26] Cox ML (1998) The genus *Psylliodes* Latreille (Chrysomelidae: Alticinae) in the U.K.: with keys to the adults of all species and to the larvae of those species feeding on Brassicaceae. The Coleopterist 7: 33–65.

[B27] de Jong PW Frandsen HO Rasmussen L Nielsen JK (2000) Genetics of resistance against defences of the host plant *Barbareavulgaris* in a Danish flea beetle population. Proceedings of the Royal Society B-Biological Sciences 267: 1663–1670. 10.1098/rspb.2000.1193 PMC1690723 11467430

[B28] de Jong PW Nielsen JK (2000) Reduction in fitness of flea beetles which are homozygous for an autosomal gene conferring resistance to defences in *Barbareavulgaris*. Heredity 84: 20–28. 10.1046/j.1365-2540.2000.00613.x 10692007

[B29] de Jong PW Nielsen JK (2002) Host plant use of *Phyllotretanemorum*: do coadapted gene complexes play a role? Entomologia Experimentalis et Applicata 104: 207–215. 10.1046/j.1570-7458.2002.01008.x

[B30] Döberl M (2010) Subfamily Alticinae. In: Löbl I Smetana A (Eds) Catalogue of Palearctic Coleoptera.Apollo Books, Stenstrup, Denmark, 491–563.

[B31] Edger PP Heidel-Fischer HM Bekaert M Rota J Gloeckner G Platts AE Heckel DG Der JP Wafula EK Tang M Hofberger JA Smithson A Hall JC Blanchette M Bureau TE Wright SI dePamphilis CW Schranz ME Barker MS Conant GC Wahlberg N Vogel H Pires JC Wheat CW (2015) The butterfly plant arms-race escalated by gene and genome duplications. Proceedings of the National Academy of Sciences of the United States of America 112: 8362–8366. 10.1073/pnas.1503926112 26100883 PMC4500235

[B32] Ehrlich PR Raven PH (1964) Butterflies and plants - a study in coevolution. Evolution 18: 586–608. 10.2307/2406212

[B33] Feeny P Paauwe KL Demong NJ (1970) Flea beetles and mustard oils - host plant specificity of *Phyllotretacruciferae* and *P.striolata* adults (Coleoptera: Chrysomelidae). Annals of the Entomological Society of America 63: 832–841. 10.1093/aesa/63.3.832

[B34] Flowers RW Janzen DH (1997) Feeding records of Costa Rican leaf beetles (Coleoptera: Chrysomelidae). Florida Entomologist 80: 319–324. 10.2307/3495765

[B35] Forister ML Novotny V Panorska AK Baje L Basset Y Butterill PT Cizek L Coley PD Dem F Diniz IR Drozd P Fox M Glassmire AE Hazen R Hrcek J Jahner JP Kaman O Kozubowski TJ Kursar TA Lewis OT Lill J Marquis RJ Miller SE Morais HC Murakami M Nickel H Pardikes NA Ricklefs RE Singer MS Smilanich AM Stireman JO Villamarin-Cortez S Vodka S Volf M Wagner DL Walla T Weiblen GD Dyer LA (2015) The global distribution of diet breadth in insect herbivores. Proceedings of the National Academy of Sciences of the United States of America 112: 442–447. 10.1073/pnas.142304211 25548168 PMC4299246

[B36] Furth DG (1979) Zoogeography and host plant ecology of the alticinae of Israel, especially *Phyllotreta*; with descriptions of three new species (Coleoptera: Chrysomelidae). Israel Journal of Entomology 28: 1–37.

[B37] Furth DG (1983) Alticinae of Israel: *Psylliodes* (Coleoptera: Chrysomelidae). Israel Journal of Entomology 17: 37–58.

[B38] Futuyma DJ Agrawal AA (2009) Macroevolution and the biological diversity of plants and herbivores. Proceedings of the National Academy of Sciences of the United States of America 106: 18054–18061. 10.1073/pnas.0904106106 19815508 PMC2775342

[B39] Gavrilović B Gavrilović B Ćurčić S Stojanović D Savić D (2014) Leaf beetles (coleoptera: Chrysomelidae) of Mt. Fruška Gora (Vojvodina Province, Northern Serbia), with an overview of host plants. Šumarski list 138: 29–41.

[B40] Ge D Chesters D Gómez-Zurita J Zhang L Yang X Vogler AP (2011) Anti-predator defence drives parallel morphological evolution in flea beetles. Proceedings of The Royal Society B – Biological sciences 278: 2133–2141. 10.1098/rspb.2010.1500 PMC3107618 21159678

[B41] Ge D Gómez-Zurita J Chesters D Yang X Vogler AP (2012) Suprageneric systematics of flea beetles (Chrysomelidae: Alticinae) inferred from multilocus sequence data. Molecular Phylogenetics and Evolution 62: 793–805. 10.1016/j.ympev.2011.11.028 22197803

[B42] Ge S-Q Daccordi M Ren J Li W-Z Yang X-K (2013) *Odontoedon*, a new genus from China with descriptions of nine new species (Coleoptera: Chrysomelidae: Chrysomelinae). Stuttgarter Beiträge zur Naturkunde A Neue Serie 6: 199–222.

[B43] Ge S-Q Daccordi M Ren J Yang X-K (2015) Revision of *Phaedon* Latreille from China (Coleoptera: Chrysomelidae) . Zoological Systematics 40: 1–30. 10.11865/zs.20150101

[B44] Ge S-Q Daccordi M Wang S-Y Yang X-K (2009) Study of the Genus *Entomoscelis* Chevrolat (Coleoptera: Chrysomelidae: Chrysomelinae) from China. Proceedings of the Entomological Society of Washington 111: 410–425. 10.4289/0013-8797-111.2.410

[B45] Ge S-Q Yang XK Cui JZ (2003) A key to the genus *Phaedon* (Coleoptera: Chrysomelidae: Chrysomelinae) from China and the description of a new species. Entomological News 114: 75–80.

[B46] Gerber GH (1994) Biology of *Entomoscelis* Chevrolat. In: Jolivet PH Cox ML Petitpierre E (Eds) Novel aspects of the biology of Chrysomelidae.Kluwer Academic Publishers, Dordrecht, 549–553. 10.1007/978-94-011-1781-4_42

[B47] Girard C Le Metayer M Zaccomer B Bartlet E Williams I Bonade-Bottino M Pham-Delegue MH Jouanin L (1998) Growth stimulation of beetle larvae reared on a transgenic oilseed rape expressing a cysteine proteinase inhibitor. Journal of Insect Physiology 44: 263–270. 10.1016/S0022-1910(97)00142-X 12769960

[B48] Gómez-Zurita J Juan C Petitpierre E (2000a) The evolutionary history of the genus *Timarcha* (Coleoptera, Chrysomelidae) inferred from mitochondrial COII gene and partial 16S rDNA sequences. Molecular Phylogenetics and Evolution 14: 304–317. 10.1006/mpev.1999.0712 10679162

[B49] Gómez-Zurita J Juan C Petitpierre E (2000b) Sequence, secondary structure and phylogenetic analyses of the ribosomal internal transcribed spacer 2 (ITS2) in the *Timarcha* leaf beetles (Coleoptera: Chrysomelidae). Insect Molecular Biology 9: 591–604. 10.1046/j.1365-2583.2000.00223.x 11122468

[B50] González-Megías A Gómez JM (2001) Adult and larval plant range and preference in *Timarchalugens* (Coleoptera: Chrysomelidae): strict monophagy on an atypical host. Annals of the Entomological Society of America 94: 110–115. 10.1603/0013-8746(2001)094[0110:AALPRA]2.0.CO;2

[B51] Görnitz K (1956) Weitere Untersuchungen über Insekten-Attraktivstoffe aus Cruciferen. Nachrichtenblatt Deutscher Pflanzenschutzdienst 10: 137–147

[B52] Hauser TP Toneatto F Nielsen JK (2012) Genetic and geographic structure of an insect resistant and a susceptible type of *Barbareavulgaris* in western Europe. Evolutionary Ecology 26: 611–624. 10.1007/s10682-011-9515-5

[B53] Heckel DG (2014) Insect detoxification and sequestration strategies. In: Voelckel C Jander G (Eds) Annual Plant Reviews; Insect-Plant Interactions.Wiley-Blackwell, Chichester, 77–114. 10.1002/9781118472507.Ch3

[B54] Heikertinger F (1943) Die *Phyllotreta*-Arten des äthiopischen Faunengebietes. Arbeiten über morphologische und taxonomische Entomologie aus Berlin-Dahlem 10: 33–56.

[B55] Hicks KL (1974) Mustard oil glucosides: feeding stimulants for adult cabbage flea beetles, *Phyllotretacruciferae* (Coleoptera: Chrysomelidae). Annals of the Entomological Society of America 67: 261–264. 10.1093/aesa/67.2.261

[B56] Hill DS (2008) Pests of crops in warmer climates and their control. Springer, Netherlands, 708 pp 10.1017/CBO9781107415324.004

[B57] Jeschke V Gershenzon J Vassão DG (2016) Insect detoxification of glucosinolates and their hydrolysis products. In: Kopriva S (Ed.) Advances in Botanical Research - Glucosinolates.Elsevier, Amsterdam, 199–245. 10.1016/bs.abr.2016.06.003

[B58] Jolivet P (1951) Contribution a l’etude des *Microtheca* Stål (ColeopteraChrysomelidae) (2^eme^ Note). Institut royal des sciences naturelles de Belgique 27: 1–7.

[B59] Jolivet P (1991) Sélection trophique chez les *Alticinae* (ColeopteraChrysomelidae). Bulletin mensuel de la Société Linnéenne de Lyon 60: 26–40. 10.3406/linly.1991.10916

[B60] Jolivet P Hawkeswood T (1995) Host-plants of Chrysomelidae of the world: an Essay about the relationships between the leaf-beetles and their Food-plants. Backhuys Publishers, Leiden, Netherlands, 281 pp.

[B61] Jolivet P Petitpierre E (1973) Plantes-hôtes connues des *Timarcha* Latreille. Bulletin de la Société entomologique de France 78: 9–25.

[B62] Jolivet P Petitpierre E (1976a) Les plantes-hotes connues des *Chrysolina* (Col. Chrysomelidae) Essai sur les types de selection trophique. Annales de la Société Entomologique de France 12: 123–149.

[B63] Jolivet P Petitpierre E (1976b) Selection trophique et evolution chromosomique chez les Chrysomelinae (Col. Chrysomelidae). Acta zoologica et pathologica antverpiensia 66: 59–90.

[B64] Jurado-Rivera JA Petitpierre E (2015) New contributions to the molecular systematics and the evolution of host-plant associations in the genus *Chrysolina* (Coleoptera, Chrysomelidae, Chrysomelinae). In: Jolivet P; Santiago-Blay, J; Schmitt, M (Eds) Research on Chrysomelidae 5 - ZooKeys 547: 165–192. 10.3897/zookeys.547.6018 PMC4714339 26798320

[B65] Knodel JJ (2017) Flea Beetles (*Phyllotreta* spp.) and Their Management. In: Reddy GVP (Ed.) Integrated Management of Insect Pests on Canola and Other Brassica Oilseed Crops.CAB International, Oxfordshire, 1–12. 10.1079/9781780648200.0001

[B66] Konstantinov AS Vandenberg NJ (1996) Handbook of Palearctic flea beetles (Coleoptera: Chrysomelidae: Alticinae). In: Gupta VK (Ed.) Contributions on Entomology, International.Associated Publishers, Gainesville, FL, 237–437.

[B67] Kuzina V Ekstrøm CT Andersen SB Nielsen JK Olsen CE Bak S (2009) Identification of defense compounds in *Barbareavulgaris* against the herbivore *Phyllotretanemorum* by an ecometabolomic approach. Plant Physiology 151: 1977–1990. 10.1104/pp.109.136952 19819983 PMC2785962

[B68] Lamb RJ (1989) Entomology of oilseed *Brassica* crops. Annual Review of Entomology 34: 211–229. 10.1146/annurev.en.34.010189.001235

[B69] Leonardi C (1970) Materiali per uno studio filogenetico del genere *Psylliodes*ColeopteraChrysomelidae). Atti della Società italiana di scienze naturali e del Museo civico di storia naturale di Milano 110: 201–223.

[B70] Letsch H Gottsberger B Metzl C Astrin J Friedman ALL McKenna DD Fiedler K (2018) Climate and host-plant associations shaped the evolution of ceutorhynch weevils throughout the Cenozoic. Evolution 72: 1815–1828. 10.1111/evo.13520 30040114 PMC6175111

[B71] Löbl I Smetana A (2010) Catalogue of Palaearctic Coleoptera, Volume 6. Chrysomeloidea. Apollo Books, Stenstrup, Denmark, 924 pp.

[B72] Lopatin IK (2005) New species of leaf-beetles (Coleoptera, Chrysomelidae) from China: V. Entomological Review 85: 934–939.

[B73] Menezes AO Mikami AY Ide AK Ventura MU (2005) Feeding preferences of *Microthecapunctigera* (Achard) (Coleoptera: Chrysomelidae) for some Brassicaceae plants in multiple-choice assays. Scientia Agricola 62: 72–75. 10.1590/S0103-90162005000100014

[B74] Milliron HE (1953) A European flea beetle injuring crucifers in North America. Journal of Economic Entomology 46: 179–179. 10.1093/jee/46.1.179

[B75] Mitchell-Olds T Siemens D Pedersen D (1996) Physiology and costs of resistance to herbivory and disease in *Brassica*. Entomologia Experimentalis et Applicata 80: 231–237. 10.1111/j.1570-7458.1996.tb00925.x

[B76] Mitchell BK (1978) Some aspects of gustation in the larval red turnip beetle, *Entomoscelisamericana*, related to feeding and host plant selection. Entomologia Experimentalis et Applicata 24: 540–549. 10.1111/j.1570-7458.1978.tb02815.x

[B77] Mitchell BK (1988) Adult leaf beetles as models for exploring the chemical basis of host-plant recognition. Journal of Insect Physiology 34: 213–225. 10.1016/0022-1910(88)90052-2

[B78] Mitchell BK (1994) The chemosensory basis of host-plant recognition in Chrysomelidae. In: Jolivet PH Cox ML Petitpierre E (Eds) Novel aspects of the biology of Chrysomelidae.Kluwer Academic Publishers, Dordrecht, 141–151. 10.1007/978-94-011-1781-4_7

[B79] Mithöfer A Boland W (2012) Plant defense against herbivores: chemical aspects. Annual Review of Plant Biology 63: 431–450. 10.1146/annurev-arplant-042110-103854 22404468

[B80] Mohr KH (1966) Chrysomelidae. In: Freude H Harde KW Lohse GA (Eds) Die Käfer Mitteleuropas.Goecke & Evers Verlag, Krefeld, 95–280.

[B81] Nadein KS (2006) A significance of the tegmen structure for classification of the genus *Psylliodes* Latreille, 1829 (Coleoptera: Chrysomelidae: Psylliodina). Proceedings of the Russian Entomological Society St. Petersburg 77: 250–254.

[B82] Nadein KS (2007a) On the taxonomy and classification of the genus *Psylliodes* Latreille, 1825 (Coleoptera, Chrysomelidae, Galerucinae). Entomologica Basiliensia et Collectionis Frey 29: 307–332.

[B83] Nadein KS (2007b) A review of the leaf-beetle genus *Psylliodes* Latreille (Coleoptera, Chrysomelidae) from Russia and neighboring countries: I. A key to subgenera, species-groups, and species. Entomological Review 87: 330–360. 10.1134/S0013873807030086

[B84] Nadein KS (2010) A review of the genus *Psylliodes* Latreille (Coleoptera, Chrysomelidae) from Russia and neighboring countries: II. An annotated list of species. Entomological Review 90: 1035–1074. 10.1134/S0013873810080099

[B85] Nadein KS (2012) Catalogue of Alticini genera of the World (Coleoptera: Chrysomelidae). Beetles and Coleopterists website, Zoological Institute, Saint-Petersburg. http://www.zin.ru/Animalia/Coleoptera/eng/alticinw.htm [accessed 02.01.2019]

[B86] Nadein KS Bezděk J (2014) Galerucinae Latreille, 1802. In: Leschen RAB Beutel RG (Eds) Handbook of Zoology.Coleoptera, beetles. Morphology and Systematics. Volume 3. Walter De Gruyter, Berlin/Boston, 251–259.

[B87] Nie RE Breeschoten T Timmermans MJTN Nadein K Xue HJ Bai M Huang Y Yang XK Vogler AP (2018) The phylogeny of Galerucinae (Coleoptera: Chrysomelidae) and the performance of mitochondrial genomes in phylogenetic inference compared to nuclear rRNA genes. Cladistics 34: 113–130. 10.1111/cla.12196 34645082

[B88] Nielsen JK (1977) Host plant relationships of *Phyllotretanemorum* L (Coleoptera: Chrysomelidae). 1. Field Studies. Journal of Applied Entomology 84: 396–407. 10.1111/j.1439-0418.1977.tb04301.x

[B89] Nielsen JK (1978) Host plant discrimination within cruciferae: feeding responses of four leaf beetles (Coleoptera: Chrysomelidae) to glucosinolates, cucurbitacins and cardenolides. Entomologia Experimentalis et Applicata 24: 41–54. 10.1111/j.1570-7458.1978.tb02755.x

[B90] Nielsen JK (1988) Crucifer-feeding Chrysomelidae: mechanisms of host plant finding and acceptance. In: Jolivet PH Petitpierre E Hsiao TH (Eds) Biology of Chrysomelidae.Kluwer Academic Publishers, Dordrecht, 25–40. 10.1007/978-94-009-3105-3_2

[B91] Nielsen JK (1996) Intraspecific variability in adult flea beetle behaviour and larval performance on an atypical host plant. Entomologia Experimentalis et Applicata 80: 160–162. 10.1111/j.1570-7458.1996.tb00909.x

[B92] Nielsen JK (1997a) Genetics of the ability of *Phyllotretanemorum* larvae to survive in an atypical host plant, Barbareavulgarisssparcuata. Entomologia Experimentalis et Applicata 82: 37–44. 10.1046/j.1570-7458.1997.00111.x

[B93] Nielsen JK (1997b) Variation in defences of the plant *Barbareavulgaris* and in counteradaptations by the flea beetle *Phyllotretanemorum*. Entomologia Experimentalis et Applicata 82: 25–35. 10.1046/j.1570-7458.1997.00110.x

[B94] Nielsen JK (2012) Non-random segregation of an autosomal gene in males of the flea beetle, *Phyllotretanemorum*: implications for colonization of a novel host plant. Entomologia Experimentalis et Applicata 143: 301–312. 10.1111/j.1570-7458.2012.01262.x

[B95] Nielsen JK de Jong PW (2005) Temporal and host-related variation in frequencies of genes that enable *Phyllotretanemorum* to utilize a novel host plant, *Barbareavulgaris*. Entomologia Experimentalis et Applicata 115: 265–270. 10.1111/j.1570-7458.2005.00293.x

[B96] Nielsen JK Nagao T Okabe H Shinoda T (2010) Resistance in the plant, *Barbareavulgaris*, and counter-adaptations in flea beetles mediated by saponins. Journal of Chemical Ecology 36: 277–285. 10.1007/s10886-010-9758-6 20177743

[B97] Pachagounder P Lamb RJ (1998) Feeding preferences of a flea beetle, *Phyllotretacruciferae* (Coleoptera: Chrysomelidae), among wild crucifers. The Canadian Entomologist 130: 241–242. 10.4039/Ent130241-2

[B98] Pachagounder P Lamb RJ Bodnaryk RP (1998) Resistance to the flea beetle *Phyllotretacruciferae* (Coleoptera: Chrysomelidae) in false flax, *Camelinasativa* (Brassicaceae). The Canadian Entomologist 130: 235–240. 10.4039/Ent130235-2

[B99] Pentzold S Jensen MK Matthes A Olsen CE Petersen BL Clausen H Moller BL Bak S Zagrobelny M (2017) Spatial separation of the cyanogenic β-glucosidase ZfBGD2 and cyanogenic glucosides in the haemolymph of *Zygaena* larvae facilitates cyanide release. Royal Society Open Science 4: 170262. 10.1098/rsos.170262 PMC5493921 28680679

[B100] Pivnick KA Lamb RJ Reed D (1992) Response of flea beetles, *Phyllotreta* spp, to mustard oils and nitriles in field trapping experiments. Journal of Chemical Ecology 18: 863–873. 10.1007/BF00988327 24254090

[B101] Ratzka A Vogel H Kliebenstein DJ Mitchell-Olds T Kroymann J (2002) Disarming the mustard oil bomb. Proceedings of the National Academy of Sciences, USA 99: 11223–11228. 10.1073/pnas.172112899 PMC123237 12161563

[B102] Reifenrath K Müller C (2008) Multiple feeding stimulants in *Sinapisalba* for the oligophagous leaf beetle *Phaedoncochleariae*. Chemoecology 18: 19–27. 10.1007/s00049-007-0389-5

[B103] Rheinheimer J Hassler M (2018) Die Blattkäfer Baden-Württembergs. Kleinsteuber Books, Karlsruhe, 928 pp.

[B104] Schoonhoven LM Van Loon JJA Dicke M (2005) Insect-Plant Biology. Oxford University Press, Oxford, 421 pp.

[B105] Siemens DH Mitchell-Olds T (1996) Glucosinolates and herbivory by specialists (Coleoptera: Chrysomelidae, Lepidoptera: Plutellidae): Consequences of concentration and induced resistance. Environmental Entomology 25: 1344–1353. 10.1093/ee/25.6.1344

[B106] Smith EH (1985) Revision of the genus *Phyllotreta* Chevrolat of America North of Mexico. Part I. The maculate species (Coleoptera: Chrysomelidae: Alticinae). Fieldiana Zoology, 168 pp. 10.5962/bhl.title.3408

[B107] Tóth M Csonka É Bakcsa F Benedek P Szarukán I Gomboc S Toshova T Subchev M Ujváry I (2007) Species spectrum of flea beetles (*Phyllotreta* spp., Coleoptera, Chrysomelidae) attracted to allyl isothiocyanate-baited traps. Zeitschrift für Naturforschung 62c: 772–778. 10.1515/znc-2007-9-1022 18069253

[B108] Vermeer KMCA Verbaarschot P de Jong PW (2012) Changes in frequencies of genes that enable *Phyllotretanemorum* to utilize its host plant, *Barbareavulgaris*, vary in magnitude and direction, as much within as between seasons. Entomologia Experimentalis et Applicata 144: 37–44. 10.1111/j.1570-7458.2012.01241.x

[B109] Vig K (1996) Host plant selection by *Phyllotretavittula* (Redtenbacher, 1849). Proceedings of the Fourth International Symposium on the Chrysomelidae, Proceedings of XX ICE, Firenze. Museo Regionale di Scienze Naturali, Torino, 233–251.

[B110] Wheat CW Vogel H Wittstock U Braby MF Underwood D Mitchell-Olds T (2007) The genetic basis of a plant-insect coevolutionary key innovation. Proceedings of the National Academy of Sciences of the United States of America 104: 20427–20431. 18077380 10.1073/pnas.0706229104 PMC2154447

[B111] Winde I Wittstock U (2011) Insect herbivore counteradaptations to the plant glucosinolate-myrosinase system. Phytochemistry 72: 1566–1575. 10.1016/j.phytochem.2011.01.016 21316065

[B112] Wittstock U Agerbirk N Stauber EJ Olsen CE Hippler M Mitchell-Olds T Gershenson J Vogel H (2004) Successful herbivore attack due to metabolic diversion of a plant chemical defense. Proceedings of the National Academy of Sciences, USA 101: 4859–4864. 10.1073/pnas.0308007101 PMC387339 15051878

[B113] Wittstock U Kurzbach E Herfurh A-M Stauber EJ (2016) Glucosinolate breakdown. In: Kopriva S (Ed.) Advances in Botanical Research – Glucosinolates.Elsevier, Amsterdam, 125–169. 10.1016/bs.abr.2016.06.006

[B114] Zimmer CT Müller A Heimbach U Nauen R (2014) Target-site resistance to pyrethroid insecticides in German populations of the cabbage stem flea beetle, *Psylliodeschrysocephala* L. (Coleoptera: Chrysomelidae). Pesticide Biochemistry and Physiology 108: 1–7. 10.1016/j.pestbp.2013.11.005 24485308

