# Supplementary material for: Adaptation of flea beetles to Brassicaceae: host plant associations and geographic distribution of Psylliodes Latreille and Phyllotreta Chevrolat (Coleoptera, Chrysomelidae)
Source: Zookeys. 2019 Jun 17;856:51–73. doi: 10.3897/zookeys.856.33724 (PMC6603994; doi:10.3897/zookeys.856.33724)
Supplement: Supplementary material 1 [file zookeys-856-051-s001.docx]

**Supplementary Table 1.** List of *Psylliodes* species according to their subgenera, including their food plants, diet breadth and geographical distribution.

| **Species** | **Food plants ^1^** | **Food plant family ^2^** | **Diet breadth ^3^** | **Location ^4^** | **Geographical Region** | **References ^5^** |
| --- | --- | --- | --- | --- | --- | --- |
| **Subgenus *Psyllobactra*** | | | | | | |
| *Psylliodes pubipennis*  Lopatin, 1958 | *Calophaca grandiflora* | Fabaceae | Unknown | Tajikistan (Southern) | PAR | Nadein 2010 |
| **Subgenus *Semicnema*** | | | | | | |
| *Psylliodes elongata*  Wang, 1992 | Unknown | Unknown | Unknown | China (Yunnan) | ORR | Döberl 2010 |
| *Psylliodes longicornis*  Wang, 1992 | Unknown | Unknown | Unknown | China (Yunnan) | ORR | Döberl 2010 |
| *Psylliodes macella*  Weise, 1900 | *Achnatherum splendens* | Poaceae | MON | Russia, Kazakhstan, Mongolia | PAR | Nadein 2010 |
| *Psylliodes reitteri parallela*  Weise, 1890 | *Phragmites* spp.*, Calamagrostis* spp.*, Miscanthus* spp*.* | Poaceae | OLI | Russia, Kyrgyzstan, Kazakhstan, Uzbekistan, Mongolia, Japan, China (Tibet) | PAR | Nadein 2010 |
| *Psylliodes reitteri reitteri*  Weise, 1888 | *Phragmites australis, Miscanthus* spp*.* | Poaceae | OLI | Austria, Germany, Russia, Ukraine, Czech Republic, Hungary, Bulgaria, Slovakia, Bosnia-Herzegovina | PAR | Fritzlar 2009; Nadein 2010; Rheinheimer and Hassler 2018 |
| **Subgenus *Eupus*** | | | | | | |
| *Psylliodes aemulans*  Lindberg, 1953 | Unknown | Unknown | Unknown | Spain (end. Canary Islands) | PAR | Biondi 1995 |
| *Psylliodes amplicollis*  Wollaston, 1856 | Unknown | Unknown | Unknown | Portugal (end. Madeira) | PAR | Döberl 2010 |
| *Psylliodes stolida*  Wollaston, 1860 | Unknown | Unknown | Unknown | Spain (only Canary Islands), Portugal (only Madeira) | PAR | Biondi 1995 |
| *Psylliodes tarsata*  Wollaston, 1854 | Unknown | Unknown | Unknown | Portugal (end. Madeira) | PAR | Döberl 2010 |
| *Psylliodes wollastoni*  Nadein, 2007 | Poaceae | Poaceae | Unknown | Portugal (end. Madeira) | PAR | Nadein 2007a; F. Beran (pers. comm) |
| **Subgenus *Minicnema*** | | | | | | |
| *Psylliodes belarbii*  Döberl, 1991 | Unknown | Unknown | Unknown | Morocco | PAR | Döberl 2010 |
| *Psylliodes elliptica*  Allard, 1861 | *Triticum aestivum, Hordeum vulgare* | Poaceae | OLI | Israel, Jordan, Syria, Turkey | PAR | Furth 1983; Leonardi 2007 |
| **Subgenus *Psylliodes* s. str** | | | | | | |
| *Psylliodes abdominalis*  Wang, 1992 | Unknown | Unknown | Unknown | Yunnan | ORR | Döberl 2010 |
| *Psylliodes aeneola*  Heikertinger, 1911 | Unknown | Unknown | Unknown | Kazakhstan | PAR | Döberl 2010 |
| *Psylliodes aerea*  Foudras, 1860 | *Thlaspi montanum, Arabis turrita, Alyssum murale, Crambe* spp.*, Iberis* spp. | Brassicaceae | OLI | Europe (Carpathians) | PAR | Doguet 1994; Aslan and Gök 2006; Nadein 2007a; 2010; Rheinheimer and Hassler 2018 |
| *Psylliodes affinis*  (Paykull, 1799) | *Solanum* spp.*, Hyosciamus niger, Atropa belladonna, Lycium halimifolium, L. barbarum, Scopolia carniolica, Nicotiana tabacum* | Solanaceae | OLI | Europe; Morocco, Kazakhstan, Russia, Turkey; Nearctic, Afrotropical Region | PAR, NAR, AFR | Clark et al. 2004; Nadein 2010; Rheinheimer and Hassler 2018; Doguet 1994 |
| *Psylliodes afromontana*  Biondi and D'Alessandro, 2018 | Unknown | Unknown | Unknown | Kenya | AFR | Biondi and D'Alessandro 2018; Döberl 2010 |
| *Psylliodes agropyri*  Palij, 1961 | *Agropyron* spp*., Achnatherum* spp*.* | Poaceae | OLI | Russia, Kazakhstan | PAR | Nadein 2010 |
| *Psylliodes algirica*  Allard, 1859 | *Quercus ilex* (reported also from *Populus alba* and *Pistacia lentiscus*) | Fagaceae | OLI | Greece, Spain, Italy, Algeria, Tunisia | PAR | Baviera and Biondi 2015; Heikertinger 1926; Petitpierre 1985; Petitpierre 1999 |
| *Psylliodes altimontana*  Medvedev, 2003 | Unknown | Unknown | Unknown | Nepal (Himalayas) | ORR | Döberl 2010; Medvedev 2004; Nadein 2007a |
| *Psylliodes amurensis*  Nadein, 2006 | Unknown | Unknown | Unknown | Russia (far East) | PAR | Döberl 2010 |
| *Psylliodes analogica*  Nadein, 2005 | Unknown | Unknown | Unknown | Kyrgyzstan | PAR | Döberl 2010 |
| *Psylliodes anatolica*  Gӧk and Cilbiroglu, 2004 | *Quercus* spp*.* | Fagaceae | MON | Turkey | PAR | Gök and Çilbiroğlu 2004; Şen and Gök 2009; I. Şen (pers. comm) |
| *Psylliodes angusticeps*  Israelson, 1980 | *Crambe strigosa* | Brassicaceae | MON | Spain (end. Canary Islands) | PAR | Biondi 1995; Nadein 2006; M. Biondi (pers. comm.) |
| *Psylliodes appalachiana*  Konstantinov and Tishechkin, 2004 | Unknown (leaf litter) | Unknown | Unknown | North America (Appalachian mountains) | NAR | Konstantinov and Tishechkin 2004 |
| *Psylliodes arida*  Lea, 1917 | Unknown | Unknown | Unknown | Australia | AUR | Lea 1917 |
| *Psylliodes arista*  Iablokoff-Khnzorian, 1962 | Unknown | Unknown | Unknown | Armenia, Georgia, Russia, Iran, Turkey | PAR | Döberl 2010 |
| *Psylliodes astenica*  Nadein, 2005 | Unknown | Unknown | Unknown | Tajikistan | PAR | Döberl 2010 |
| *Psylliodes attenuata*  (Koch, 1803) | *Humulus lupulus, H. japonicas, Cannabis sativa, Urtica dioica, Phaseolus* spp*., Solanum tuberosum, S. lycopersicum, Beta vulgaris, Linum* spp.*, Chenopodium* spp.*, Arctium lappa, A. tomentosum* | Cannabaceae, Fabaceae, Solanaceae, Asteraceae, Amaranthaceae, Linaceae | POL | Europe, China , Turkey, Uzbekistan, Mongolia, Kazakhstan, Japan, Russia, Taiwan | PAR, ORR | Doguet 1994;  Nadein 2010; Rheinheimer and Hassler 2018 |
| *Psylliodes ausoniae*  Leonardi, 2013 | *Quercus* spp., *Ostrya carpinifolia* | Fagaceae, Betulaceae | POL | Italy | PAR | Baviera and Biondi 2015 |
| *Psylliodes baligheensis*  Jacoby, 1896 | Unknown | Unknown | Unknown | Indonesia (Sumatra) | ORR | Jacoby 1896 |
| *Psylliodes baluchistana*  Lopatin, 1990 | Unknown | Unknown | Unknown | Iran | PAR | Döberl 2010 |
| *Psylliodes biondii*  Leonardi, 2007 | *Erysimum pseudorhaeticum, Isatis apennina* | Brassicaceae | OLI | Italy | PAR | Döberl 2010 |
| *Psylliodes brettinghami*  Baly, 1874 | *Solanum* spp.*, Physalis angulata, Physaliastrum japonicum* | Solanaceae | OLI | China ( Guangxi, Sichuan, Yunnan), Japan, Taiwan, Australia | PAR, ORR, AUR | Nadein and Lee 2012; Takizawa 2005 |
| *Psylliodes breweri*  Baly, 1876 | Unknown | Unknown | Unknown | Australia | AUR | Baly 1876 |
| *Psylliodes brisouti*  Bedel, 1898 | *Erysimum decumbens, E. ochroleucum, Arabis glabra* | Brassicaceae | OLI | Europe, Turkey | PAR | Doguet 1994; Brelih et al. 2003; Nadein 2010; Petitpierre 1999; Rheinheimer and Hassler 2018; Wanntorp and Ødegaard 2005 |
| *Psylliodes burangana*  Chen and Wang, 1981 | Unknown | Unknown | Unknown | China (Tibet) | PAR | Döberl 2010 |
| *Psylliodes caerulipes*  Jacoby, 1896 | Unknown | Unknown | Unknown | Indonesia (Sumatra) | ORR | Jacoby 1896 |
| *Psylliodes calcarata*  Bryant, 1944 | Unknown | Unknown | Unknown | Tanzania, Republic of South Africa | AFR | Biondi 1996 |
| *Psylliodes callinota*  Faldermann, 1837 | Unknown | Unknown | Unknown | Russia (Southern) | PAR | Döberl 2010 |
| *Psylliodes caneparii*  Leonardi, 2007 | *Erucastrum virgatum* | Brassicaceae | MON | Italy | PAR | Baviera and Biondi 2015 |
| *Psylliodes cantonensis*  *G*ruev, 1981 | Unknown (Recorded on *Clerodendron fortunatum* however, considering the close affinity of this species to *P. chlorophana*, the real host plant family of this species could be Solanaceae) | Unknown | Unknown | China (Guangdong), Taiwan | ORR | Gruev 1981; M. Biondi (pers. comm.) |
| *Psylliodes capitata*  Jacoby, 1892 | Unknown | Unknown | Unknown | Mexico (Guerrero) | NTR | Furth 2006 |
| *Psylliodes cerenae*  Gӧk et al., 2003 | Unknown | Unknown | Unknown | Turkey | PAR | Gök et al. 2003 |
| *Psylliodes cereola*  Peyerimhoff, 1925 | *Erucastrum elatum* | Brassicaceae | MON | Algeria, Morocco | PAR | Leonardi 2007 |
| *Psylliodes cervinoi*  Baselga and Novoa, 2003 | Unknown | Unknown | Unknown | Spain | PAR | Baselga and Novoa 2003b; Döberl 2010 |
| *Psylliodes chalcomera*  (Illiger, 1807) | *Carduus* spp.*, Cirsium* spp., *Centaurea solstitialis, C. cyanus, Helianthus annuus, Lactuca sativa, Onopordum acanthium, Carthamus tinctoria, Cynara cardunculus, Picnomon arcana* | Asteraceae | OLI | Europe, Algeria, Morocco, Tunisia, Russia, Kazakhstan, Turkey, Israel, Iran, Syria, China | PAR | Doguet 1994; Nadein 2010; Rheinheimer and Hassler 2018 |
| *Psylliodes chapuisii*  Baly, 1877 | Unknown | Unknown | Unknown | Malaysia (Peninsular) | ORR | Mohamedsaid 2004 |
| *Psylliodes chlorophana*  Jacoby, 1884 | *Solanum nigrum, S. americanum* | Solanaceae | MON | Japan, Taiwan, China; Oriental Region | PAR, ORR | Döberl 2010; Jacoby 1884; Scherer 1982 |
| *Psylliodes chrysocephala chrysocephala*  (Linnaeus, 1758) | *Brassica* spp., *Barbarea* spp., *Erucastrum* spp.*, Erysimum* spp.*, Sinapis* spp.*,*  *Rapistrum* spp., *Raphanus* spp., *Thlaspi arvense, Capsella bursa*-*pastoris*, *Alliaria petiolata*, *Sisymbrium officinale, Hirschfeldia* spp*., Isatis* spp., *Diplotaxis* spp. | Brassicaceae | OLI | Europe; Algeria, Morocco, Tunisia, Cyprus, Iran, Israel, Lebanon, Syria, Turkey, Syria, Jordan; Afrotropical; Nearctic | PAR, NAR, AFR | Döberl 2010; Nadein 2010; Rheinheimer and Hassler 2018; Doguet 1994 |
| *Psylliodes chrysocephala inops*  Peyerimhoff, 1915 | *Sinapis pubescens, Brassica gravinae* | Brassicaceae | OLI | Algeria | PAR | Döberl 2010; Peyerimhoff 1915 |
| *Psylliodes chujoe*  Madar, 1960 | *Cardamine anemonoides* | Brassicaceae | MON | Japan, Taiwan | PAR, ORR | Nadein and Lee 2012; Takizawa 2005 |
| *Psylliodes circumdata*  (Redtenbacher, 1842) | *Brassica nigra, Calepina irregularis, Bunias erucago,* (Collected from *Quercus* spp. as refugial plants) | Brassicaceae | OLI | Europe; Middle East; Algeria, Libya, Morocco, Tunisia. | PAR | Baviera and Biondi 2015; Doguet 1994; Doguet and Leonardi 2017; Furth 1983; Nadein 2010; Petitpierre 1999 |
| *Psylliodes coelestis*  Warchalowski, 2000 | Unknown | Unknown | Unknown | Spain, Morocco | PAR | Döberl 2010 |
| *Psylliodes concolor*  Nadein, 2006 | Unknown | Unknown | Unknown | Georgia | PAR | Döberl 2010; Nadein 2006 |
| *Psylliodes congrua*  Weise, 1923 | Unknown | Unknown | Unknown | Australia | AUR | Weise 1923 |
| *Psylliodes convexior*  LeConte, 1857 | *Descurainia pinnata, Dimorphocarpa wislizenii, Barbarea vulgaris, Brassica rapa, Capsella bursa-pastoris, Lepidium virginicum, Raphanus sativus, Humulus lupulus, Asclepias syriaca, Lactuca sativa, Beta vulgaris, Chenopodium album, Cyperaceae* spp*., Fabaceae* spp.*, Pinus ponderosa, Triticum aestivum, Zea mais* | Brassicaceae, Cannabaceae, Asclepiadaceae, Asteraceae, Amaranthaceae, Cyperaceae, Fabaceae,  Pinaceae,  Poaceae | POL | North America (Texas), Mexico (Baja California Sur) | NAR, NTR | Clark et al. 2004 |
| *Psylliodes crambicola*  Lohse, 1954 | *Crambe maritima, Isatis tinctoria* | Brassicaceae | OLI | Germany, Finland, Denmark, Norway, Russia, Sweden | PAR | Craven 2007; Nadein 2010; Rheinheimer and Hassler 2018 |
| *Psylliodes credens*  Fall, 1933 | Unknown (Recorded on *Salicornia* spp.) | Unknown | Unknown | North America (California) | NAR | Clark et al. 2004 |
| *Psylliodes cucullata*  (Illiger, 1807) | *Poa pratensis, Agropyron desertorum, A. cristatum, Festuca ovina, Eremopyrum cristatum, Spergula arvensis, Sisymbrium* spp., *Erysimum* spp., *Allium* spp. | Poaceae,  Caryophyllaceae, Brassicaceae  Amaryllidaceae | POL | Europe; Russia, Mongolia, China, Korea; Nearctic | PAR, NAR | Doguet 1994; Jolivet 1967; Nadein 2007b; 2010; Rheinheimer and Hassler 2018 |
| *Psylliodes cuprea*  (Koch, 1803) | *Sisymbrium officinale, Brassica nigra,*  *B. fruticulosa, Diplotaxis muralis, Isatis tinctoria, I. lusitanica, Alyssum* spp*., Raphanus raphanistrum, Descurainia sophia, Hirschfeldia incana, Sinapis alba, S. arvensis, Erucaria boveana, E. hispanica, Diplotaxis erucoides, Rapistrum rugosum, Raphanus rostratus, Ochthodium aegyptiaca, Erysimum* spp.*, Barbarea minor, Coincya wrightii, Fibigia macrocarpa, Lepidium* spp., *Aubrieta* spp. | Brassicaceae | OLI | Europe; Algeria, Morocco, Tunisia; Middle East | PAR | Baviera and Biondi 2015; Doguet 1994; Furth 1983; Jolivet 1967; Mohr 1966; Rheinheimer and Hassler 2018 |
| *Psylliodes cupreata*  (Duftschmid, 1825) | *Brassica rapa, B. napobrassica, Descurainia sophia, Camelina sativa, Berteroa incana, Erucastrum gallicum, Sinapis* spp., *Sisymbrium* spp., *Beta vulgaris* | Brassicaceae, Amaranthaceae | POL | Europe, Kyrgyzstan, Kazakhstan, Mongolia, Uzbekistan | PAR | Doguet 1994; Hajyieva and Soroka 2008; Kamiński 1936; Nadein 2010; Zverezomb-Zubovsky 1956; Rheinheimer and Hassler 2018 |
| *Psylliodes cyanescens*  Weise, 1887 | Unknown | Unknown | Unknown | Russia (East Siberia, far East) | PAR | Döberl 2010 |
| *Psylliodes danieli*  Weise, 1900 | Unknown | Unknown | Unknown | Italy, Austria, Croatia, Slovenia | PAR | Nadein 2007c; Weise 1900 |
| *Psylliodes deplanata*  Medvedev, 1962 | Unknown | Unknown | Unknown | Georgia, Russia (Southern) | PAR | Döberl 2010 |
| *Psylliodes dilutella*  Heikertinger, 1911 | *Solanum* spp. | Solanaceae | MON | Kyrgyzstan, Kazakhstan, Tajikistan, Uzbekistan | PAR | Döberl 2010; Lopatin 2010 |
| *Psylliodes dogueti*  Warchalowski, 1993 | Unknown | Unknown | Unknown | Turkey | PAR | Döberl 2010; Ekiz et al. 2013 |
| *Psylliodes drusei*  Furth, 1983 | *Quercus* spp. | Fagaceae | MON | Israel, Turkey | PAR | Aslan and Gök 2007; I. Şen (pers. comm.) |
| *Psylliodes dulcamarae*  (Koch, 1803) | *Solanum dulcamara, Atropa belladonna, Hyoscyamus niger* | Solanaceae | OLI | Europe; Kazakhstan, Mongolia, Turkey | PAR | Doguet 1994; Nadein 2010; Rheinheimer and Hassler 2018 |
| *Psylliodes elegans*  Horn, 1889 | *Brassica napus, Cakile lanceolata, Lepidium virginicum* | Brassicaceae | OLI | North America | NAR | Clark et al. 2004 |
| *Psylliodes erberi*  Döberl, 1995 | Unknown | Unknown | Unknown | Portugal (end. Madeira) | PAR | Döberl 2010 |
| *Psylliodes fageli*  Bechyne, 1957 | Unknown | Unknown | Unknown | Algeria | PAR | Döberl 2010 |
| *Psylliodes feroniae*  Leonardi, 1978 | *Quercus* spp. | Fagaceae | MON | Italy (Southern) | PAR | Baviera and Biondi 2015; Biondi and De Nardis 2001; M. Biondi pers. comm. |
| *Psylliodes festae*  Dodero, 1925 | *Hirschfeldia incana, Brassica tournefortii, B. nigra, Diplotaxis erucoides, Sinapis alba, Rapistrum rugosum, Raphanus aucheri, Erucaria boveana, Ochthodium aegyptiacum, Alyssum baumgartnerianum, Biscutella* spp., (also collected on *Quercus* spp. probably as refugial plant) | Brassicaceae | OLI | Mediterranean Region | PAR | Doguet and Leonardi 2017; Furth 1983 |
| *Psylliodes fiorellae*  Leonardi, 1978 | Unknown | Unknown | Unknown | Italy (Northern) | PAR | Döberl 2010 |
| *Psylliodes frivaldszkyi*  Weise, 1888 | Unknown | Unknown | Unknown | Romania, Ukraine, Slovakia, Poland, Austria, Czech republic | PAR | Döberl 2010; Nadein 2007c |
| *Psylliodes fusiformis*  (Illiger, 1807) | *Sinapis arvensis, S. pubescens, Brassica oleracea* | Brassicaceae | OLI | Cyprus, Italy, Portugal, Spain, Algeria, Morocco, Tunisia, Switzerland | PAR | Petitpierre 1999 |
| *Psylliodes gibbosa*  Allard, 1861 | *Lolium perenne, Desmazeria rigida, Scleropoa rigida* | Poaceae | OLI | Mediterranean Region | PAR | Doguet 1994; Nadein 2008; Petitpierre 1999 |
| *Psylliodes gigantea*  Chûjô, 1935 | Unknown | Unknown | Unknown | Taiwan | ORR | Chûjô 1935 |
| *Psylliodes glabra*  (Duftschmid, 1825) | Unknown (possibly *Biscutella laevigata*) | Unknown | Unknown | Austria, Croatia, Bosnia Herzegovina, Germany, Italy, Slovenia | PAR | Nadein 2007c; Rheinheimer and Hassler 2018; F. Fritzlar (pers. comm.) |
| *Psylliodes globosa*  Nadein, 2007 | Unknown | Unknown | Unknown | Nepal | ORR | Döberl 2010; Nadein 2007a |
| *Psylliodes gougeleti*  Allard, 1859 | Poaceae | Poaceae | Unknown | Portugal, Spain, Algeria, Morocco | PAR | Baselga and Novoa 2003b; Craven 2007; Nadein 2008 |
| *Psylliodes gracilis*  Boheman, 1859 | Unknown | Unknown | Unknown | Malaysia (Peninsular) | ORR | Mohamedsaid 2004 |
| *Psylliodes grigorievi*  Jacobson, 1922 | Unknown | Unknown | Unknown | Uzbekistan | PAR | Döberl 2010 |
| *Psylliodes guatemalensis*  Jacoby, 1885 | Unknown | Unknown | Unknown | Guatemala, Arizona, Mexico | NAR, NTR | Furth 2009 |
| *Psylliodes gyirongana*  Chen and Wang, 1981 | Unknown | Unknown | Unknown | China (Tibet) | PAR | Döberl 2010 |
| *Psylliodes heikertingeri*  Jakobson, 1922 | Brassicaceae | Brassicaceae | OLI | Italy (only in Sicily), Algeria, Tunisia | PAR | Baviera and Biondi 2015 |
| *Psylliodes hermonensis*  Furth, 1983 | *Erysimum verrucosum, E. goniocaulon, Hirschfeldia* spp*., Quercus* spp. | Brassicaceae, Fagaceae | POL | Israel | PAR | Furth 1983 |
| *Psylliodes heydeni*  Weise, 1888 | *Poa* spp*., Agropyron* spp., *Festuca* spp. | Poaceae | OLI | Spain, Portugal, France (Pyrenees) | PAR | Doguet 1994; Nadein 2007b; Ugarte 2005; M. Biondi (pers. comm.) |
| *Psylliodes hispana*  Heikertinger, 1911 | *Coincya* spp., *Brassica* spp*.* | Brassicaceae | OLI | France, Portugal, Spain | PAR | Baselga and Novoa 2001; Craven 2007; Doguet 1994 |
| *Psylliodes hospes*  Wollaston, 1854 | *Sinapis* spp., *Lobularia maritima, Crambe kralickii, Farsetia ovalis, F. aegyptiaca, Diplotaxis* spp., *Zilla spinosa, Erucastrum canariense* | Brassicaceae | OLI | France, Italy, Spain (incl. Canary Islands), Morocco, Egypt, Israel | PAR | Baviera and Biondi 2015; Doguet 1994; Biondi 1995; Craven 2007; Furth 1983; Heikertinger 1926; Petitpierre 1999 |
| *Psylliodes huaxiensis*  Wang, 1992 | Unknown | Unknown | Unknown | China (Yunnan, Sichuan) | ORR | Döberl 2010 |
| *Psylliodes hyoscyami*  (Linnaeus, 1758) | *Hyoscyamus* spp.*, Atropa belladonna, Solanum dulcamara, Datura* spp. | Solanaceae | OLI | Europe; Middle East; Algeria, Tunisia | PAR | Baviera and Biondi 2015; Furth 1983; Doguet 1994; Nadein 2010; Rheinheimer and Hassler 2018 |
| *Psylliodes illyrica*  Leonardi and Gruev*,* 1993 | *Quercus ilex* | Fagaceae | MON | Romania, Ukraine, Turkey, Serbia and Montenegro, Slovakia, Austria, Czech Republic | PAR | Nadein 2010 |
| *Psylliodes infanda*  Nadein, 2005 | Unknown | Unknown | Unknown | South West Mongolia | PAR | Nadein 2005 |
| *Psylliodes inflata*  Reiche and Saulcy 1858 | *Hirschfeldia incana, Brassica nigra, Barbarea minor, Erysimum verrucosum, Cladanthus mixtus, Anacyclus clavatus, Poa* spp. | Brassicaceae, Asteraceae,  Poaceae | POL | France, Italy (only in Sardinia and Sicily), Malta, Spain, Portugal, Algeria, Libya, Morocco, Tunisia; Middle East | PAR | Baviera and Biondi 2015; Furth 1983; Jolivet 1967 |
| *Psylliodes instabilis*  Foudras, 1860 | *Sinapis arvensis, S. alba, Erysimum* spp.*, Berteroa incana, Iberis* spp.*, Aurinia petrea, Matthiola lunata, Alyssum* spp.*, Fibigia macrocarpa, Diplotaxis* spp.*, Barbarea minor, Arabis* spp. | Brassicaceae | OLI | Europe; Algeria, Morocco, Tunisia; Cyprus, Israel, Turkey, Jordan, Lebanon, Syria | PAR | Baviera and Biondi 2015; Doguet 1994; Furth 1983; Jolivet 1967; Nadein 2010; Rheinheimer and Hassler 2018 |
| *Psylliodes isatidis*  Heikertinger, 1913 | *Isatis* spp. (also reported on *Lepidium campestre, Brassica nigra, Sisymbrium officinale, Alyssum* spp.*, Hirschfeldia incana, Sinapis alba, S. arvensis, Erucaria boveana, E. hispanica, Diplotaxis erucoides, Rapistrum rugosum, Raphanus rostratus, Ochthodium aegyptiaca, Erysimum verrucosum,*  *E. goniocaulon, Fibigia macrocarpa, Barbarea* spp.) | Brassicaceae | MON | Europe; Iran, Uzbekistan, Kazakhstan, Turkey, Mongolia, Russia | PAR | Doguet 1994; Baviera and Biondi 2015; Nadein 2010; Rheinheimer and Hassler 2018 |
| *Psylliodes kasnakensis*  Gӧk and Aslan, 2007 | *Quercus* spp. | Fagaceae | MON | Turkey | PAR | Ekiz et al. 2013; Gök and Aslan 2007; Nadein 2006 |
| *Psylliodes kiesenwetteri*  Kutschera, 1864 | *Biscutella laevigata, Erysimum pseudorhaeticum,* (probably also on Poaceae) | Brassicaceae | Unknown | Austria, Croatia, Albania, Bulgaria, Bosnia Herzegovina, Italy, Greece, Hungary, Slovakia, Macedonia, Serbia and Montenegro, Turkey, France (Corse) | PAR | Nadein 2008; M. Biondi (pers. comm.); F. Fritzlar (pers. comm.) |
| *Psylliodes kikuyuana*  Biondi, 1996 | Unknown | Unknown | Unknown | Kenya | AFR | Biondi 1996; Nadein 2007a |
| *Psylliodes konstantinovi*  Lopatin, 1997 | Unknown | Unknown | Unknown | Kyrgyzstan | PAR | Döberl 2010 |
| *Psylliodes laevicollis*  (Dufour, 1851) | *Diplotaxis brassicoides, Rhynchosinapis cheiranthos* | Brassicaceae | OLI | France, Portugal, Spain, Morocco | PAR | Doguet 1994; Petitpierre 1999 |
| *Psylliodes laevifrons laevifrons*  Kutschera, 1864 | *Quercus ilex, Ostya carpinifoli, Corylus* spp.*, Ulmus* spp., *Fraxinus* spp. | Fagaceae, Betulaceae,  Ulmaceae,  Oleaceae | POL | France, Italy, Greece | PAR | Baviera and Biondi 2015; Doguet 1994; Gruev 1990; Leonardi 2013 |
| *Psylliodes laevifrons cretica*  Weise, 1888 | *Quercus ilex* | Fagaceae | Unknown | Greece (Crete) | PAR | Gruev 1990; Leonardi 2013 |
| *Psylliodes laticollis*  Kutschera, 1864 | *Nasturtium officinale, Alliaria petiolata* | Brassicaceae | OLI | Europe; Afrotropical Region; Algeria, Morocco, Tunisia, | PAR, AFR | Doguet 1994; Cox 2007; Nadein 2010; Rheinheimer and Hassler 2018 |
| *Psylliodes laurisilvae*  Biondi, 1987 | *Crambe* spp. | Brassicaceae | MON | Spain (Canary Islands – Tenerife) | PAR | Biondi 1987; 1995 |
| *Psylliodes laxa*  Nadein, 2006 | Unknown | Unknown | Unknown | Russia (far East) | PAR | Döberl 2010; Nadein 2006 |
| *Psylliodes leonhardi*  Heikertinger, 1926 | *Quercus* spp. (probably also Poaceae) | Fagaceae | MON | Italy | PAR | Baviera and Biondi 2015; Leonardi 2007 |
| *Psylliodes lethierryi*  Allard, 1860 | *Biscutella didyma, B. lyrata* | Brassicaceae | MON | Tunisia, Algeria, Italy (Sicily) | PAR | Doguet and Leonardi 2017 |
| *Psylliodes libanicola*  Pic, 1903 | Unknown | Unknown | Unknown | Lebanon | PAR | Döberl 2010 |
| *Psylliodes libertii*  Leonardi, 2013 | *Quercus* spp*., Ostrya carpinifolia* | Fagaceae, Betulaceae | POL | Italy | PAR | Baviera and Biondi 2015 |
| *Psylliodes littoralis*  Biondi, 1997 | Brassicaceae | Brassicaceae | Unknown | Cyprus, Turkey | PAR | Biondi 1997; Döberl 2010 |
| *Psylliodes longicollis*  Weise, 1900 | Unknown | Unknown | Unknown | Azerbaijan, Armenia, Georgia, Russia (Southern) | PAR | Döberl 2010 |
| *Psylliodes loriae*  Jacoby, 1904 | Unknown | Unknown | Unknown | Papua New Guinea | AUR | Jacoby 1904 |
| *Psylliodes luridipennis*  Kutschera, 1864 | *Coincya wrightii* | Brassicaceae | MON | Great Britain (Lundy Island) | PAR | Cox 1998; Cox 2007; Craven 2007 |
| *Psylliodes luteola*  O. F. Müller, 1776 | *Triticum aestivum, Hordeum* spp.*, Lolium* spp.*, Quercus robur, Q. petraea, Ulmus campestris, U. carpinifolia, Carpinus betulus, Populus nigra, P. alba, Salix alba, Solanum spp.* | Poaceae,  Fagaceae,  Ulmaceae,  Salicaceae,  Solanaceae | POL | Europe; Algeria, Morocco, Cyprus, Iran, Lebanon, Syria, Turkey | PAR | Baviera and Biondi 2015; Doguet 1994; Petitpierre 1999;  Rheinheimer and Hassler 2018 |
| *Psylliodes maculatipes*  Pic, 1924 | *Zilla spinosa* | Brassicaceae | MON | Algeria, Oman, Egypt (Sinai), Saudi Arabia | PAR | Döberl 2010; Furth 1983; Leonardi 2007 |
| *Psylliodes magnifica*  Gruev, 1975 | Unknown | Unknown | Unknown | Bulgaria (Mt. Strandzha), Greece (Northern) | PAR | Döberl 2010; Ekiz et al. 2013 |
| *Psylliodes manobioides*  Nadein, 2007 | *Arundinaria alpina* | Poaceae | MON | Kenya | AFR | Nadein 2007a |
| *Psylliodes marcida*  (Illiger, 1807) | *Cakile maritima, Cochlearia* spp., *Lobularia maritima, Crambe maritima, Coincya monensis, Raphanus* spp. | Brassicaceae | OLI | Europe; Algeria, Morocco, Tunisia, Israel, Lebanon, Turkey | PAR | Craven 2007; Furth 1983; Jolivet 1967; Rheinheimer and Hassler 2018; Doguet 1994 |
| *Psylliodes marcosellai*  Biondi, 1996 | Unknown | Unknown | Unknown | Tanzania | AFR | Biondi 1996 |
| *Psylliodes maroccana*  Heikertinger, 1916 | *Cakile maritima, Lobularia maritima, Matthiola sinuata* | Brassicaceae | OLI | France, Italy, Spain (incl. Canary Islands), Morocco | PAR | Baviera and Biondi 2015 |
| *Psylliodes masai*  Biondi, 1996 | Unknown | Unknown | Unknown | Kenya | AFR | Biondi 1996 |
| *Psylliodes melanocephala*  Jacoby, 1891 | Unknown | Unknown | Unknown | Mexico (Districto Federal) | NTR | Furth 2006 |
| *Psylliodes metatarsalis*  Leonardi, 2007 | Unknown | Unknown | Unknown | Algeria | PAR | Döberl 2010 |
| *Psylliodes mexicana*  Jacoby, 1891 | Unknown | Unknown | Unknown | Mexico (Districto Federal) | NTR | Furth 2006 |
| *Psylliodes milleri lindbergi*  Madar and Madar, 1964 | Unknown | Unknown | Unknown | Spain, Algeria, Morocco | PAR | Döberl 2010 |
| *Psylliodes milleri milleri*  Kutschera, 1864 | *Erysimum* spp., *Biscutella* spp. | Brassicaceae | OLI | Greece, Italy, Spain, Cyprus, Turkey | PAR | Baviera and Biondi 2015; Döberl 2010; F. Fritzlar (pers. comm.) |
| *Psylliodes montana*  Wiese, 1910 | Unknown | Unknown | Unknown | Tanzania | AFR | Biondi 1996; Nadein 2007a |
| *Psylliodes moricandiae*  Peyerimhoff, 1925 | *Diplatoxis acris, D. harra, Erucaria boveana, Matthiola longipetala* | Brassicaceae | OLI | Algeria, Tunisia | PAR | Furth 1983; Leonardi 2007 |
| *Psylliodes napi*  Fabricius, 1792 | *Alliaria petiolata, Brassica* spp., *Nasturtium officinale, Cardamine impatiens, C. amara, Barbarea vulgaris, Crambe maritima, Lunaria rediviva, Rorippa* spp.*, Coincya wrightii, Cochlearia danica, Erucastrum* spp*.* | Brassicaceae | OLI | Europe; Nearctic; Algeria, Morocco, Russia, Kazakhstan, Turkey | PAR, NAR | Baviera and Biondi 2015; Doguet 1994; Nadein 2010; Rheinheimer and Hassler 2018 |
| *Psylliodes nigripennis*  Allard, 1860 | Unknown | Unknown | Unknown | Algeria, Tunisia | PAR | Döberl 2010 |
| *Psylliodes nigripes*  Boheman, 1853 | Unknown | Unknown | Unknown | Sweden | PAR | Döberl 2010 |
| *Psylliodes nigroaenea*  Jacoby, 1896 | Unknown | Unknown | Unknown | Indonesia (Sumatra) | ORR | Jacoby 1896 |
| *Psylliodes nitida*  Medvedev, 1973 | Unknown | Unknown | Unknown | Russia (far East) | PAR | Döberl 2010 |
| *Psylliodes nyalamana*  Chen and Wang, 1981 | Unknown | Unknown | Unknown | China | PAR | Döberl 2010 |
| *Psylliodes obscuroaenea*  Rosenhauer, 1856 | *Quercus ilex, Q. pyrenaica* | Fagaceae | MON | Italy, Spain, Portugal, Algeria, Morocco, Tunisia | PAR | Baviera and Biondi 2015; Baselga and Novoa 2003a; Döberl 2010 |
| *Psylliodes obscurofasciata*  Chen, 1933 | Unknown | Unknown | Unknown | China | PAR | Döberl 2010 |
| *Psylliodes olgae*  Nadein, 2007 | Unknown | Unknown | Unknown | Spain | PAR | Döberl 2010; Nadein 2007b |
| *Psylliodes ozisiki*  Leonardi and Arnold, 1995 | Unknown | Unknown | Unknown | Armenia, Turkey | PAR | Döberl 2010; Leonardi and Arnold 1995 |
| *Psylliodes pallidicolor*  Pic, 1903 | Unknown (possibly on *Quercus* spp*.*) | Unknown | Unknown | Bulgaria, Greece, Spain, Algeria, Israel, Lebanon, Syria, Turkey, Iraq | PAR | Furth 1983 |
| *Psylliodes pallidicornis*  Heikertinger 1921 | *Sisymbrium elatum* | Brassicaceae | MON | Armenia, Russia (Southern), Turkmenistan | PAR | Nadein 2010 |
| *Psylliodes pallidipennis*  Rosenhauer, 1856 | *Matthiola incana, Brassica radicata, Cakile maritima, Reseda alba* | Brassicaceae, Resedaceae | OLI | France, Italy, Malta, Portugal, Spain, Algeria, Morocco, Tunisia, Lebanon, Syria | PAR | Baviera and Biondi 2015; Doguet 1994; Heikertinger 1926; Petitpierre 1999 |
| *Psylliodes parilis*  Weise, 1923 | *Duboisia leichhardtii, D. myoporoides* | Solanaceae | MON | Australia | AUR | Razzaque 2002 |
| *Psylliodes parodii*  Leonardi, 2007 | *Sesleria* spp. | Poaceae | MON | Italy | PAR | Döberl 2010; Leonardi 2007 |
| *Psylliodes persica*  Allard, 1867 | *Capsella bursa-pastoris, Brassica napus* | Brassicaceae | OLI | Turkey, Afghanistan, Iran, Iraq, Jordan, Kazakhstan, Saudi Arabia, Syria, Uzbekistan, Turkmenistan, Tajikistan | PAR | Aslan and Ghahari 2017; Nadein 2010 |
| *Psylliodes petasata*  Foudras, 1860 | *Cerastium* spp. | Caryophyllaceae | MON | Spain, France (Pyrenees) | PAR | Doguet 1994 |
| *Psylliodes peyerimhoffi*  Heikertinger, 1916 | Unknown | Unknown | Unknown | Egypt, Arab Emirates, Saudi Arabia, Israel | PAR | Döberl 2010 |
| *Psylliodes picina*  (Marsham, 1802) | *Phalaris* spp., *Phragmites australis, Quercus* spp., *Ulmus* spp., *Corylus* spp.*, Betula* spp.*, Eupatorium cannabinum, Cirsium palustre, C. arvense, Lythrum salicaria, Lysimachia vulgaris* | Poaceae,  Fagaceae,  Ulmaceae,  Betulaceae,  Asteraceae,  Lythraceae,  Primulaceae | POL | Europe, Nearctic | PAR, NAR | Doguet 1994;  Gruev and Döberl 2005; Heikertinger 1926; Nadein 2010; Rheinheimer and Hassler 2018 |
| *Psylliodes picipes*  Redtenbacher, 1849 | *Lunaria rediviva, Biscutella laevigata* | Brassicaceae | OLI | Austria, Croatia, France, Italy, Slovakia | PAR | Brelih et al. 2003; Doguet 1994; Nadein 2010 |
| *Psylliodes plana*  Maulik, 1926 | Unknown | Unknown | Unknown | China, India; Oriental Region | PAR, ORR | Döberl 2010 |
| *Psylliodes puncticollis*  Rosenhauer, 1856 | *Calamagrostis* spp*., Stipa offneri, Apocynum venetum, Oenothera biennis, Ammophila arenaria, Centaurea* spp*., Quercus faginea, Q. rotundifolia* | Poaceae, Apocynaceae, Onagraceae, Asteraceae, Fagaceae | POL | Albania, Bulgaria, Croatia, France, Greece, Italy, Spain, Algeria, Tunisia | PAR | Baviera and Biondi 2015; Brelih et al. 2003; Doguet 1994; Heikertinger 1926; Petitpierre 1999 |
| *Psylliodes punctifrons*  Baly, 1874 | *Cardamine anemonoides, Brassica chinensis, B. napus* | Brassicaceae | OLI | Russia (far East); Oriental Region | PAR, ORR | Takizawa 2005 |
| *Psylliodes punctulata*  Melsheimer, 1847 | *Humulus lupulus, Cannabis sativa, Barbarea vulgaris, Brassica* spp.*, Cardamine diphylla, Descurainia sophia, Erysimum cheiranthoides, E. repandum, Lepidium* spp*.,* *Norta altissima, Raphanus sativus, Nasturtium officinale, Sisymbrium officinale, Beta vulgaris, Amaranthus* spp.*, Solanum tuberosum* | Cannabaceae, Brassicaceae, Amaranthaceae, Solanaceae | POL | North America (Colorado), Guatemala, Mexico | NAR, NTR | Clark et al. 2004 |
| *Psylliodes pyrenaea*  Heikertinger, 1912 | Unknown | Unknown | Unknown | France (Pyrenees, Basses-Alps), Spain (Avila) | PAR | Petitpierre 1999 |
| *Psylliodes pyritosa*  Kutschera, 1864 | *Brassica* spp.*, Lepidium draba, Sinapis arvensis, Sisymbrium officinale, Rorippa lippicensis, Alyssoides sinuata* | Brassicaceae | OLI | Portugal (incl. Madeira), Spain (incl. Canary Islands), Italy, France, Greece, Austria, Serbia and Montenegro, Bosnia and Herzegovina; Afrotropical Region | PAR, AFR | Baviera and Biondi 2015; Doguet 1994; Nadein 2010 |
| *Psylliodes quadridentata*  Baly, 1876 | Unknown | Unknown | Unknown | Australia | AUR | Döberl 2010 |
| *Psylliodes rhaica*  Jacobson, 1922 | *Solanum* spp. | Solanaceae | MON | Russia, Ukraine, Kazakhstan | PAR | Döberl 2010; Olegovich 2011 |
| *Psylliodes ridenda*  Nadein, 2008 | Unknown | Unknown | Unknown | Turkey | PAR | Döberl 2010; Nadein 2008 |
| *Psylliodes rubroaenea*  Heikertinger, 1916 | Unknown | Unknown | Unknown | Georgia, Russia (Southern) | PAR | Nadein 2007c |
| *Psylliodes ruffoi*  Leonardi, 1975 | Poaceae | Poaceae | Unknown | Italy | PAR | Baviera and Biondi 2015 |
| *Psylliodes ruficolor*  Doguet, 1992 | *Quercus* spp. | Fagaceae | MON | France, Spain, Algeria, Tunisia | PAR | Doguet 1994; Vela et al. 2017 |
| *Psylliodes rufitarsis*  Graells, 1858 | Unknown | Unknown | Unknown | Spain | PAR | Döberl 2010 |
| *Psylliodes saulcyi*  Allard, 1867 | *Atriplex lacinata, A. halimus, Chenopodium album, Beta vulgaris* | Amaranthaceae | OLI | Iraq, Cyprus, Syria, Jordan, Israel, Ukraine, Egypt, Turkey, Arab Emirates, Kazakhstan, Mongolia, Iran | PAR | Furth 1983; Heikertinger 1926 |
| *Psylliodes schwarzi*  Weise, 1900 | Unknown | Unknown | Unknown | France, Italy, Switzerland | PAR | Doguet 1994; Döberl 2010 |
| *Psylliodes scutellata*  (Waterhouse, 1838) | *Zea mays, Solanum* spp.*, Duboisia* spp*., Datura* spp*., Hibiscus cannabinum* | Poaceae, Solanaceae, Malvaceae | POL | Australia | AUR | Hawkeswood and Furth 1994 |
| *Psylliodes semicnemis*  Gruev, 1990 | Unknown | Unknown | Unknown | Nepal | ORR | Döberl 2010 |
| *Psylliodes shira*  Maulik, 1926 | Unknown | Unknown | Unknown | Nepal, Oriental Region | ORR | Döberl 2010 |
| *Psylliodes shirensis*  Biondi and D'Alessandro, 2018, 2019 | Unknown | Unknown | Unknown | Tanzania | AFR | Biondi and D'Alessandro 2018, 2019 |
| *Psylliodes solarii*  Leonardi, 1975 | Unknown | Unknown | Unknown | France, Italy | PAR | Doguet 1994; Döberl 2010 |
| *Psylliodes springeri*  Leonardi, 1975 | *Isatis apennina* | Brassicaceae | MON | Italy | PAR | Leonardi 2007 |
| *Psylliodes sturanyi*  Apfelbeck, 1906 | Poaceae | Poaceae | Unknown | Bulgaria, Serbia and Montenegro, Bosnia Herzegovina, Romania, Austria | PAR | Apfelbeck 1906; Nadein 2007c |
| *Psylliodes subaenea styriaca* Heikertinger, 1921 | *Hornungia alpin, Thlaspi* spp. | Brassicaceae | OLI | Austria, Slovakia | PAR | Brelih et al. 2003 |
| *Psylliodes subaenea subaenea* Kutschera, 1864 | Unknown (possibly Brassicaceae, considering the host plant family of subspecies *Ps. subaenea styriaca* ) | Unknown | Unknown | Croatia, Bosnia Herzegovina, Poland, Romania, Slovakia, Ukraine, Serbia and Montenegro | PAR | Döberl 2010; M. Biondi (pers. comm.) |
| *Psylliodes sublaevis*  Horn, 1889 | Unknown | Unknown | Unknown | North America (North Carolina), Mexico (Guerrero) | NAR, NTR | Clark et al. 2004 |
| *Psylliodes submontana*  Nadein, 2006 | Unknown | Unknown | Unknown | Russia | PAR | Nadein 2006 |
| *Psylliodes subrugosa*  Jacoby, 1885 | *Rorippa indica, R. islandica, Cardamine* spp*.* | Brassicaceae | OLI | Russia, Japan, China | PAR | Nadein and Lee 2012; Takizawa 2005; Yano and Ohsaki 1993 |
| *Psylliodes sumatrensis*  Jacoby, 1893 | Unknown | Unknown | Unknown | Indonesia (Sumatra) | ORR | Jacoby 1893 |
| *Psylliodes taiwana*  Takizawa, 1979 | Unknown | Unknown | Unknown | Japan (Ryukyu), Taiwan | PAR,ORR | Döberl 2010 |
| *Psylliodes taiwanica*  Chûjô, 1935 | Unknown | Unknown | Unknown | Taiwan | ORR | Döberl 2010 |
| *Psylliodes takizawai*  Gruev, 1990 | Unknown | Unknown | Unknown | North Korea | PAR | Döberl 2010 |
| *Psylliodes taurica*  Leonardi, 1971 | Unknown | Unknown | Unknown | Turkey | PAR | Döberl 2010 |
| *Psylliodes tenebrosa*  Jacoby, 1896 | *Erysimum* spp. | Brassicaceae | MON | Nepal, Pakistan, India; Oriental Region | ORR | Scherer 1969 |
| *Psylliodes tenuidentata*  Nadein, 2008 | Unknown | Unknown | Unknown | Israel | PAR | Döberl 2010; Nadein 2008 |
| *Psylliodes teresae*  Biondi, 1996 | *Lycium* cfr *ferocissimus* | Solanaceae | MON | South Africa | AFR | Biondi 1996 |
| *Psylliodes testaceoconcolor*  Heikertinger, 1962 | *Crambe hispanica, Hirschfeldia incana* | Brassicaceae | OLI | Syria, Israel, Lebanon, Turkey, Azerbaijan | PAR | Furth 1983 |
| *Psylliodes thlaspis*  Foudras, 1860 | *Lepidium* spp.*, Iberis umbellata, I. amara, Thlaspi arvensis, Arabis glabra, Isatis tinctoria* | Brassicaceae | OLI | Europe; Turkey | PAR | Brelih et al. 2003; Doguet 1994; Rheinheimer and Hassler 2018 |
| *Psylliodes tibetana*  Chen, 1976 | Unknown | Unknown | Unknown | Tibet (Xizang) | PAR | Döberl 2010 |
| *Psylliodes toelgi*  Heikertinger, 1914 | *Biscutella laevigata* | Brassicaceae | MON | Italy, Austria, Croatia, Slovenia, Russia, France, Germany, Hungary, Czech Republic, Serbia and Montenegro, Slovenia, Ukraine, Romania, Turkey | PAR | Brelih et al. 2003; Doguet 1994; Fritzlar 2009; Rozner and Rozner 2014; Rheinheimer and Hassler 2018 |
| *Psylliodes tricolor*  Weise, 1888 | *Descurainia sophia, Sisymbrium officinale*,  *S. sophia, Isatis tinctoria, Barbarea minor, Erysimum verrucosum, Alyssum baumgartenerianum* | Brassicaceae | OLI | Europe; Morocco, Afghanistan, Russia, Israel, Iran, Kazakhstan, Uzbekistan | PAR | Furth 1983; Doguet 1994; Nadein 2010; Rheinheimer and Hassler 2018 |
| *Psylliodes tristis*  Jacoby, 1885 | Unknown | Unknown | Unknown | Guatemala | NTR | Jacoby 1885 |
| *Psylliodes tsinghaina*  Chen and Zia, 1966 | Unknown | Unknown | Unknown | Qinghai (Tsinghai) | PAR | Döberl 2010 |
| *Psylliodes umbratilis*  Wollaston, 1854 | Poaceae | Poaceae | Unknown | Portugal (end. Madeira) | PAR | Wollaston 1854 |
| *Psylliodes urbaniae*  Biondi and D’Alessandro 2017 | *Lunaria annua* | Brassicaceae | MON | Italy (Central Apennines) | PAR | Biondi and D'Alessandro 2017 |
| *Psylliodes valida*  Weise, 1889 | *Cardamine bulbifera, Alliaria petiolata* | Brassicaceae | OLI | Russia, Georgia, Turkey | PAR | Nadein 2010 |
| *Psylliodes vehemens normandi*  Heikertinger, 1916 | Brassicaceae (Possibly also on *Tipuana tipu* and Poaceae) | Brassicaceae | Unknown | Portugal (incl. Azores, Madeira), Spain (end. Malaga, Canary Islands), Algeria, Morocco, Tunisia | PAR | Biondi 1995; Döberl 2010; D. Teixidor (pers. comm.) |
| *Psylliodes vehemens vehemens*  Wollaston, 1854 | Brassicaceae | Brassicaceae | OLI | Portugal (end. Madeira) | PAR | Biondi 1995 |
| *Psylliodes verisimilis*  Fall, 1933 | Unknown | Unknown | Unknown | North America | NAR | Clark et al. 2004 |
| *Psylliodes vindobonensis*  Heikertinger, 1914 | *Erysimum virgatum, E. marschallianum, Arabis* spp. | Brassicaceae | OLI | Austria, Czech Republic, France, Germany, Italy, Spain, Switzerland, Turkey, Ukraine, Croatia | PAR | Doguet 1994; Nadein 2010; Rheinheimer and Hassler 2018 |
| *Psylliodes viridana*  Motschulsky, 1858 | *Solanum* spp.*, Nicotiana tabaccum* | Solanaceae | OLI | Russia, Taiwan, Nepal; Oriental Region | PAR, ORR | Nadein and Lee 2012; Takizawa 2005 |
| *Psylliodes wachsmanni*  Csiki, 1903 | *Quercus infectoria* | Fagaceae | MON | Italy (Northern), Albania, Croatia, Bosnia Herzegovina, Slovakia, Turkey, Serbia and Montenegro, Cyprus, Jordan | PAR | Biondi 1994; Leonardi 1972 |
| *Psylliodes wrasei*  Leonardi and Arnold, 1995 | *Lepidium draba* | Brassicaceae | MON | Bulgaria, Greece, Georgia, Macedonia, Ukraine, Turkey | PAR | Aslan et al. 2014; Nadein 2010 |
| *Psylliodes wunderlei*  Döberl, 1998 | Unknown | Unknown | Unknown | Spain (Santander) | PAR | Döberl 2010 |
| *Psylliodes yalvacensis*  Gök, 2005 | Unknown (possibly on *Quercus* spp.) | Unknown | Unknown | Turkey (South West) | PAR | Gök 2005; I. Şen (pers. comm.) |
| *Psylliodes yuae*  Nadein, 2012 | *Lycianthes lysimachioides* | Solanaceae | MON | Taiwan | ORR | Nadein and Lee 2012 |

^1^ All recorded food plants were included (abbreviated spp. when more than two species in the same genus were reported), with refugial or uncommon food plants in parenthesis.

^2^ Only the well documented food plant families were considered.

^3^ Only species with well documented information regarding their food plants had their diet breadth specified.

^4^ Abbreviations of locations: end., endemic; incl., including.

^5^ The references are in regards to the food plants information. For the species where food plant information is unknown, either the catalogue by Döberl (2010) was used as the secondary source of information or the original paper where the species was described.

**REFERENCES**

Apfelbeck V (1906) Nove vrste koleoptera s Balkanskog poluostrva. . Glasnik Zemaljskog Muzeja u Bosni i Hercegovini 17: 239-251

Aslan B, Bayram F, Aslan EG (2014) First record of the flea beetle *Psylliodes wrasei* Leonardi and Arnold (Chrysomelidae: Galerucinae: Alticini) in Turkey: a promising biological control agent for Hoary Cress, *Lepidium draba* L . (Brassicaceae). Journal of the Entomological Research Society 16: 111-115

Aslan EG, Ghahari H (2017) An annotated synopsis of the flea beetles of Iran with new records (Coleoptera: Chrysomelidae: Galerucinae: Alticini). Transactions of the American Entomological Society 143: 633-667

Aslan EG, Gök A (2006) Host-plant relationships of 65 flea beetles species from Turkey, with new associations (Coleoptera: Chrysomelidae: Alticinae). Entomological News 117: 297-308. doi:10.3157/0013-872X(2006)117[297:HROFBS]2.0.CO;2

Aslan EG, Gök A (2007) Two new records of leaf beetles from Turkey with habitat and host plant information (Coleoptera: Chrysomelidae). Entomologische Zeitschrift 117: 103-104

Baly JS (1876) Descriptions of new genus and of new species of Halticinae. Transactions of the Entomological Society of London: 581-602

Baselga A, Novoa F (2001) Citas nuevas de Chrysomelidae (Coleoptera) de Galicia (Noroeste de la Península Ibérica ). Boletin de la Asociación Española de Entomologia 25: 125-131

Baselga A, Novoa F (2003a) Los Chrysomelidae de los Arribes del Duero, noroeste de la Península Ibérica (Coleoptera). Nouvelle Revue d'Entomologie, Nouvelle Série 20: 117-131

Baselga A, Novoa F (2003b) A new species of *Psylliodes* (Coleoptera: Chrysomelidae) and key to the wingless species from the Iberian Peninsula. Annals of the Entomological Society of America 96: 689-692. doi:10.1603/0013-8746(2003)096[0689:ANSOPC]2.0.CO;2

Baviera C, Biondi M (2015) The Alticini (Coleoptera: Chrysomelidae, Galerucinae) of Sicily: Recent records and updated checklist. Atti della Accademia Peloritana dei Pericolanti, Classe di Scienze Fisiche, Matematiche e Naturali 93: A2-A50. doi:10.1478/AAPP.932A2

Biondi M (1987) Contributo alla conoscenza dei Chrysomelidae Alticinae delle isole Canarie , con descrizione di una nuova specie di *Psylliodes* (Coleoptera). Vieraea 17: 93-97

Biondi M (1994) Contribution a l'histoire naturelle de l'île de Chypre. Coleoptera: Chrysomelidae Alticinae. Biocosme Mésogéen Nice 11:19–25

Biondi M (1995) Gli Alticini Delle Isole Canarie. Fragmenta Entomologica 26: 1-133

Biondi M (1996) The genus *Psylliodes* in the Afrotropical region with description of five new species from Kenya, Tanzania and South Africa. Fragmenta Entomologica 28: 257-276

Biondi M, D'Alessandro P (2017) *Psylliodes urbaniae*: a new species from Central Apennines (Coleoptera: Chrysomelidae: Galerucinae: Alticini). Onychium 13: 121-130. doi:10.5281/zenodo.495568

Biondi M, D'Alessandro P (2018) Two new species of the flea beetle genus *Psylliodes* Latreille of the *montana* species-group from Eastern Africa (Coleoptera: Chrysomelidae). Fragmenta Entomologica 50: 87-93. doi:doi.org/10.4081/fe.2018.305

Biondi M, D’Alessandro P (2019) *Psylliodes shirensis*, a new replacement name for *Psylliodes shira* Biondi & D’Alessandro (Coleoptera: Chrysomelidae). Fragmenta Entomologica 51 (in press).

Biondi M, De Nardis G (2001) I Coleoptera Chrysomelidae del Massiccio del Gran Sasso d'Italia: proposta per uno studio ecologico finalizzato al monitoraggio ambientale. In: Cicolani B (Ed) Monitoraggio Biologico del Gran Sasso. Andromeda Editrice, 36-54.

Brelih S, Döberl M, Drovenik B, Pirnat A (2003) Materialien zur Käferfauna (Coleoptera) Slowenien. 1. Beitrag: Polyphaga: Chrysomeloidea (= Phytophaga): Chrysomelidae: Alticinae. Scopolia 50: 1-279

Chûjô M (1935) Studies on the Chrysomelidae in the Japanese Empire VIII. Transactions of the Natural History Society of Formosa 25: 354-369

Clark SM, LeDoux DG, Seeno TN, Riley EG, Gilbert AJ, Sullivan JM (2004) Host plants of leaf beetle species occurring in the United States and Canada (Coleoptera: Orsodacnidae, Megalopodidae, Chrysomelidae exclusive of Bruchinae). Coleopterist Society, Special Publication no. 2, 476 pp.

Cox ML (1998) The genus *Psylliodes* Latreille (Chrysomelidae: Alticinae) in the U.K.: with keys to the adults of all species and to the larvae of those species feeding on Brassicaceae. The Coleopterist 7: 33-65

Cox ML (2007) Atlas of the seed and leaf Beetles of Britain and Ireland. Pisces Publications, Newbury, Berkshire, 334 pp.

Craven JC (2007) The Evolution and Conservation Ecology of the Lundy Cabbage and its Beetles. PhD Thesis, Leeds, United Kingdom: The University of Leeds.

Döberl M (2010) Subfamily Alticinae. In: Löbl I, Smetana A (Eds) Catalogue of Palearctic Coleoptera. Apollo Books, Stenstrup, Denmark, 491-563

Doguet S (1994) Coléoptère Chrysomelidae. Volume 2. Alticinae. Faune de France, France et Régions Limitrophes 80. Federation Francaise des Societes de Sciences naturelles, Paris, 694 pp.

Doguet S, Leonardi C (2017) Critical study on *Psylliodes circumdata* (W. Redtenbacher) with restoration of two species (Coleoptera: Chrysomelidae). Entomologische Blätter und Coleoptera 113: 55-66

Ekiz AN, Şen Iİ, Aslan EG, Gök A (2013) Checklist of leaf beetles (Coleoptera: Chrysomelidae) of Turkey, excluding Bruchinae. Journal of Natural History 47: 2213-2287. doi:10.1080/00222933.2012.763069

Fritzlar F (2009) Neue und interessante Blattkäfer-Nachweise aus Thüringen und anderen Bundesländern (Coleoptera, Chrysomelidae), Teil 5. Thüringer Faunistische Abhandlungen XIV: 181-210

Furth DG (1983) Alticinae of Israel: *Psylliodes* (Coleoptera: Chrysomelidae). Israel Journal of Entomology 17: 37-58

Furth DG (2006) The current status of knowledge of the Alticinae of Mexico (Coleoptera: Chrysomelidae). Bonner zoologische Beiträge 54: 209-237

Furth DG (2009) Flea beetle diversity of the Sierra Tarahumara, Copper Canyon, Mexico (Chrysomelidae: Alticinae). Research on Chrysomelidae 2: 131-151. doi:10.1163/ej.9789004169470.1-299.45

Gök A (2005) *Psylliodes yalvacensis* sp. n. (Coleoptera, Chrysomelidae, Alticinae) from Turkey. Biologia Bratislava 60: 133-135. doi:10.1080/13880200590903354

Gök A, Aslan EG (2007) A new species of *Psylliodes* Latreille (Coleoptera: Chrysomelidae) from Turkey. Entomological News 118: 371-376

Gök A, Çilbiroğlu EG (2004) A new species of the genus *Psylliodes* Latreille (Coleoptera: Chrysomelidae) from Turkey. Zootaxa 440: 1-6

Gök A, Doguet S, Cilbiroglu EG (2003) *Psylliodes cerenae* sp. nov., a new Alticinae species from Southwest Turkey (Coleoptera: Chrysomelidae). Annales Zoologici 53: 201-202

Gruev B (1981) New data about some Chrysomelid beetles from China with a Description of a New Species of *Psylliodes* Latreille (Col., Chrysomelidae). The Entomological Review of Japan 35: 71-76

Gruev B (1990) The geographic distribution of Lamprosomatinae, Eumolpinae, Chrysomelinae, Alticinae, Hispinae and Cassidinae in Greece (Coleoptera, Chrysomelidae). Deutsche Entomologische Zeitschrift 37: 289-359. doi:10.1002/mmnd.19900370410

Gruev B, Döberl M (2005) General Distribution of the Flea Beetles in the Palaearctic Subregion (Coleoptera, Chrysomelidae: Alticinae) Supplement. Pensoft Publishers, Sofia, Moscow, 241-241 pp.

Hajyieva H, Soroka S (2008) Phytosanitary situation in sugar beet crops in Belarus. Zemdirbyste-Agriculture 95: 65-73

Hawkeswood TJ, Furth DG (1994) New host plant records for some Australian Alticinae. Spixiana 17: 43-49

Heikertinger F (1926) Bestimmungstabelle der Halticinengattung *Psylliodes* aus dem paläarktischen Gebiete. Koleopterologische Rundschau 12: 101-138

Jacoby M (1884) Descriptions of new genera and species of Phytophagous Coleoptera from Sumatra. Notes from the Leyden Museum 6: 9-70

Jacoby M (1885) Insecta, Coleoptera, Galerucidae. Halticinae. Phytophaga. Biologia Centrali-Americana 6: 263-625

Jacoby M (1893) Descriptions of some new genera and new species of Halticidae. Transactions of the Entomological Society of London: 145-158

Jacoby M (1896) Descriptions of the new genera and species of Phytophagous Coleoptera obtained by Dr. Modigliani in Sumatra. Annali del Museo Civico di Storia Naturale di Genova 16: 377-501

Jacoby M (1904) Descriptions of new genera and species of phytophagous Coleoptera obtained by Dr Loria in New Guinea. Annali del Museo Civico di Storia Naturale di Genova 41: 469-514

Jolivet P (1967) Notes systématiques et écologiques sur les Chrysomélides marocains (Coleoptera). Bulletin de la Société des Sciences naturelles et physiques du Maroc 46: 305-394

Kamiński E (1936) Über die Haliticinen-Fauna (Coleóptera) und ihre Nahrungspflanzen aus der Umgebung von Wilno. Travaux de l'Institut de Zoologie de l'Université de Wilno 34: 1-33

Konstantinov A, Tishechkin A (2004) The first Nearctic leaf litter flea beetle (Coleoptera: Chrysomelidae) from the Great Smoky Mountains National Park. The Coleopterists Bulletin 58: 71-76

Lea AM (1917) Insecta. Transactions of the Royal Society of South Australia XLI: 626-626

Leonardi C (1972) La “*Psylliodes wachsmanni*” Csiki specie distinta e suo inquadramento nel gruppo della “*Psylliodes picina”*. Atti del museo civico di storia naturale di Trieste 28: 139-146

Leonardi C (2007) Dati inediti sul genere *Psylliodes* Latreille, con descrizione di quattro nuove specie mediterranee (Coleoptera Chrysomelidae). Atti della Società italiana di Scienze naturali e del Museo civico di Storia naturale di Milano 148: 161-240

Leonardi C (2013) Indagine critica su *Psylliodes laevifrons* Kutschera con descrizione di due nuove specie (Coleoptera Chrysomelidae). Atti della Società italiana di Scienze naturali e del Museo civico di Storia naturale di Milano 154: 81-114

Leonardi C, Arnold U (1995) Due nuove specie di *Psylliodes* della regione mediterranea orientale (Coleoptera, Chrysomelidae). Atti della Società italiana di Scienze naturali e del Museo civico di Storia naturale di Milano 134: 299-311

Lopatin IK (2010) Leaf beetles (Insecta, Coleoptera, Chrysomelidae) of Central Asia. BSU, Minsk, 511 pp. (in Russian)

Medvedev LN (2004) New species of Leaf beetle (Insecta: Coleoptera: Chrysomelidae) from Nepal. In: Hartmann M, Baumbach H (Eds). Verein der Freunde und Förderer des Naturkundemuseums, Erfurt, 317-322

Mohamedsaid MS (2004) Catalogue of the Malaysian Chrysomelidae (Insecta: Coleoptera). Pensoft, Sofia-Moscow, 1-239 pp.

Mohr KH (1966) Chrysomelidae. In: Freude H, Harde KW, Lohse GA (Eds) Die Käfer Mitteleuropas Band 9. Goecke & Evers Verlag, Krefeld, 95-280

Nadein K, Lee C-f (2012) New data about some Alticini from Taiwan with descriptions of two new species (Coleoptera: Chrysomelidae). Bonn zoological Bulletin 61: 41-48

Nadein KS (2005) A review of leaf beetles of the *Psylliodes saulcyi* species-group (Coleoptera, Chrysomelidae, Alticinae). Entomological Review 85: 267-279

Nadein KS (2006) New species of the genus *Psylliodes* Latr. (Coleoptera, Chrysomelidae) from the Palaearctic Region. Entomological Review 86: 931-941. doi:10.1134/S0013873806080094

Nadein KS (2007a) On the taxonomy and classification of the genus *Psylliodes* Latreille, 1825 (Coleoptera, Chrysomelidae, Galerucinae). Entomologica Basiliensia et Collectionis Frey 29: 307-332

Nadein KS (2007b) Review of the *cucullatus* species group of the genus *Psylliodes* Latreille ( Coleoptera: Chrysomelidae: Galerucinae). Genus 18: 637-660

Nadein KS (2007c) Review of the *glaber* species-group of the genus *Psylliodes* Latr. (Coleoptera: Chrysomelidae: Galerucinae: Alticini). Genus 18: 433-471

Nadein KS (2008) Review of the *Psylliodes gibbosus* species group, with descriptions of two new species (Coleoptera: Chrysomelidae: Galerucinae). Koleopterologische Rundschau 78: 333-366

Nadein KS (2010) A review of the genus *Psylliodes* Latreille (Coleoptera, Chrysomelidae) from Russia and neighboring countries: II. An annotated list of species. Entomological Review 90: 1035-1074. doi:10.1134/S0013873810080099

Olegovich BA (2011) Beetles (Coleoptera, Chrysomelidae) European part of Russia. PhD Thesis, Lambert Academic Publishing, Dudweiler Landstrasse: Russian Academy of Science, Moscow.

Petitpierre E (1985) Notas faunisticas y ecológicas sobre Chrysomelidae (Coleoptera) de Mallorca y Catalunya. Bolletí de la Societat d'Història Natural de les Balears 29: 31-36

Petitpierre E (1999) Catàleg dels coleòpters crisomèlids de Catalunya IV. Alticinae. Butlletí de la Institució Catalana d'Historia Natural 67: 91-129

Peyerimhoff DP (1915) Notes sur la biologie de quelques Coléoptères phytophages du Nord Africain (2e série). Annales de la Société Entomologique de France 84: 19-61

Razzaque QMA (2002) Studies on the biology and ecology of the Duboisia flea beetle *Psylliodes paralis* Weise (Chrysomelidae, Coleoptera) in Duboisia plantations. Australian Journal of Entomology 41: 281-282

Rheinheimer J, Hassler M (2018) Die Blattkäfer Baden-Württembergs. Kleinsteuber Books, Karlsruhe, 928 pp.

Rozner I, Rozner G (2014) Data to the leaf-beetle fauna of Greece (Coleoptera: Chrysomelidae). Natura Somogyiensis 24: 81-98

Scherer G (1969) Die Alticinae des Indischen Subkontinentes (Coleoptera - Chrysomelidae). Pacific Insects Monographs 22: 1-251

Scherer G (1982) Erichson‐Typen im Zoologischen Museum Berlin (Coleoptera - Chrysomelidae - Alticinae). Deutsche Entomologische Zeitschrift 29: 479-481. doi:10.1002/mmnd.19820290412

Şen I, Gök A (2009) Leaf beetle communities (Coleoptera: Chrysomelidae) of two mixed forest ecosystems dominated by pine – oak – hawthorn in Isparta province, Turkey. Annales Zoologici Fennici 46: 217-232. doi:10.5735/086.046.0306

Takizawa H (2005) A revision of the Genus *Psylliodes* Latreille in Japan (Chrysomelidae: Alticinae). Insecta Matsumurana Series entomology New series 62: 175-185

Ugarte I (2005) Coleópteros fitófagos de los encinares cantábricos (Insecta: Coleoptera) de la reserva de la biosfera de Urdaibai. País Vasco - España, Asociación naturística AMALUR NATURA ELKARTEA. Agurain/ Salvatierra, Araba/ Álava), 197 pp.

Vela JM, Bastazo G, Fritzlar F (2017) Inventario comentado de los crisomélidos (Coleoptera, Chrysomelidae) de las Sierras Tejeda y Almijara y los Acantilados de Maro (Sur de España , Málaga- Granada). Boletin de la Asociación Española de Entomologia 41: 29-73

Wanntorp, H.-E. & Ødegaard, F. 2005. Tre missförstådda jordloppor: om förekomsten av *Psylliodes isatidis* Heikertinger, *P. crambicola* Lohse och *P. brisouti* (Bedel) i Norden (Coleoptera, Chrysomelidae) Entomologisk Tidskrift 126: 191-199

Weise J (1900) Beschreibungen von Chrysomeliden und Synonymische Bemerkungen. Archiv fur Naturgeschichte 66: 267-296

Weise J (1923) Chrysomeliden und Coccinelliden aus Queensland. Results of Dr. E. Mjöberg's Swedish Scientific Expedition to Australia 1910-1913. Arkiv för zoologi 15: 1-150

Wollaston TV (1854) Insecta Maderensia: being an account of the insects of the islands of the Madeiran group. J. Van Voorst, London, 634-634 pp. doi:10.5962/bhl.title.9060

Yano S, Ohsaki N (1993) The phenology and intrinsic quality of wild crucifers that determine the community structure of their herbivorous insects. Researches on Population Ecology 35: 151-170. doi:10.1007/BF02513589

Zverezomb-Zubovsky EV (1956) Pests of Sugar Beet. Academy of Sciences of Ukrainian SSR, Kiev, 276 pp. (in Russian)
