# Supplementary material for: Adaptation of flea beetles to Brassicaceae: host plant associations and geographic distribution of Psylliodes Latreille and Phyllotreta Chevrolat (Coleoptera, Chrysomelidae)
Source: Zookeys. 2019 Jun 17;856:51–73. doi: 10.3897/zookeys.856.33724 (PMC6603994; doi:10.3897/zookeys.856.33724)
Supplement: Supplementary material 2 [file zookeys-856-051-s002.docx]

**Supplementary Table 2.** Species groups of *Psylliodes* s. str. and their associated host plant families.

| ***Psylliodes* species group** | **Host plant family** | **Reference** |
| --- | --- | --- |
| ***chrysocephala* group** | | |
| *Ps. angusticeps* | Brassicaceae | Nadein 2006a |
| *Ps. chrysocephala* | Brassicaceae | Leonardi 1970; Nadein 2006b |
| *Ps. circumdata****^†^*** | Brassicaceae | Leonardi 1970 |
| *Ps. festae* | Brassicaceae | Doguet and Leonardi 2017 |
| *Ps. laticollis****^†^*** | Brassicaceae | Leonardi 1970 |
| *Ps. lethierryi* | Brassicaceae | Doguet and Leonardi 2017 |
| *Ps. marcida* | Brassicaceae | Leonardi 1970; Nadein 2006b |
| *Ps. pallidipennis* | Brassicaceae | Leonardi 1970 |
| *Ps. rhaica* | Unknown | Leonardi 1970; Nadein 2006b |
| *Ps. tricolor* | Brassicaceae | Nadein 2006b |
| ***cucullata* group** | | |
| *Ps. agropyri* | Poaceae | Nadein 2007c |
| *Ps. cucullata* | Poaceae, Caryophyllaceae, Polygonaceae, Brassicaceae | Leonardi 1970; Nadein 2006b |
| *Ps. olgae* | Unknown | Nadein 2007b |
| *Ps. pyrenaea* | Unknown | Nadein 2006b |
| ***gibbosa* group** | | |
| *Ps. fageli* | Unknown | Nadein 2008 |
| *Ps. gibbosa* | Poaceae | Leonardi 1970; Nadein 2006b |
| *Ps. gougeleti* | Poaceae | Nadein 2008 |
| *Ps. inflata* | Poaceae | Leonardi 1970; Nadein 2006b |
| *Ps. kiesenwetteri* | Brassicaceae | Leonardi 1970; Nadein 2006b |
| *Ps. ridenda* | Unknown | Nadein 2008 |
| *Ps. ruffoi* | Poaceae | Nadein 2006b |
| *Ps. tenuidentata* | Unknown | Nadein 2008 |
| ***hyoscyami* group** | | |
| *Ps. chalcomera* | Asteraceae | Leonardi 1970; Nadein 2006b |
| *Ps. dulcamarae* | Solanaceae | Leonardi 1970; Nadein 2006b |
| *Ps. hyoscyami* | Solanaceae | Leonardi 1970; Nadein 2006b |
| ***napi* group** | | |
| *Ps. brisouti* | Brassicaceae | Nadein 2006b |
| *Ps. crambicola****^†^*** | Brassicaceae | Leonardi 1970 |
| *Ps. cuprea****^†^*** | Brassicaceae | Leonardi 1970 |
| *Ps. fusiformis****^†^*** | Brassicaceae | Nadein 2007c |
| *Ps. isatidis****^†^*** | Brassicaceae | Leonardi 1970 |
| *Ps. laticollis****^†^*** | Brassicaceae | Nadein 2007c |
| *Ps. milleri****^†^*** | Brassicaceae | Leonardi 1970 |
| *Ps. napi* | Brassicaceae | Nadein 2006b |
| *Ps. submontana* | Unknown | Nadein 2007c |
| *Ps. thlaspis****^†^*** | Brassicaceae | Leonardi 1970 |
| *Ps. toelgi* | Brassicaceae | Leonardi 1970; Nadein 2006b |
| *Ps. urbaniae* | Brassicaceae | Biondi and D'Alessandro 2017 |
| *Ps. vindobonensis* | Brassicaceae | Nadein 2006b |
| ***picina* group** | | |
| *Ps. algirica****^†^*** | Fagaceae | Leonardi 1970 |
| *Ps. anatolica* | Fagaceae | Gök and Çilbiroğlu 2004 |
| *Ps. attenuata****^†^*** | Cannabaceae, Solanaceae, Asteraceae, Amaranthaceae, Linaceae, Fabaceae | Leonardi 1970 |
| *Ps. belarbii* | Unknown | Leonardi 2007 |
| *Ps. cerenae* | Unknown | Leonardi 2007 |
| *Ps. cervinoi* | Unknown | Baselga and Novoa 2003 |
| *Ps. cretica* | Fagaceae | Leonardi 1978; Nadein 2006b |
| *Ps. danieli****^†^*** | Unknown | Leonardi 1970 |
| *Ps. drusei* | Fagaceae | Nadein 2006b |
| *Ps. elliptica* | Unknown | Leonardi 2007 |
| *Ps. feroniae* | Fagaceae | Leonardi 1978; Nadein 2006b |
| *Ps. fiorellae* | Unknown | Leonardi 1978; Nadein 2006b |
| *Ps. frivaldszkyi****^†^*** | Unknown | Leonardi 1970 |
| *Ps. glabra****^†^*** | Unknown | Leonardi 1970 |
| *Ps. illyrica* | Fagaceae | Leonardi 1970; Nadein 2006b |
| *Ps. laevifrons* | Fagaceae | Leonardi 2013; Nadein 2006b |
| *Ps. leonhardi* | Poaceae, Fagaceae | Leonardi 1970 |
| *Ps. libanicola* | Unknown | Nadein 2006b |
| *Ps. luteola****^†^*** | Poaceae, Fagaceae, Solanaceae, Salicaceae, Ulmaceae, Lythraceae | Leonardi 1970 |
| *Ps. metatarsalis* | Unknown | Leonardi 2007 |
| *Ps. obscuroaenea* | Fagaceae | Nadein 2006b |
| *Ps. petasata****^†^*** | Caryophyllaceae | Leonardi 1970 |
| *Ps. picina* | Fagaceae | Leonardi 1970; Nadein 2006b |
| *Ps. puncticollis* | Poaceae, Asteraceae, Apocynaceae, Onagraceae | Leonardi 1970 |
| *Ps. schwarzi****^†^*** | Unknown | Leonardi 1970 |
| *Ps. springeri* | Brassicaceae | Leonardi 2007 |
| *Ps. sturanyi****^†^*** | Poaceae | Leonardi 1970 |
| *Ps. wachsmanni****^†^*** | Fagaceae | Leonardi 1972 |
| *Ps. yalvacensis* | Fagaceae | Gok 2005 |
| ***pyritosa* group** | | |
| *Ps. aerea****^†^*** | Brassicaceae | Leonardi 1970 |
| *Ps. biondii* | Brassicaceae | Leonardi 2007 |
| *Ps. caneparii* | Brassicaceae | Leonardi 2007 |
| *Ps. cereola* | Brassicaceae | Leonardi 2007 |
| *Ps. cupreata****^†^*** | Brassicaceae | Nadein 2006b |
| *Ps. fusiformis****^†^*** | Brassicaceae | Leonardi 1970 |
| *Ps. hispana* | Brassicaceae | Leonardi 1970 |
| *Ps. hospes****^†^*** | Brassicaceae | Leonardi 1970 |
| *Ps. instabilis* | Brassicaceae | Leonardi 1970; Nadein 2006b |
| *Ps. littoralis****^†^*** | Brassicaceae | Biondi 1997 |
| *Ps. maculatipes****^†^*** | Brassicaceae | Leonardi 2007 |
| *Ps. moricandiae* | Brassicaceae | Leonardi 2007 |
| *Ps. pallidicornis****^†^*** | Brassicaceae | Leonardi 1970 |
| *Ps. picipes****^†^*** | Brassicaceae | Leonardi 1970 |
| *Ps. pyritosa* | Brassicaceae | Leonardi 1970; Nadein 2006b |
| *Ps. subaenea****^†^*** | Brassicaceae | Leonardi 1970 |
| *Ps. valida****^†^*** | Brassicaceae | Leonardi 1970 |
| ***persica* group** | | |
| *Ps. arista* | Unknown | Nadein 2006b |
| *Ps. coelestis* | Unknown | Nadein 2006b |
| *Ps. deplanata* | Unknown | Nadein 2006b |
| *Ps. heikertingeri* | Brassicaceae | Nadein 2006b |
| *Ps. hospes****^†^*** | Brassicaceae | Nadein 2006b |
| *Ps. littoralis****^†^*** | Brassicaceae | Nadein 2006b |
| *Ps. milleri****^†^*** | Brassicaceae | Nadein 2006b |
| *Ps. persica* | Brassicaceae | Leonardi 1970; Nadein 2006b |
| ***affinis* group** | | |
| *Ps. affinis* | Solanaceae | Leonardi 1970; Nadein 2007c |
| *Ps. cupreata****^†^*** | Brassicaceae | Leonardi 1970 |
| *Ps. dilutella****^†^*** | Solanaceae | Leonardi 1970 |
| *Ps. grigorievi****^†^*** | Unknown | Leonardi 1970 |
| *Ps. saulcyi****^†^*** | Chenopodiaceae | Leonardi 1970 |
| ***vehemens* group** | | |
| *Ps. laurisilvae* | Brassicaceae | Biondi 1987 |
| *Ps. vehemens* | Brassicaceae | Leonardi 1970 |
| ***glabra* group** | | |
| *Ps. danieli****^†^*** | Unknown | Nadein 2006b |
| *Ps. dogueti* | Unknown | Nadein 2006b |
| *Ps. frivaldszkyi****^†^*** | Unknown | Nadein 2006b |
| *Ps. glabra****^†^*** | Unknown | Nadein 2006b |
| *Ps. longicollis* | Unknown | Nadein 2006b |
| *Ps. parodii* | Poaceae | Leonardi 2007 |
| *Ps. petasata****^†^*** | Caryophyllaceae | Nadein 2006b |
| *Ps. rubroaenea* | Unknown | Nadein 2006b |
| *Ps. schwarzi****^†^*** | Unknown | Nadein 2006b |
| *Ps. solarii* | Unknown | Nadein 2006b |
| *Ps. sturanyi****^†^*** | Poaceae | Nadein 2006b |
| ***cuprea* group** | | |
| *Ps. crambicola****^†^*** | Brassicaceae | Nadein 2006b |
| *Ps. cuprea****^†^*** | Brassicaceae | Nadein 2006b |
| *Ps. isatidis****^†^*** | Brassicaceae | Nadein 2006b |
| *Ps. ozisiki* | Unknown | Nadein 2006b |
| *Ps. pallidicornis****^†^*** | Brassicaceae | Nadein 2007c |
| *Ps. thlaspis****^†^*** | Brassicaceae | Nadein 2006b |
| *Ps. wrasei* | Brassicaceae | Nadein 2006b |
| ***saulcyi* group** | | |
| *Ps. analogica* | Unknown | Nadein 2006b |
| *Ps. astenica* | Unknown | Nadein 2006b |
| *Ps. dilutella****^†^*** | Solanaceae | Nadein 2006b |
| *Ps. grigorievi****^†^*** | Unknown | Nadein 2006b |
| *Ps. infanda* | Unknown | Nadein 2006b |
| *Ps. saulcyi****^†^*** | Chenopodiaceae | Nadein 2006b |
| ***luteola* group** | | |
| *Ps. algirica****^†^*** | Fagaceae | Nadein 2006b |
| *Ps. concolor* | Unknown | Nadein 2007c |
| *Ps. diversicolor* | Fagaceae | Nadein 2006a |
| *Ps. luteola****^†^*** | Poaceae, Fagaceae, Solanaceae, Salicaceae, Ulmaceae, Lythraceae | Nadein 2006b |
| *Ps. maculatipes****^†^*** | Brassicaceae | Nadein 2006b |
| *Ps. pallidicolor* | Fagaceae | Nadein 2006b |
| *Ps. ruficolor* | Fagaceae | Nadein 2006b |
| *Ps. wachsmanni****^†^*** | Fagaceae | Nadein 2006b |
| ***brettinghami* group** | | |
| *Ps. brettinghami* | Solanaceae | Nadein 2007c |
| *Ps. nitida* | Unknown | Nadein 2007c |
| *Ps. viridana* | Solanaceae | Nadein 2007c |
| ***punctifrons* group** | | |
| *Ps. amurensis* | Unknown | Nadein 2007c |
| *Ps. cyanescens* | Unknown | Nadein 2007c |
| *Ps. laxa* | Unknown | Nadein 2007c |
| *Ps. punctifrons* | Brassicaceae | Nadein 2007c |
| *Ps. subrugosa* | Brassicaceae | Nadein 2007c |
| *Ps. takizawai* | Brassicaceae | Nadein 2007c |
| ***aerea* group** | | |
| *Ps. aerea****^†^*** | Brassicaceae | Nadein 2007c |
| *Ps. picipes****^†^*** | Brassicaceae | Nadein 2007c |
| ***circumdata* group** | | |
| *Ps. circumdata****^†^*** | Brassicaceae | Nadein 2007c |
| ***aeneola* group** | | |
| *Ps. aeneola* | Unknown | Nadein 2006b |
| ***attenuata* group** | | |
| *Ps. attenuata****^†^*** | Cannabaceae, Solanaceae, Asteraceae, Amaranthaceae, Linaceae, Fabaceae | Nadein 2006b |
| ***testaceoconcolor* group** | | |
| *Ps. testaceoconcolor* | Brassicaceae | Nadein 2007c |
| ***subaenea* group** | | |
| *Ps. subaenea****^†^*** | Brassicaceae | Nadein 2006b |
| ***valida* group** | | |
| *Ps. valida****^†^*** | Brassicaceae | Nadein 2006b |
| ***altimontana* group** | | |
| *Ps. globosa* | Unknown | Nadein 2007a |
| *Ps. altimontana* | Unknown | Nadein 2007a |
| ***montana* group** | | |
| *Ps. afromontana* | Unknown | Biondi and D'Alessandro 2018 |
| *Ps. kikuyuana* | Unknown | Biondi 1996 |
| *Ps. manobioides* | Poaceae | Nadein 2007a |
| *Ps. masai* | Unknown | Biondi 1996 |
| *Ps. montana* | Unknown | Biondi 1996 |
| *Ps. shirensis* | Unknown | Biondi and D'Alessandro 2018 |
| ***calcarata* group** | | |
| *Ps. calcarata* | Unknown | Biondi 1996 |
| *Ps. teresae* | Solanaceae | Biondi 1996 |
| **marcosellai *group*** | | |
| *Ps. marcosellai* | Unknown | Biondi 1996 |

***^†^*** Indicates species classified in more than one group, depending on the study cited.

References are based on the original description and reclassification (if any occurred).

**REFERENCES**

Baselga A, Novoa F (2003) A new species of *Psylliodes* (Coleoptera: Chrysomelidae) and key to the wingless species from the Iberian Peninsula. Annals of the Entomological Society of America 96: 689-692. doi:10.1603/0013-8746(2003)096[0689:ANSOPC]2.0.CO;2

Biondi M (1987) Contributo alla conoscenza dei Chrysomelidae Alticinae delle isole Canarie , con descrizione di una nuova specie di *Psylliodes* (Coleoptera). Vieraea 17: 93-97

Biondi M (1996) The genus *Psylliodes* in the Afrotropical region with description of five new species from Kenya , Tanzania and South Africa. Fragmenta Entomologica 28: 257-276

Biondi M (1997) *Longitarsus aubozaorum* and *Psylliodes littoralis*, two new flea beetle species from Turkey. Fragmenta Entomologica, Roma 29: 383-390

Biondi M, D'Alessandro P (2017) *Psylliodes urbaniae* : a new species from Central Apennines (Coleoptera: Chrysomelidae: Galerucinae: Alticini). Onychium 13: 121-130. doi:10.5281/zenodo.495568

Biondi M, D'Alessandro P (2018) Two new species of the flea beetle genus *Psylliodes* Latreille of the *montana* species-group from Eastern Africa (Coleoptera: Chrysomelidae). Fragmenta Entomologica 50: 87-93. doi:doi.org/10.4081/fe.2018.305

Biondi M, D’Alessandro P (2019) *Psylliodes shirensis*, a new replacement name for *Psylliodes shira* Biondi & D’Alessandro (Coleoptera: Chrysomelidae). Fragmenta Entomologica 51 (in press).

Doguet S, Leonardi C (2017) Critical study on *Psylliodes circumdata* (W. Redtenbacher) with restoration of two species (Coleoptera: Chrysomelidae). Entomologische Blätter und Coleoptera 113: 55-66

Gök A (2005) *Psylliodes yalvacensis* sp. n. (Coleoptera, Chrysomelidae, Alticinae) from Turkey. Biologia Bratislava 60: 133-135. doi:10.1080/13880200590903354

Gök A, Çilbiroğlu EG (2004) A new species of the genus *Psylliodes* Latreille (Coleoptera: Chrysomelidae) from Turkey. Zootaxa 440: 1-6

Leonardi C (1970) Materiali per uno studio filogenetico del genere *Psylliode*s Coleoptera Chrysomelidae). Atti della Società italiana di scienze naturali e del Museo civico di storia naturale di Milano 110: 201-223

Leonardi C (1972) La “*Psylliodes wachsmanni*” Csiki specie distinta e suo inquadramento nel gruppo della “*Psylliodes picina”*. Atti del museo civico di storia naturale di Trieste 28: 139-146

Leonardi C (1978) Studio critico sulla *Psylliodes picina* (Marsh.) e sulle forme che le sono state attribuite, con particolare riguardo alla fauna italiana (Coleoptera Chrysomelidae). Atti della Società italiana di Scienze naturali e del Museo civico di Storia naturale di Milano 119: 271-299.

Leonardi C (2007) Dati inediti sul genere *Psylliodes* Latreille, con descrizione di quattro nuove specie mediterranee (Coleoptera Chrysomelidae). Atti della Società italiana di Scienze naturali e del Museo civico di Storia naturale di Milano 148: 161-240

Leonardi C (2013) Indagine critica su *Psylliodes laevifrons* Kutschera con descrizione di due nuove specie (Coleoptera Chrysomelidae). Atti della Società italiana di Scienze naturali e del Museo civico di Storia naturale di Milano 154: 81-114

Nadein KS (2006a) New species of the genus *Psylliodes* Latr. (Coleoptera, Chrysomelidae) from the Palaearctic Region. Entomological Review 86: 931-941. doi:10.1134/S0013873806080094

Nadein KS (2006b) A significance of the tegmen structure for classification of the genus *Psylliodes* Latreille, 1829 (Coleoptera: Chrysomelidae: Psylliodina). Proceedings of the Russian Entomological Society St. Petersburg 77: 250-254

Nadein KS (2007a) On the taxonomy and classification of the genus *Psylliodes* Latreille, 1825 (Coleoptera, Chrysomelidae, Galerucinae). Entomologica Basiliensia et Collectionis Frey 29: 307-332

Nadein KS (2007b) Review of the *cucullatus* species group of the genus *Psylliodes* Latreille (Coleoptera: Chrysomelidae: Galerucinae). Genus 18: 637-660

Nadein KS (2007c) A review of the leaf-beetle genus *Psylliodes* Latreille (Coleoptera, Chrysomelidae) from Russia and neighboring countries: I. A key to subgenera, species-groups, and species. Entomological Review 87: 330-360. doi:10.1134/S0013873807030086

Nadein KS (2008) Review of the *Psylliodes gibbosus* species group, with descriptions of two new species (Coleoptera: Chrysomelidae: Galerucinae). Koleopterologische Rundschau 78: 333-366
