# Supplementary material for: Adaptation of flea beetles to Brassicaceae: host plant associations and geographic distribution of Psylliodes Latreille and Phyllotreta Chevrolat (Coleoptera, Chrysomelidae)
Source: Zookeys. 2019 Jun 17;856:51–73. doi: 10.3897/zookeys.856.33724 (PMC6603994; doi:10.3897/zookeys.856.33724)
Supplement: Supplementary material 3 [file zookeys-856-051-s003.docx]

**Supplementary Table 3.** List of *Phyllotreta* species including their food plants, diet breadth and geographical distribution.

| **Species** | **Food Plants ^1^** | **Food plant family ^2^** | **Diet breadth ^3^** | **Location ^4^** | **Geographic Region** | **References ^5^** |
| --- | --- | --- | --- | --- | --- | --- |
| *Phyllotreta acutecarinata*  Heikertinger, 1941 | *Brassica napus* | Brassicaceae | MON | Austria, Greece (Thessaloniki), Poland, Russia, Ukraine, Afghanistan, Turkey | PAR | Ghahari 2017 |
| *Phyllotreta aeneicollis*  (Crotch, 1873) | *Brassica* spp., *Coronopus didymus, Lepidium virginicum, L. austrinum, Sisymbrium irio, Raphanus sativus, Cleome* spp*.* | Brassicaceae, Cleomaceae | OLI | North America | NAR | Clark et al. 2004 |
| *Phyllotreta africana*  Scherer, 1970 | Unknown | Unknown | Unknown | South Africa (Cape Province) | AFR | Scherer 1970 |
| *Phyllotreta alberta*  Chittenden, 1927 | *Lepidium virginicum* | Brassicaceae | MON | North America | NAR | Clark et al. 2004 |
| *Phyllotreta albionica*  (LeConte, 1857) | *Brassica* spp., *Capsella bursa-pastoris, Descurainia sophia, Draba streptocarpa, Iberis* spp*., Lepidium alyssoides, L. montanum, Lobularia maritima, Raphanus sativus, Nasturtium officinale, Sisymbrium officinale, Thlaspi alpestre, Cleome integrifolia, C. lutea, Tropaeolum majus* | Brassicaceae, Cleomaceae, Tropaeolaceae | OLI | North America, Mexico | NAR, NTR | Clark et al. 2004 |
| *Phyllotreta amabilis*  Weise, 1903 | Unknown | Unknown | Unknown | Tanzania, Rwanda, Burundi | AFR | Heikertinger 1943 |
| *Phyllotreta andreevae*  Lopatin, 1992 | Unknown | Unknown | Unknown | Tajikistan | PAR | Döberl 2010 |
| *Phyllotreta annae*  Konstantinov, 1992 | Unknown | Unknown | Unknown | Kazakhstan | PAR | Döberl 2010 |
| *Phyllotreta aptera*  Wang, 1992 | Unknown | Unknown | Unknown | China (Yunnan) | ORR | Döberl 2010 |
| *Phyllotreta araxicola*  Iablokoff-Khnzorian, 1968 | Unknown | Unknown | Unknown | Azerbaijan, Armenia | PAR | Döberl 2010 |
| *Phyllotreta arcuate*  Smith, 1985 | Unknown | Unknown | Unknown | North America | NAR | Smith 1985 |
| *Phyllotreta armoraciae*  (Koch, 1803) | *Armoracia rusticana,* (also reported on *Nasturtium officinale*) | Brassicaceae | MON | Europe, Kazakhstan (Turkestan); Nearctic Region | PAR, NAR | Chashchina 2008; Rheinheimer and Hassler 2018 |
| *Phyllotreta astrachanica*  Lopatin, 1977 | *Raphanus sativus, Neslia paniculata, Lepidium perfoliatum, L. draba, Rorippa islandica, Sinapis* spp.*, Cardamine* spp., *Armoracia rusticana, Nasturtium* spp., *Alliaria* spp. | Brassicaceae | OLI | Azerbaijan, Austria, Bulgaria, Czech Republic, France, Georgia, Greece, Germany, Hungary, Italy, Netherlands, Poland, Slovakia, Slovenia, Spain, Switzerland, Sweden, Serbia and Montenegro, Cyprus, Iran, Kazakhstan, Turkey | PAR | Baviera and Biondi 2015; Brelih et al. 2003; Doguet 1994; Şen and Gök 2009; Rheinheimer and Hassler 2018 |
| *Phyllotreta asturica*  Heikertinger, 1941 | Unknown | Unknown | Unknown | Spain | PAR | Döberl 2010 |
| *Phyllotreta atra*  (Fabricius, 1775) | *Brassica rapa, Alliaria petiolata, Thlaspis perfoliatum, Cardamine impatiens, Raphanus sativus, Capsella bursa-pastoris, Sinapis* spp*., Arabis hirsuta, Diplotaxis muralis, Armoracia rusticana, Nasturtium* spp., *Lepidium* spp.*, Reseda lutea, Cleome speciosissima, Tropaeolum majus* | Brassicaceae, Resedaceae, Cleomaceae, Tropaeolaceae | OLI | Europe, Algeria, Morocco, Afghanistan, Russia (Eastern), Iran, Jordan, Kazakhstan, Mongolia, Syria, Tajikistan, Kyrgyzstan, Yemen, Turkey | PAR, AFR | Aslan and Alkan 2015; Aslan and Ghahari 2017; Baviera and Biondi 2015; Brelih et al. 2003; Chashchina 2008; Rheinheimer and Hassler 2018 |
| *Phyllotreta attenuata*  Smith, 1985 | *Rorippa palustris* | Brassicaceae | MON | North America | NAR | Clark et al. 2004 |
| *Phyllotreta austriaca aligera*  Heiketinger, 1911 | Unknown | Unknown | Unknown | Russia (Eastern), Korea | PAR | Döberl 2010 |
| *Phyllotreta austriaca austriaca*  Heikertinger, 1909 | *Biscutella laevigata, Sisymbrium strictissimum, Hesperis matronalis, Lunaria rediviva* | Brassicaceae | OLI | Czech Republic, Germany, Hungary, Romania, Slovakia, Russia, Ukraine, Austria | PAR | Mohr 1966; Januš 2004; Fritzlar 2009; Rheinheimer and Hassler 2018 |
| *Phyllotreta aygulae*  Özdikmen et al., 2017 | *Brassica oleracea, Eruca vesicaria, Lepidium sativum* | Brassicaceae | OLI | Turkey | PAR | Özdikmen et al. 2017b |
| *Phyllotreta bactriana*  Heikertinger, 1941 | *Capparis* spp*., Malcolmia* spp*.* | Capparaceae, Brassicaceae | OLI | Tajikistan, Uzbekistan, Kazakhstan (Turkestan) | PAR | Lopatin 2010 |
| *Phyllotreta balcanica*  Heikertinger, 1909 | *Lepidium draba, Diplotaxis muralis, Raphanus* spp*., Sinapis arvensis, Rorippa sylvestris* | Brassicaceae | OLI | Azerbaijan, Albania, Austria, Bosnia-Herzegovina, Bulgaria, Croatia, France, Greece, Hungary, Macedonia, Italy, Romania, Slovenia, Spain, Ukraine, Serbia and Montenegro, Afghanistan, Kazakhstan, Turkey, Uzbekistan, Kyrgyzstan, Iran | PAR | Brelih et al. 2003; Doguet 1994; Doguet and Dufay 1994; Ghahari 2017 |
| *Phyllotreta banghaasi*  Heikertinger, 1941 | Unknown | Unknown | Unknown | Kazakhstan, Uzbekistan, Turkmenistan, Kyrgyzstan | PAR | Döberl 2010 |
| *Phyllotreta bartanga*  Lopatin, 1966 | Unknown | Unknown | Unknown | Tajikistan | PAR | Döberl 2010 |
| *Phyllotreta basalis*  Brisout de Barneville, 1884 | Unknown | Unknown | Unknown | Algeria | PAR | Döberl 2010 |
| *Phyllotreta beschkentica*  Lopatin, 1961 | Unknown | Unknown | Unknown | Tajikistan, Iran | PAR | Döberl 2010 |
| *Phyllotreta bilgeae*  Özdikmen & Coral Şahin, 2017 | Unknown | Unknown | Unknown | Turkey | PAR | Özdikmen and Coral Şahin 2017; Özdikmen et al. 2017a |
| *Phyllotreta bipustulata*  (Fabricius, 1801) | *Armoracia rusticana, Barbarea vulgaris, Brassica* spp.*, Capsella bursa-pastoris, Cardamine* spp.*, Lepidium campestre, L. virginicum, Raphanus sativus, Rorippa islandica, R. palustris, Sinapis arvensis, Sisymbrium officinale* | Brassicaceae | OLI | North America | NAR | Clark et al. 2004 |
| *Phyllotreta birmanica*  Harold 1877 | *Brassica* spp. | Brassicaceae | MON | Myanmar, India | ORR | Gajendra and Prasad 2016; Kalaichelvan and Verma 2005 |
| *Phyllotreta bisinuata*  Smith, 1985 | Unknown | Unknown | Unknown | North America | NAR | Smith 1985 |
| *Phyllotreta bolognai*  Biondi, 1992 | *Alyssum* spp*.* | Brassicaceae | MON | Turkey | PAR | Biondi 1992; Döberl 2010 |
| *Phyllotreta brevipennis*  Chittenden, 1927 | Unknown | Unknown | Unknown | North America | NAR | Chittenden 1927 |
| *Phyllotreta buchtarmensis*  Lopatin, 1990 | Unknown | Unknown | Unknown | Kazakhstan | PAR | Döberl 2010 |
| *Phyllotreta buettikeri*  Doguet, 1979 | Unknown | Unknown | Unknown | Algeria, Saudi Arabia | PAR | Döberl 2010 |
| *Phyllotreta buhseae*  Iablokoff-Khnzorian, 1978 | *Cleome coluteoides* | Cleomaceae | MON | Turkmenistan | PAR | Iablokoff-Khnzorian, 1978; Döberl 2010 |
| *Phyllotreta bulgarica*  Gruev, 1977 | *Barbarea minor* | Brassicaceae | Unknown | Bulgaria, Greece, Israel, Turkey | PAR | Furth 1979; Döberl 2010 |
| *Phyllotreta cadabae*  Bryant, 1942 | *Cadaba rotundifolia* | Capparaceae | Unknown | Sudan | AFR | Bryant 1942 |
| *Phyllotreta capensis*  Jacoby, 1990 | Unknown | Unknown | Unknown | South Africa | AFR | Heikertinger 1943 |
| *Phyllotreta capicola*  Bryant ,1924 | *Brassica oleracea* | Brassicaceae | Unknown | Sudan | AFR | Heikertinger 1943 |
| *Phyllotreta caucasicola*  Heikertinger, 1941 | *Brassica oleracea* | Brassicaceae | Unknown | Russia (Kavkaz), Armenia, Iraq, Turkey | PAR | Ghahari 2017 |
| *Phyllotreta chalybeipennis*  (Crotch, 1873) | *Cakile edentula* | Brassicaceae | MON | North America | NAR | Clark et al. 2004 |
| *Phyllotreta cheiranthi*  Weise, 1903 | *Brassica oleracea, B. rapa, Eruca sativa, Farsetia stenoptera, Raphanus sativus, Schouwia arabica, Cadaba rotundifolia, Cleome gynandra* | Brassicaceae, Capparaceae, Cleomaceae | OLI | Egypt, Arab Emirates, Oman, Saudi Arabia, Yemen, Sri Lanka, Madagascar, Uganda, Guinea, Sudan (Eastern), Tanzania | PAR, ORR, AFR | Bechyné 1955; Couilloud 1993; Heikertinger 1943 |
| *Phyllotreta chotanica*  Duvivier, 1892 | *Brassica* spp.*, Raphanus sativus, Cleome rutidosperma, C. gynandra* | Brassicaceae, Cleomaceae | OLI | Nepal, India (Northern), Pakistan, China (Yunnan), Vietnam (South), Taiwan | ORR | Lee et al. 2011; Ravi et al. 2014; Yadav et al. 2010 |
| *Phyllotreta christinae*  Heikertinger, 1941 | *Cardamine* spp. | Brassicaceae | MON | Austria, Bosnia-Herzegovina, Bulgaria, Croatia, Czech Republic, France, Germany, Hungary, Italy (Northern), Luxembourg, Netherlands, Poland, Romania, Slovakia, Slovenia, Switzerland, Ukraine | PAR | Brelih et al. 2003; Doguet 1994; Rheinheimer and Hassler 2018 |
| *Phyllotreta chujoe*  Madar, 1959 | *Cardamine* spp. | Brassicaceae | MON | Japan (Hokkaido, Honshu), Russia (Eastern) | PAR | Yano and Ohsaki 1993 |
| *Phyllotreta cleomica*  Furth, 1979 | *Cleome arabica, C. trinervia* | Cleomaceae | MON | Israel | PAR | Furth 1979 |
| *Phyllotreta coiffaiti*  Doguet, 1979 | Unknown | Unknown | Unknown | Lebanon | PAR | Döberl 2010 |
| *Phyllotreta collaris* Chapuis, 1879 | Unknown | Unknown | Unknown | Ethiopia | AFR | Heikertinger 1943 |
| *Phyllotreta conjuncta*  Gentner, 1924 | *Arabis* spp*., Armoracia rusticana, Barbarea verna, B. vulgaris, Lepidium* spp*., Raphanus sativus, Nasturtium officinale, Rorippa palustris, Sinapis arvensis, Brassica* spp. | Brassicaceae | OLI | North America | NAR | Clark et al. 2004 |
| *Phyllotreta consobrina consobrina*  (Curtis, 1837) | *Brassica* spp., *Diplotaxis* spp., *Isatis* spp., *Rapistrum* spp., *Sinapis* spp.,  *Eruca vesicaria, Sisymbrium* spp., *Cardamine* spp. | Brassicaceae | OLI | Belgium, Croatia (Dalmatia), France, United Kingdom, Germany, Italy, Luxembourg, Malta, Netherlands, Spain, Switzerland, Algeria, Morocco, Tunisia, Portugal (incl. Madeira); Afrotropical Region | PAR, AFR | Petitpierre 1999; Rheinheimer and Hassler 2018 |
| *Phyllotreta consobrina springeri*  Wittmer, 1936 | *Schouwia schimperi, Diplotaxis harra, Moricandia nitens* | Brassicaceae | OLI | Israel, Jordan, Lebanon, Egypt (Sinai) | PAR | Furth 1979 |
| *Phyllotreta constricta*  Smith, 1985 | *Cleome serrulata, C. integrifolia, Brassica rapa, Lepidium alyssoides, L. montanum, Rorippa palustris* | Cleomaceae, Brassicaceae | OLI | North America | NAR | Clark et al. 2004 |
| *Phyllotreta corrugata*  Reiche & Saulcy, 1858 | *Brassica napus, B. nigra, Sinapis arvensis,*  *S. alba, Lepidium draba, Diplotaxis* spp.*, Raphanus raphanistrum, R. sativus, Hirschfeldia incana, Erucaria hispanica, E. boveana, Isatis aleppica,*  *I. lucitanica, Barbarea minor, Enarthrocarpus strangulatus, Ochthodium aegyptiacum, Iberis* spp*., Reseda luteola, Tropaeolaceae* spp*.* | Brassicaceae, Resedaceae, Tropaeolaceae | OLI | Azerbaijan, Armenia, Bulgaria, France, Great Britain, Greece, Italy, Malta, Spain, Russia (Southern), Ukraine, Algeria, Egypt, Lebanon, Morocco, Tunisia, Afghanistan, Cyprus, Iran, Iraq, Israel, Jordan, Kazakhstan, Syria, Turkey, Turkmenistan, Uzbekistan | PAR | Aslan and Ghahari 2017; Baviera and Biondi 2015; Petitpierre 1999; Peyerimhoff 1915; Şen and Gök 2009 |
| *Phyllotreta costulata*  Weise, 1904 | Unknown | Unknown | Unknown | Tanzania | AFR | Heikertinger 1943 |
| *Phyllotreta crassicornis*  Allard, 1866 | *Matthiola* spp., Brassicaceae | Brassicaceae | OLI | Belgium, France, Italy, Luxembourg, Netherlands, Spain, Algeria, Morocco | PAR | Baviera and Biondi 2015; Heikertinger 1941 |
| *Phyllotreta crotchi*  Jacoby, 1885 | Unknown | Unknown | Unknown | Mexico | NTR | Jacoby 1885 |
| *Phyllotreta cruciferae*  (Goeze, 1777) | *Brassica* spp., *Sinapis* spp*., Alliaria* spp*., Eruca* spp*., Cochlearia* spp*., Rorippa* spp*., Camelina* spp*., Neslia* spp*., Hirschfeldia* spp*., Diplotaxis muralis, Arabis hirsuta, Cakile maritima, Sisymbrium officinale, Raphanus* spp*., Armoracia* spp., *Cardamine* spp., *Nasturtium* spp., *Lepidium* spp., *Isatis* spp., *Alyssum* spp., *Reseda* spp.*, Tropaeolum* spp. | Brassicaceae, Resedaceae, Tropaeolaceae | OLI | Europe, Algeria, Morocco, Tunisia, Egypt, Afghanistan, Cyprus, Iran, Israel, Japan (Kyushu), Jordan, Kazakhstan, Mongolia, Pakistan, Syria, Kyrgyzstan, Tajikistan, Turkey, India; Eritrea, Kenya (coastal); Nearctic Region | PAR, ORR, AFR, NAR | Baviera and Biondi 2015; Brelih et al. 2003; Clark et al. 2004; Furth 1979; Heikertinger 1943; Mohr 1966; Şen and Gök 2009; Rheinheimer and Hassler 2018 |
| *Phyllotreta cruralis*  Abeille de Perrin 1895 | *Suaeda pruinosa, Suaeda vera, Salsola kali,*  *S. vermiculata, Atriplex halimus* | Amaranthaceae | OLI | Spain, Algeria, Tunisia, Israel, Jordan | PAR | Furth 1979; Heikertinger 1941; Petitpierre et al. 2000 |
| *Phyllotreta cumingi*  Baly 1877 | Unknown | Unknown | Unknown | Philippines (Manila) | ORR | Baly 1877 |
| *Phyllotreta cupreata*  Chen & Kung, 1955 | Unknown | Unknown | Unknown | China (Gansu) | PAR | Döberl 2010 |
| *Phyllotreta curvipes*  Heikertinger, 1941 | Unknown | Unknown | Unknown | Kazakhstan | PAR | Döberl 2010 |
| *Phyllotreta cyanea*  Medvedev, 2009 | Unknown | Unknown | Unknown | Vietnam (Tam Dao) | ORR | Medvedev 2009 |
| *Phyllotreta cyanella*  Boheman 1859 | Unknown | Unknown | Unknown | Java | ORR | Boheman 1859 |
| *Phyllotreta dacica*  Heikertinger, 1941 | Unknown | Unknown | Unknown | Azerbaijan, Bosnia-Herzegovina, Bulgaria, Croatia, Romania, Ukraine, Serbia and Montenegro, Turkey | PAR | Döberl 2010 |
| *Phyllotreta decipiens*  Horn, 1889 | *Brassica rapa, Raphanus sativus, Beta vulgaris, Solidago* spp*., Artemisia* spp*., Phaseolus vulgaris, Solanum tuberosum* | Brassicaceae, Amaranthaceae, Asteraceae, Fabaceae, Solanaceae | POL | North America | NAR | Clark et al. 2004 |
| *Phyllotreta decora*  Boheman 1859 | Unknown | Unknown | Unknown | Philippines (Manila) | ORR | Boheman 1858; 1859 |
| *Phyllotreta denticornis*  Horn, 1889 | *Brassica* spp. | Brassicaceae | OLI | North America | NAR | Clark et al. 2004 |
| *Phyllotreta diademata*  Foudras, 1860 | *Lepidium perfoliatum, L. draba, Rorippa sylvestris, R. amphibia, Neslia paniculata, Brassica oleraceae, Alliaria officinalis, A. petiolata, Eruca vesicaria, Diplotaxis muralis, Arabis hirsuta, Raphanus* spp., *Isatis* spp., *Cardamine flexuosa,*  *C. pratensis, Armoracia rusticana, Crambe maritima, Sinapis* spp., *Lobularia maritima, Capparis spinosa* | Brassicaceae,  Capparaceae | OLI | Europe, Afghanistan, Iran, Iraq, India, Turkey, Kazakhstan (Turkestan) | PAR, ORR | Brelih et al. 2003; Cox 2007; Petitpierre 1999; Scherer 1969; Rheinheimer and Hassler 2018 |
| *Phyllotreta dilatata*  C. G. Thomson, 1866 | *Rorippa amphibia, R. palustris, Armoracia rusticana, Nasturtium* spp*., Lepidium latifolium* | Brassicaceae | OLI | Austria, Belgium, Belarus, Czech Republic, Denmark, France, Germany, Luxembourg, Netherlands, Poland, Slovakia, Sweden, Switzerland, Ukraine, Russia (Eastern) | PAR | Böhme 2005; Bukejs 2008; Doguet 1994; Mohr 1966; Verdyck 1998; Rheinheimer and Hassler 2018 |
| *Phyllotreta djurdjurensis*  Doguet, 1977 | Brassicaceae | Brassicaceae | Unknown | Algeria | PAR | Doguet 1977; Döberl 2010 |
| *Phyllotreta dohrniana*  Wollaston, 1867 | Unknown | Unknown | Unknown | Cape Verde | AFR | Wollaston, 1867; Heikertinger 1943 |
| *Phyllotreta dolichophalla*  Smith, 1985 | Unknown | Unknown | Unknown | North America | NAR | Smith, 1985 |
| *Phyllotreta dollmani*  Bryant, 1942 | Unknown | Unknown | Unknown | Zambia | AFR | Bryant 1942 |
| *Phyllotreta downesi*  Baly, 1877 | Unknown | Unknown | Unknown | Vietnam, Laos, South India | ORR | Medvedev 2009 |
| *Phyllotreta dunbrodensis*  (Jacoby, 1903) | Unknown | Unknown | Unknown | South Africa (Cape Province) | AFR | Biondi 1999 |
| *Phyllotreta ebneri*  Weise, 1925 | *Gossypium* spp. | Malvaceae | Unknown | Sudan | AFR | Heikertinger 1943 |
| *Phyllotreta egridirensis*  Gruev & Kasap, 1985 | Unknown | Unknown | Unknown | Iran, Turkey | PAR | Döberl 2010 |
| *Phyllotreta elongatula*  Boheman, 1859 | Unknown | Unknown | Unknown | Philippines (Manila) | ORR | Boheman 1858; 1859 |
| *Phyllotreta emarginata*  Smith, 1985 | Brassicaceae | Brassicaceae | OLI | North America | NAR | Clark et al. 2004 |
| *Phyllotreta erysimi baicalica*  Heikertinger, 1941 | Unknown | Unknown | Unknown | Mongolia, Russia (Eastern) | PAR | Döberl 2010 |
| *Phyllotreta erysimi erysimi*  Weise, 1900 | *Brassica napus, Lepidium draba, Sisymbrium conferta, Raphanus* spp. | Brassicaceae | OLI | Azerbaijan, Bulgaria, Greece, Macedonia, Romania, Russia, Turkey, Ukraine, Serbia and Montenegro, Iran, Afghanistan, Kyrgyzstan, Kazakhstan, Mongolia, Syria, Tajikistan, Turkey, Turkmenistan, Uzbekistan | PAR | Aslan and Alkan 2015; Aslan and Ghahari 2017; Furth 1979 |
| *Phyllotreta erysimi iranella*  Lopatin, l990 | Unknown | Unknown | Unknown | Iran | PAR | Döberl 2010 |
| *Phyllotreta erysimi kutscherai*  Heikertinger, 1941 | Unknown | Unknown | Unknown | Russia (Eastern) | PAR | Döberl 2010 |
| *Phyllotreta erysimi tekensis*  Lopatin, 1992 | Unknown | Unknown | Unknown | Turkmenistan | PAR | Döberl 2010 |
| *Phyllotreta exclamationis*  (Thunberg, 1784) | *Cardamine* spp*., Rorippa* spp*., Nasturtium* spp*.* | Brassicaceae | OLI | Europe | PAR | Baviera and Biondi 2015; Brelih et al. 2003; Cox 2007; Doguet 1994; Mohr 1966; Rheinheimer and Hassler 2018 |
| *Phyllotreta ezoensis*  Kimoto, 1993 | Unknown | Unknown | Unknown | Russia (Eastern), Japan (Hokkaido) | PAR | Döberl 2010 |
| *Phyllotreta fallaciosa*  Heikertinger, 1941 | *Sinapis* spp*., Diplotaxis* spp*., Erucaria* spp*., Hirschfeldia* spp*., Isatis* spp*., Nasturtium officinale* | Brassicaceae | OLI | France, Greece (Rhodes), Italy, Spain, Algeria, Morocco, Cyprus, Iran, Israel, Jordan, Turkey | PAR | Baviera and Biondi 2015; Furth 1979; Petitpierre 1999 |
| *Phyllotreta farsetiarum*  Peyerimhoff, 1929 | *Farsetia* spp*.* | Brassicaceae | OLI | Algeria; Afrotropical Region | PAR, AFR | Heikertinger 1941 |
| *Phyllotreta flavifrons*  Jacoby, 1899 | *Hibiscus esculentus* | Malvaceae | Unknown | Yemen, Somalia, Rwanda, Burundi, Tanzania | AFR | Bryant 1942 |
| *Phyllotreta flavilabris*  Weise, 1895 | Unknown | Unknown | Unknown | Senegal, Ghana | AFR | Bryant 1942; Heikertinger 1943; Bechyné 1955 |
| *Phyllotreta flavoguttata*  Kutschera, 1860 | *Sinapis alba, Hirschfeldia incana* | Brassicaceae | OLI | Albania, Greece, Israel | PAR | Furth 1979 |
| *Phyllotreta flexuosa*  (Illiger, 1794) | *Cardamine flexuosa, C. amara, Arabidopsis arenosa, Rorippa amphibia , Barbarea vulgaris, Raphanus* spp*., Nasturtium officinale, Armoracia rusticana, Arabis soyeri* | Brassicaceae | OLI | Europe | PAR | Brelih et al. 2003; Cox 2007; Doguet 1994; Rheinheimer and Hassler 2018 |
| *Phyllotreta florieni*  Pic, 1910 | *Diplotaxis harra, D. acris, Erucaria boveana,*  *E. pinnata* | Brassicaceae | OLI | Egypt, Arab Emirates, Israel, Saudi Arabia | PAR | Furth 1979 |
| *Phyllotreta fornuseki*  Cizek, 2003 | *Crambe tataria, Sisymbrium* spp. | Brassicaceae | OLI | Czech Republic, Slovakia, Turkey | PAR | Čížek 2003 |
| *Phyllotreta foudrasi*  Brisout de Barneville, 1873 | *Alyssum* spp*., Cakile maritima, Cardaria* spp*., Diplotaxis* spp*., Iberis* spp*., Lepidium* spp*.* | Brassicaceae | OLI | France, Italy, Portugal, Spain, Algeria, Morocco, Tunisia | PAR | Petitpierre 1999; Petitpierre et al. 2000 |
| *Phyllotreta fulgida*  Chittenden, 1927 | *Isomeris arborea* | Capparaceae | MON | North America | NAR | Krupnick and Weis 1998; Krupnick and Weis 1999 |
| *Phyllotreta fulvicollis*  Jacoby, 1885 | Unknown | Unknown | Unknown | Australia | AUR | Döberl 2010 |
| *Phyllotreta fulvonigra*  Laboissiére, 1942 | Unknown | Unknown | Unknown | Democratic Republic of Congo (Ndeko) | AFR | Laboissiére 1942 |
| *Phyllotreta furthi*  Doguet, 1984 | Unknown | Unknown | Unknown | Algeria, Tunisia | PAR | Döberl 2010 |
| *Phyllotreta gafsana*  Normand, 1937 | Unknown | Unknown | Unknown | Tunisia | PAR | Döberl 2010 |
| *Phyllotreta gallica*  Brisout de Barneville, 1892 | *Iberis amara* | Brassicaceae | MON | France, Spain, Switzerland | PAR | Petitpierre 1999 |
| *Phyllotreta ganglbaueri*  Heikertinger, 1909 | *Biscutella laevigata, Erysimum silvestre, Peltaria alliacea, Alyssum ovirense, Diplotaxis muralis, Arabis hirsuta, A. glabra, Iberis linifolia* | Brassicaceae | OLI | Austria, Bosnia-Herzegovina, Bulgaria, Croatia, Czech Republic, France (Alpes-Maritimes), Italy, Portugal, Romania, Slovenia, Ukraine (Crimea), Serbia and Montenegro, Turkey | PAR | Brelih et al. 2003; Mohr 1966 |
| *Phyllotreta gillerforsi*  Biondi, 1991 | *Brassica* spp. | Brassicaceae | OLI | Spain (end. Canary Islands) | PAR | Biondi 1991, 1995 |
| *Phyllotreta gloriae*  Biondi, 1994 | *Sinapis* spp*., Moricandia arvensis* | Brassicaceae | OLI | Spain | PAR | Biondi 1994; Vela et al. 2017 |
| *Phyllotreta gurskii*  Lopatin, 1966 | Unknown | Unknown | Unknown | Tajikistan | PAR | Döberl 2010 |
| *Phyllotreta hebraea*  Heikertinger, 1941 | *Erucaria boveana* | Brassicaceae | MON | Israel | PAR | Heikertinger 1941; Furth 1979 |
| *Phyllotreta hemipoda*  Abeille de Perrin, 1909 | *Moricandia* spp*., Lonchophora* spp. | Brassicaceae | OLI | Portugal, Algeria, Morocco, Tunisia | PAR | Heikertinger 1941 |
| *Phyllotreta herbacea*  Chittenden, 1927 | *Brassica rapa* | Brassicaceae | MON | North America | NAR | Clark et al. 2004 |
| *Phyllotreta hermonensis*  Furth, 1979 | *Anchonium billardieri, Erysimum goniocaulon* | Brassicaceae | OLI | Israel, Iran | PAR | Furth 1979 |
| *Phyllotreta hispanica*  Pic, 1903 | Unknown | Unknown | Unknown | Spain (South) | PAR | Döberl 2010 |
| *Phyllotreta hochetlingeri* Fleischer, 1917 | Unknown | Unknown | Unknown | Croatia, Poland, Slovakia | PAR | Döberl 2010 |
| *Phyllotreta humilis*  Weise, 1887 | Brassicaceae | Brassicaceae | Unknown | Russia (Eastern), Mongolia, China, Korea | PAR | Guyer et al. 1977; Döberl 2010 |
| *Phyllotreta iberica*  Heikertinger, 1911 | Unknown | Unknown | Unknown | Spain (North-West) | PAR | Döberl 2010 |
| *Phyllotreta imitatrix*  Heikertinger, 1943 | Unknown | Unknown | Unknown | South Africa | AFR | Heikertinger 1943 |
| *Phyllotreta impressicollis*  Laboissiére, 1942 | Unknown | Unknown | Unknown | Democratic Republic of Congo (Nord Kivu) | AFR | Laboissiére 1942 |
| *Phyllotreta inconspicua*  Chittenden, 1927 | Unknown | Unknown | Unknown | North America | NAR | Chittenden 1927 |
| *Phyllotreta indica*  Chen, 1934 | Unknown | Unknown | Unknown | India (Coromandel) | ORR | Chen 1934 |
| *Phyllotreta inordinata*  Chittenden, 1927 | Unknown | Unknown | Unknown | North America | NAR | Chittenden 1927 |
| *Phyllotreta insularis*  Heikertinger, 1942 | *Crateva formosensis* | Capparaceae | MON | Taiwan | ORR | Lee et al. 2011 |
| *Phyllotreta iridicollis*  Bechyné, 1959 | Unknown | Unknown | Unknown | Democratic Republic of Congo (Nizi) | AFR | Bechyné 1959 |
| *Phyllotreta judaea*  Pic, 1901 | *Reseda lutea, R. alba* | Resedaceae | MON | Armenia, Bulgaria, Slovakia, Iran, Israel, Jordan, Turkey | PAR | Furth 1979 |
| *Phyllotreta koltzei*  Weise, 1887 | Unknown | Unknown | Unknown | Russia (Eastern), Korea | PAR | Döberl 2010 |
| *Phyllotreta konevi*  Lopatin, 1985 | Unknown | Unknown | Unknown | Kazakhstan (Barsa-kelmes Island in Aral Sea) | PAR | Döberl 2010 |
| *Phyllotreta krali*  Lopatin, 1990 | Unknown | Unknown | Unknown | Iran | PAR | Döberl 2010 |
| *Phyllotreta lacerta*  Heikertinger, 1941 | Unknown | Unknown | Unknown | Spain (North-West) | PAR | Döberl 2010 |
| *Phyllotreta laeviceps*  Wollaston, 1887 | *Brassica nigra* | Brassicaceae | MON | Cape Verde | AFR | Heikertinger 1943 |
| *Phyllotreta laticornis*  Chittenden, 1927 | Unknown | Unknown | Unknown | North America | NAR | Chittenden 1927 |
| *Phyllotreta lativittata*  Kutschera, 1860 | Brassicaceae (also reported on *Capparis* spp*.*) | Brassicaceae | OLI | Azerbaijan, Armenia, Greece, Italy, Malta, Cyprus, Iraq, Israel, Jordan, Kazakhstan, Afghanistan, Iran, Lebanon, Oman, Kyrgyzstan, Syria, Russia (Southern), Tajikistan, Turkmenistan, Turkey, Uzbekistan, China (Xizang) | PAR | Baviera and Biondi 2015; Lopatin 2010 |
| *Phyllotreta lepidula*  (LeConte, 1857) | *Sisymbrium altissimum* | Brassicaceae | MON | North America | NAR | Clark et al. 2004 |
| *Phyllotreta lewisii*  (Crotch, 1873) | *Brassica oleracea, B. rapa, Raphanus sativus, Cleome serrulata, C. integrifolia* | Brassicaceae, Cleomaceae | OLI | North America | NAR | Clark et al. 2004 |
| *Phyllotreta libecki*  Schaeffer, 1919 | *Arabis virginica, Brassica napus , B. rapa, Lepidium virginicum, Raphanus sativus, Rorippa obtuse, R. teres* | Brassicaceae | OLI | North America | NAR | Clark et al. 2004 |
| *Phyllotreta lijiangana*  Wang, 1992 | Unknown | Unknown | Unknown | China (Yunnan) | ORR | Döberl 2010 |
| *Phyllotreta lindahli*  Dury, 1906 | Unknown | Unknown | Unknown | North America | NAR | Smith 1979 |
| *Phyllotreta lindemannae*  Scherer, 1963 | Unknown | Unknown | Unknown | Tanzania (Ndanda) | AFR | Scherer 1963 |
| *Phyllotreta lopatini*  Konstantinov, 1992 | Unknown | Unknown | Unknown | Azerbaijan | PAR | Döberl 2010 |
| *Phyllotreta lorestanica*  Warchalowski, 1973 | *Lepidium* spp. | Brassicaceae | MON | Iran, Turkey | PAR | Aslan et al. 2004 |
| *Phyllotreta lubischevi*  Lopatin, 1992 | Unknown | Unknown | Unknown | Kyrgyzstan | PAR | Döberl 2010 |
| *Phyllotreta maculicornis*  Pic, 1906 | *Lepidium draba, Sinapis arvensis* | Brassicaceae | OLI | Syria, Turkey | PAR | Aslan and Alkan 2015; Şen and Gök 2009 |
| *Phyllotreta maculosa*  Lablokoff-Khnzorian, 1968 | Unknown | Unknown | Unknown | Armenia | PAR | Döberl 2010 |
| *Phyllotreta malayana*  Baly, 1877 | Unknown | Unknown | Unknown | Celebes, New Guinea | AUR | Baly 1877 |
| *Phyllotreta mashonana*  Jacoby, 1897 | *Cleome gynandra, Brassica napus* | Cleomaceae, Brassicaceae | OLI | Saudi Arabia, Yemen, Kenya, Ethiopia, South Africa | PAR, AFR | Heikertinger 1943; Maina et al. 2015; Schmutterer 1971 |
| *Phyllotreta melichari*  Heikertinger, 1941 | Unknown | Unknown | Unknown | Spain | PAR | Döberl 2010 |
| *Phyllotreta mexicana* Jacoby, 1885 | Unknown | Unknown | Unknown | Mexico | NTR | Jacoby 1885 |
| *Phyllotreta milneri*  Bryant, 1942 | Unknown | Unknown | Unknown | South Africa | AFR | Bryant 1942 |
| *Phyllotreta misella*  Jakobson 1901 | Unknown | Unknown | Unknown | Russia (Eastern), Mongolia, China | PAR | Döberl 2010 |
| *Phyllotreta mollis*  Konstantinov, 1992 | Unknown | Unknown | Unknown | Uzbekistan | PAR | Döberl 2010 |
| *Phyllotreta mombasensis*  Weise, 1926 | Unknown | Unknown | Unknown | Kenya (Mombasa) | AFR | Weise, 1926 |
| *Phyllotreta mongolica*  Medvedev, 1980 | Unknown | Unknown | Unknown | Mongolia | PAR | Döberl 2010 |
| *Phyllotreta namwalica*  Bryant, 1942 | Unknown | Unknown | Unknown | Zambia | AFR | Bryant 1942 |
| *Phyllotreta natalensis*  Jacoby 1899 | Unknown | Unknown | Unknown | South Africa (KwaZulu-Natal) | AFR | Biondi and D'Alessandro 2012; Heikertinger 1943 |
| *Phyllotreta nemorum*  (Linnaeus, 1758) | *Raphanus raphanistrum, R. sativus, Armoracia rusticana, Barbarea vulgaris, Alliaria petiolata, Aurinia saxatilis , Iberis* spp.*, Sisymbrium* spp.*, Lepidium* spp.*, Erucaria boveana, Sinapis alba, S. arvensis, Capsella bursa-pastoris, Hirschfeldia* spp.*, Dentaria* spp.*, Cardamine* spp.*, Matthiola* spp.*, Arabis* spp.*, Rorippa* spp., Brassica spp. | Brassicaceae | OLI | Europe, Russia (Eastern), Iran, Israel, Kazakhstan, Mongolia, Turkey, Uzbekistan, Tajikistan, Korea | PAR | Baviera and Biondi 2015; Chashchina 2008; Cox 2007; Furth 1979; Gruev and Döberl 2005; Rheinheimer and Hassler 2018 |
| *Phyllotreta nigra*  Laboissiére, 1942 | Unknown | Unknown | Unknown | Democratic Republic of Congo, Rwanda | AFR | Laboissiére 1942 |
| *Phyllotreta nigricornis*  Jacoby 1876 | Unknown | Unknown | Unknown | New Guinea | AUR | Jacoby 1876 |
| *Phyllotreta nigripes impennis*  Peyerimhoff, 1919 | Unknown | Unknown | Unknown | Algeria | PAR | Döberl 2010 |
| *Phyllotreta nigripes nigripes*  (Fabricius, 1775) | *Brassica* spp.*, Eruca vesicaria, Raphanus* spp.*, Sinapis arvensis, S. alba, Hirschfeldia incana, Diplotaxis* spp*., Erysimum* spp*., Barbarea* spp*., Carmelina* spp*., Cardamine* spp., *Alliaria* spp., *Sisymbrium* spp., *Armoraciae rusticana, Matthiola* spp., *Lepidium* spp., *Iberis* spp., *Reseda* spp*., Tropaeolum* spp.*, Cadaba* spp. | Brassicaceae, Resedaceae, Tropaeolaceae, Capparaceae | OLI | Europe, Spain (incl. Canary Islands), Algeria, Egypt, Morocco, Tunisia, Cyprus, Russia (Eastern) Iran, Israel, Kazakhstan, Syria, Tajikistan, Turkey, Kyrgyzstan, Uzbekistan | PAR | Aslan and Alkan 2015; Aslan and Ghahari 2017; Baviera and Biondi 2015; Furth 1979; Şen and Gök 2009; Rheinheimer and Hassler 2018 |
| *Phyllotreta nitidicollis*  Weise, 1888 | Brassicaceae, Resedaceae | Brassicaceae,  Resedaceae | OLI | Italy (Sicily), Algeria, Libya, Morocco, Tunisia | PAR | Baviera and Biondi 2015 |
| *Phyllotreta nitidipennis*  Heikertinger, 1943 | Unknown | Unknown | Unknown | Democratic Republic of Congo, West Africa | AFR | Bechyné 1955 |
| *Phyllotreta nodicornis*  (Marsham, 1802) | *Reseda lutea, R. phyteuma, Sinapis alba, Raphanus raphanistrum, Brassica rapa* | Resedaceae, Brassicaceae | OLI | Europe, Turkey, India | PAR, ORR | Brelih et al. 2003; Cox 2007; Mohr 1966; Rheinheimer and Hassler 2018 |
| *Phyllotreta oblonga*  Chittenden, 1927 | *Armoracia rusticana, Cardamine bulbosa, Lepidium virginicum* | Brassicaceae | OLI | North America | NAR | Clark et al. 2004 |
| *Phyllotreta obtusa*  Chittenden, 1927 | Unknown | Unknown | Unknown | North America | NAR | Chittenden 1927 |
| *Phyllotreta ochripes*  (Curtis, 1837) | *Alliaria petiolata, Rorippa amphibia, Nasturtium officinale, Cardamine amara, C. pentaphyllos, Sinapis arvensis, Brassica napus, Isatis* spp.*, Diplotaxis* spp. | Brassicaceae | OLI | Europe, Russia (Eastern), Iran, Japan (Honshu, Kyushu), Turkey | PAR | Baviera and Biondi 2015; Cox 2007; Doguet 1994; Mohr 1966; Petitpierre 1999; Rheinheimer and Hassler 2018 |
| *Phyllotreta ogloblini*  Shapiro 1960 | Unknown | Unknown | Unknown | Ukraine | PAR | Döberl 2010 |
| *Phyllotreta oltuensis*  Gruev & Asian 1998 | *Capparis ovata* | Capparaceae | MON | Turkey (Erzurum) | PAR | Gruev and Aslan 1998 |
| *Phyllotreta opaca*  Jacoby, 1897 | Unknown | Unknown | Unknown | Zimbabwe | AFR | Bryant 1942 |
| *Phyllotreta oregonenesis*  (Crotch, 1873) | *Brassica oleracea, B. rapa, Lepidium spathulatum, Radicula terrestris, Raphanus sativus, Rorippa palustris, Cleome serrulata, C. integrifolia* | Brassicaceae, Cleomaceae | OLI | North America | NAR | Clark et al. 2004 |
| *Phyllotreta ovalis*  Blatchley, 1921 | Unknown | Unknown | Unknown | North America | NAR | Smith 1979 |
| *Phyllotreta ovata*  Bryant, 1942 | Unknown | Unknown | Unknown | South Africa | AFR | Bryant 1942 |
| *Phyllotreta ozbeki*  Gruev & Asian 1998 | *Lepidium campestre* | Brassicaceae | MON | Turkey (Bayburt), Iran | PAR | Gruev and Aslan 1998 |
| *Phyllotreta pallidipennis*  Reitter, 1891 | *Lepidium* spp*., Capparis* spp. | Brassicaceae, Capparaceae | OLI | Kazakhstan, Russia, Ukraine, Afghanistan, Iran, Pakistan, Kyrgyzstan, Mongolia, Tajikistan, Turkmenistan, Turkey, Uzbekistan, China (Xizang) | PAR | Lopatin 2010 |
| *Phyllotreta panousei*  Hoffmann, 1953 | Unknown | Unknown | Unknown | Mongolia | PAR | Döberl 2010 |
| *Phyllotreta paradoxa*  Lopatin 1956 | *Salsola dendroides* | Amaranthaceae | MON | Afghanistan, Tajikistan | PAR | Lopatin 2010 |
| *Phyllotreta parallela*  (Boieldieu 1859) | *Hirschfeldia incana, Cardaria* spp*.,*  *Isatis* spp*., Diplotaxis* spp*., Alyssum* spp*.* | Brassicaceae | OLI | France, Italy, Portugal, Spain, Algeria, Morocco, Tunisia | PAR | Baviera and Biondi 2015; Doguet 1994; Peyerimhoff 1915 |
| *Phyllotreta parfentjevi*  Shapiro, 1958 | Unknown | Unknown | Unknown | Kazakhstan, Turkmenistan, Uzbekistan, Iran | PAR | Döberl 2010 |
| *Phyllotreta perspicua*  Chittenden, 1927 | Unknown | Unknown | Unknown | North America | NAR | Chittenden 1927 |
| *Phyllotreta peyerimhoffi*  Heikertinger, 1941 | *Reseda* spp*., Caylusea hexagyna, Ochradenus baccatus* | Resedaceae | OLI | Arab Emirates, Israel, Jordan, Saudi Arabia, Egypt (Sinai) | PAR | Furth 1979 |
| *Phyllotreta polita*  Chittenden, 1927 | Unknown | Unknown | Unknown | North America | NAR | Chittenden 1927 |
| *Phyllotreta pontoaegeica*  Gruev 1982 | *Sinapis alba* | Brassicaceae | MON | Bulgaria, Greece, Turkey | PAR | Şen and Gök 2009 |
| *Phyllotreta prasina*  Chittenden, 1927 | *Cardaria* spp. | Brassicaceae | MON | North America | NAR | Clark et al. 2004 |
| *Phyllotreta praticola*  Weise, 1887 | *Capsella bursa-pastoris* | Brassicaceae | MON | Azerbaijan, Russia, Afghanistan, Iran, Kyrgyzstan, Kazakhstan, Mongolia, Tajikistan, Turkey, Uzbekistan, India, China (North-western) | PAR, ORR | Lopatin 2010 |
| *Phyllotreta procera*  (L. Redtenbacher, 1849) | *Reseda* spp*., Caylusea hexagyna, Ochradenus baccatus* (also reported on *Arabis hirsuta, Brassica* spp.*, Capparis spinosa*) | Resedaceae | OLI | Europe, Spain (incl. Canary Islands), Portugal (incl. Madeira), Iran, Israel, Jordan, Tajikistan, Turkey, Turkmenistan, Algeria, Morocco, Tunisia, Tanzania | PAR, AFR | Baviera and Biondi 2015; Biondi 1995; Brelih et al. 2003; Furth 1979; Şen and Gök 2009; Rheinheimer and Hassler 2018 |
| *Phyllotreta pseudoexclamationis*  Konstantinov, 1992 | Unknown | Unknown | Unknown | Georgia | PAR | Döberl 2010 |
| *Phyllotreta punctulata*  (Marsham, 1802) | *Alliaria* spp*., Arabis hirsuta, Armoracia lapathifolia, A. rusticana, Brassica* spp., *Cakile maritima, Diplotaxis muralis, Erysimum verrucosum, Eruca vesicaria, Isatis tinctoria, Lepidium campestre, L. draba, Raphanus sativus, Rapistrum rugosum, Sinapis arvensis, Sisymbrium altissimum, Nasturtium* spp., *Aurinia saxatilis* (also reported on *Reseda* spp.) | Brassicaceae | OLI | Europe, Morocco, Israel, Turkey, North America | PAR, NAR | Baviera and Biondi 2015; Biondi and De Nardis 2001; Brelih et al. 2003; Clark et al. 2004; Cox 2007; Rheinheimer and Hassler 2018 |
| *Phyllotreta pusilla*  Horn, 1889 | *Descurainia pinnata, Arabis alpina, Armoracia rusticana, Brassica* spp.*, Cardaria* spp*., Erysimum* spp*., Iberis* spp*., Lepidium pubicarpum,*  *L. virginicum, Lobularia maritima, Matthiola incana, Raphanus sativus, Nasturtium officinale, Rorippa* spp.*, Sisymbrium* spp., *Cleome* spp.*, Beta vulgaris, Chenopodium quinoa, Spinacia oleracea, Medicago sativa, Phaseolus vulgaris, Pisum sativum, Lactuca sativa, Tropaeolum* spp*., Solanum tuberosum, S. lycopersicum, Bouteloua eriopoda, Hordeum* spp.*, Triticum* spp*., Zea mays, Daucus carota* | Brassicaceae, Capparaceae, Cleomaceae, Fabaceae, Asteraceae, Amaranthaceae, Tropaeolaceae,  Solanaceae, Poaceae,  Apiaceae | POL | North America, Mexico | NAR, NTR | Clark et al. 2004 |
| *Phyllotreta ramosa*  *(*Crotch, 1874) | *Brassica* spp.*, Erysimum asperum, E. cheiri, Matthiola incana, Raphanus sativus, Nasturtium officinale* | Brassicaceae | OLI | North America | NAR | Clark et al. 2004 |
| *Phyllotreta ramosoides*  Smith, 1985 | *Brassica rapa* | Brassicaceae | MON | North America | NAR | Clark et al. 2004 |
| *Phyllotreta randoniae*  Peyerimhoff, 1920 | *Randonia africana, Reseda* spp. | Resedaceae | OLI | Spain, Algeria, Morocco | PAR | Heikertinger 1941 |
| *Phyllotreta rapillyi*  Doguet, 1984 | Unknown | Unknown | Unknown | Algeria | PAR | Döberl 2010 |
| *Phyllotreta rectilineata*  Chen, 1939 | *Cardamine* spp. | Brassicaceae | MON | Russia (Eastern), China, Korea; Oriental Region | PAR, ORR | Yano and Ohsaki 1993 |
| *Phyllotreta reitteri* Heikertinger, 1911 | *Lepidium latifolium* | Brassicaceae | MON | Ukraine (Crimea), Kazakhstan, Turkey, Uzbekistan | PAR | Bieńkowski and Orlova-Bienkowskaja 2018; Hinz et al. 2008 |
| *Phyllotreta reticulata*  Laboissiére, 1942 | Unknown | Unknown | Unknown | Democratic Republic of Congo (Rwindi) | AFR | Laboissiére 1942 |
| *Phyllotreta rhodesiana*  Bryant, 1942 | Unknown | Unknown | Unknown | Zambia | AFR | Bryant 1942 |
| *Phyllotreta robusta*  LeConte, 1878 | *Brassica* spp.*, Lepidium* spp*., Raphanus sativus, Nasturtium officinale, Sinapis arvensis* | Brassicaceae | OLI | North America | NAR | Clark et al. 2004 |
| *Phyllotreta ruficeps*  Weise, 1925 | Unknown | Unknown | Unknown | Sudan, Tanzania | AFR | Heikertinger 1943 |
| *Phyllotreta rufitarsis*  Allard, l859 | *Reseda* spp.*, Moricandia* spp. | Resedaceae, Brassicaceae | OLI | Italy, Spain, Algeria, Spain (Canary Islands), Morocco, Tunisia, Israel, Jordan, Egypt (Sinai); Afrotropical Region | PAR, AFR | Biondi 1995; Furth 1979 |
| *Phyllotreta rufothoracica*  Chen | Unknown | Unknown | Unknown | Vietnam (Tonkin) | ORR | Medvedev 2009 |
| *Phyllotreta rugifrons*  Kuster, 1849 | *Nasturtium officinale* | Brassicaceae | MON | Croatia (Istria), France, Italy, Malta, Spain, Algeria, Morocco, Tunisia, Israel, Syria | PAR | Baviera and Biondi 2015; Doguet 1994; Furth 1979 |
| *Phyllotreta sachalinensis*  L. N. Medvedev, 1973 | Unknown | Unknown | Unknown | Russia (Eastern) | PAR | Döberl 2010 |
| *Phyllotreta sanga*  Bechyné, 1959 | Unknown | Unknown | Unknown | Democratic Republic of Congo (Mayumbe) | AFR | Bechyné 1959 |
| *Phyllotreta schereri*  Medvedev, 2009 | *Capparis* sp. | Capparaceae | MON | Vietnam (Son Lang) | ORR | Medvedev 2009 |
| *Phyllotreta scheuchi*  Heikertinger, 1941 | *Sinapis arvensis, Lepidium draba* | Brassicaceae | OLI | Austria, Bulgaria, Czech Republic, Great Britain, Germany, Hungary, Slovakia, Spain, Ukraine, Mongolia | PAR | Böhme 2005; Rheinheimer and Hassler 2018 |
| *Phyllotreta shirahatai*  Madar, 1959 | Unknown | Unknown | Unknown | Japan (Honshu) | PAR | Döberl 2010 |
| *Phyllotreta sisymbrii*  Weise, 1888 | Unknown | Unknown | Unknown | Azerbaijan, Armenia, Georgia, Russia (Southern), Iran, Syria, Turkey | PAR | Döberl 2010 |
| *Phyllotreta spatulata*  Smith, 1985 | Unknown | Unknown | Unknown | North America | NAR | Smith 1985 |
| *Phyllotreta stehliki*  Madar & Madar, 1968 | Unknown | Unknown | Unknown | Afghanistan | PAR | Döberl 2010 |
| *Phyllotreta striolata*  (Illiger, 1803) | *Brassica* spp.*, Raphanus sativus* var*. acanthiformis, Sinapis arvensis, Matthiola* spp., *Roripa silvestris, Nasturtium officinale, Sisymbrium officinale, Matthiola* spp., *Eruca* spp., *Berteroa* spp., *Cardamine amara* | Brassicaceae | OLI | Europe, China, Japan, Mongolia, Kazakhstan, Nepal, India, Taiwan, Korea; Afrotropical Region; Oriental Region; Nearctic Regions | PAR, ORR, AFR, NAR | Brelih et al. 2003; Mohr 1966; Scherer 1969; Rheinheimer and Hassler 2018 |
| *Phyllotreta subatra*  Wollaston, 1867 | *Sinapis nigra* | Brassicaceae | MON | Cape Verde | AFR | Heikertinger 1943; Wollaston 1867 |
| *Phyllotreta subnitida*  Chittenden, 1927 | Unknown | Unknown | Unknown | North America | NAR | Chittenden 1927 |
| *Phyllotreta subrugosa*  Jacoby, 1891 | Unknown | Unknown | Unknown | Mexico | NTR | Furth 2006 |
| *Phyllotreta subtilior*  Csiki, 1940 | Unknown | Unknown | Unknown | India (Himachal Pradesh) | ORR | Döberl 2010 |
| *Phyllotreta talassicola*  Heikertinger, 1944 | *Lepidium* spp*., Capsella bursa-pastoris* | Brassicaceae | OLI | Afghanistan, Kyrgyzstan, Kazakhstan, Tajikistan, Uzbekistan | PAR | Lopatin 2010 |
| *Phyllotreta temperei*  Doguet, 1974 | *Brassica* spp.*, Reseda glauca* | Brassicaceae, Resedaceae | OLI | Andorra, France, Spain | PAR | Doguet 1994; Petitpierre 1999 |
| *Phyllotreta tenuimarginata*  Jacoby, 1899 | *Gossypium* spp*., Acacia mellifera* | Malvaceae, Fabaceae | POL | Arab Emirates, Yemen, Madagascar, Somalia | AFR | Couilloud 1993; Heikertinger 1943 |
| *Phyllotreta tetrastigma*  (Comolli, 1837) | *Cardamine* spp.*, Nasturtium officinale, Rorippa amphibia, R. microphyllum, Raphanus sativus* | Brassicaceae | OLI | Europe, Turkey | PAR | Brelih et al. 2003; Mohr 1966; Petitpierre 1999; Rheinheimer and Hassler 2018 |
| *Phyllotreta toelgi*  Heikertinger, 1941 | Unknown | Unknown | Unknown | Turkey | PAR | Döberl 2010 |
| *Phyllotreta togana*  Heikertinger, 1943 | Unknown | Unknown | Unknown | Togo | AFR | Bechyné 1955; Heikertinger 1943 |
| *Phyllotreta tomboi*  Lopatin, 1967 | Unknown | Unknown | Unknown | Mongolia | PAR | Döberl 2010 |
| *Phyllotreta transversovalis*  Chittenden, 1927 | Unknown | Unknown | Unknown | North America | NAR | Chittenden 1927 |
| *Phyllotreta tricolor*  Chapuis, 1879 | Unknown | Unknown | Unknown | Ethiopia | AFR | Heikertinger 1943 |
| *Phyllotreta tunisea*  Pic, 1909 | Unknown | Unknown | Unknown | Algeria, Morocco, Tunisia | PAR | Döberl 2010 |
| *Phyllotreta turneri*  Bryant, 1935 | Unknown | Unknown | Unknown | South-West Africa | AFR | Heikertinger 1943 |
| *Phyllotreta ubsunurica*  Medvedev, 1980 | Unknown | Unknown | Unknown | Mongolia | PAR | Döberl 2010 |
| *Phyllotreta ulkei*  Horn, 1889 | Unknown | Unknown | Unknown | North America | NAR | Horn 1889 |
| *Phyllotreta undulata*  (Kutschera, 1860) | *Armoracia rusticana, Raphanus sativus, Brassica* spp.*, Lepidium didymus, Cardamine* spp., *Rorippa* spp., *Nasturtium* spp., *Isatis* spp., *Alliaria* spp. (also reported on *Reseda luteola* and *Tropaeolium majus*) | Brassicaceae | OLI | Europe, Algeria, Russia (Eastern), Kazakhstan, Kyrgyzstan, Mongolia, Turkey, Turkmenistan, Uzbekistan; Australian Region; Nearctic Regions | PAR, AUR, NAR | Baviera and Biondi 2015; Chashchina 2008; Petitpierre 1999; Reid et al. 2012; Samuelson 1973; Rheinheimer and Hassler 2018 |
| *Phyllotreta unicostata*  Jacoby, 1900 | Unknown | Unknown | Unknown | South Africa | AFR | Heikertinger 1943 |
| *Phyllotreta ustulata iraqensis*  Gruev, 1998 | Unknown | Unknown | Unknown | Iraq | PAR | Döberl 2010 |
| *Phyllotreta ustulata ustulata*  Lopatin, 1961 | *Anabasis* spp*., Salsola* spp*.* | Amaranthaceae | OLI | Kazakhstan, Uzbekistan, Tajikistan | PAR | Lopatin 2010 |
| *Phyllotreta utana*  Chittenden, 1920 | *Sisymbrium officinale, Beta vulgaris, Medicago sativa* | Brassicaceae, Amaranthaceae, Fabaceae | POL | North America | NAR | Clark et al. 2004 |
| *Phyllotreta utanula*  Smith, 1985 | Unknown | Unknown | Unknown | North America | NAR | Smith 1985 |
| *Phyllotreta variipennis aegyptiaca*  Pic, 1915 | Unknown | Unknown | Unknown | Algeria, Spain (incl. Canary Islands), Lebanon, Egypt, Saudi Arabia | PAR | Döberl 2010 |
| *Phyllotreta variipennis variipennis* (Boieldieu, 1859) | *Brassica* spp., *Lepidium draba, Diplotaxis tenufolia, Alyssum* spp*., Sinapis arvensis, Hirschfeldia incana, Schouwia purpurea, Rapistrum* spp*., Diplotaxis muralis, Arabis turrita, Sisymbrium* spp*., Capparis spinosa, Reseda* spp. | Brassicaceae, Capparaceae, Resedaceae | OLI | Europe (incl. Canary Islands), Turkey, Morocco, Tunisia, Cyprus, Iran, Iraq, Israel, Turkey; Afrotropical Region | PAR, AFR | Aslan and Alkan 2015; Baviera and Biondi 2015; Biondi 1995; Brelih et al. 2003; Doguet 1994; Furth 1979; Heikertinger 1943 |
| *Phyllotreta vietnamica*  Medvedev, 2009 | Unknown | Unknown | Unknown | Vietnam (Hoa Binh) | ORR | Medvedev 2009 |
| *Phyllotreta vilis*  Weise, 1888 | *Erysimum* spp*.* | Brassicaceae | MON | Bulgaria, Greece, Italy, Croatia, Turkey | PAR | Rozner and Rozner 2008 |
| *Phyllotreta viridicoerulea*  Scherer | Unknown | Unknown | Unknown | Laos, Thailand, Myanmar | ORR | Medvedev, 2009 |
| *Phyllotreta viridicyanea*  Chittenden, 1927 | *Ambrosia acanthicarpa* | Asteraceae | MON | North America | NAR | Clark et al. 2004 |
| *Phyllotreta vittatoides*  Laboissiére, 1942 | Unknown | Unknown | Unknown | Democratic Republic of Congo | AFR | Laboissiére 1942 |
| *Phyllotreta vittula*  (Redtenbacher, 1849) | *Erucastrum gallicum, Neslia paniculata, Agropyron repens, A. caninum, Lepidium* spp*., Zea mais, Beta vulgaris, Hordeum vulgare, Avena* spp*., Fescuta* spp*., Secale* spp*., Sinapis alba, S. arvensis, Raphanus raphanistrum, Fagopyrum esculentum, Erysimum* spp*., Elytrigia repens* | Brassicaceae, Amaranthaceae, Poaceae | POL | Europe, Afghanistan, China, Iran, Kyrgyzstan, Kazakhstan, Mongolia, Tajikistan, Turkey, Korea; Nearctic Region | PAR, NAR | Aslan and Alkan 2015; Brelih et al. 2003; Cox 2007; Vig 1996; Zheng et al., 2004; Rheinheimer and Hassler 2018 |
| *Phyllotreta weiseana*  Jacobson, 1901 | *Lepidium* spp*., Erysimum* spp*. , Sisymbrium* spp*., Nasturtium* spp*., Crambe* spp*., Sinapis* spp*., Thlaspi* spp*., Berteroa* spp*., Brassica* spp*.* | Brassicaceae | OLI | Azerbaijan, Ukraine, Russia (Southern), Iran, Kazakhstan, Turkey | PAR | Lopatin 2010; Sergeev 2008 |
| *Phyllotreta weisei*  Jacoby, 1906 | Unknown | Unknown | Unknown | South Africa | AFR | Heikertinger 1943 |
| *Phyllotreta yoffei*  Furth, 1979 | *Erucaria boveana, Diplotaxis acris, D. harra, Moricandia nitens, Matthiola arabica, Malcomia africana, Reseda stenostachya* | Brassicaceae, Resedaceae | OLI | Israel | PAR | Furth 1979 |
| *Phyllotreta yunnanica*  Chen, 1933 | Unknown | Unknown | Unknown | China (Gansu, Yunnan, Sichuan) | ORR | Döberl 2010 |
| *Phyllotreta zerchei*  Doberl, 1998 | Unknown | Unknown | Unknown | Greece (Peloponnese) | PAR | Döberl 2010 |
| *Phyllotreta ziegleri*  Lohse, 1980 | *Arabis alpina, Cardamine amara* | Brassicaceae | OLI | Austria (Eastern Alps) | PAR | Böhme 2005; Kapp 1994; Kofler 1990 |
| *Phyllotreta zimmermanni*  (Crotch, 1873) | *Arabis ludoviciana, Armoracia lapathifolia,*  *A. rusticana, Barbarea verna, B. vulgaris, Brassica* spp.*, Capsella bursa-pastoris, Dentaria laciniata, Cardamine spp., Lepidium* spp.*, Nasturtium* spp*., Raphanus sativus, Rorippa islandica, R. palustris* | Brassicaceae | OLI | Finland, Sweden, Russia (Eastern); Nearctic Region | PAR, NAR | Clark et al. 2004 |

^1^ All recorded food plants were included (abbreviated spp. when more than two species in the same genus were reported), with refugial or uncommon food plants in parenthesis.

^2^ Only the well documented food plant families were considered.

^3^ Only species with well documented information regarding their food plants had their diet breadth specified.

^4^ Abbreviations of locations: end., endemic; incl., including.

^5^ The references are in regards to the food plants information. For the species where food plant information is unknown, either the catalogue by Döberl (2010) was used as the secondary source of information or the original paper where the species was described.

**REFERENCES**

Aslan EG, Alkan K (2015) The Alticini (Coleoptera: Chrysomelidae: Galerucinae) fauna of Davraz Mountain (Isparta): Comments on host plant and altitude preferences with two new records for Turkish fauna. Turkish Journal of Zoology 39: 488-493. doi:10.3906/zoo-1404-15

Aslan EG, Ghahari H (2017) An annotated synopsis of the flea beetles of Iran with new records (Coleoptera: Chrysomelidae: Galerucinae: Alticini). Transactions of the American Entomological Society 143: 633-667

Aslan I, Özbek H, Warchalowski A (2004) Five new records, new localities and new host plants for the Turkish flea-beetle fauna (Coleoptera: Chrysomelidae: Alticinae). Entomologica Fennica 15: 138-141

Baly JS (1877) Descriptions of new genera and of uncharacterized species of Halticinae. Transactions of the Entomological Society of London: 157-184, 283-323

Baviera C, Biondi M (2015) The Alticini (Coleoptera: Chrysomelidae, Galerucinae) of Sicily: Recent records and updated checklist. Atti della Accademia Peloritana dei Pericolanti, Classe di Scienze Fisiche, Matematiche e Naturali 93: A2-A50. doi:10.1478/AAPP.932A2

Bechyné J (1955) Über die Westafrikanischen Alticiden (Col. Phytophaga). Entomologische Arbeiten aus dem Museum G Frey Band 6: 486-568

Bechyné J (1959) Observation sur les Alticides recueillis au Congo Belge par M.A. Collart (Coleoptera, Phytophaga). Bulletin Institut Royal des Sciences naturelles de Belgique 35: 1-36

Bieńkowski AO, Orlova-Bienkowskaja MJ (2018) Alien leaf beetles (Coleoptera, Chrysomelidae) of European Russia and some general tendencies of leaf beetle invasions. PLoS ONE 13: 1-23. doi:10.1371/journal.pone.0203561

Biondi M (1991) Contributo alla conoscenza dei Crisomelidi Alticini della Macaronesia con descrizione di una nuova specie delle Canarie (Coleoptera, Chrysomelidae, Alticinae). Vieraea 20: 33-38

Biondi M (1992) Note sui Crisomelidi Alticini della fauna di Turchia, con descrizione di tre nuove specie (Coleoptera, Chrysomelidae, Alticinae). Fragmenta Entomologica Roma, 23: 341-354

Biondi M (1994) *Phyllotreta gloriae* a new species from Southern Spain (Coleoptera, Chrysomelidae, Alticinae). Eos 69: 111-114

Biondi M (1995) Gli Alticini Delle Isole Canarie. Fragmenta Entomologica 26: 1-133

Biondi M (1999) The black *Longitarsus* species associated with Boraginaceae in South Africa (Coleoptera, Chrysomelidae, Alticinae). In: Cox ML (Ed). Backhuys Publishers, Leiden, The Netherlands, 515-531

Biondi M, D'Alessandro P (2012) Afrotropical flea beetle genera: A key to their identification, updated catalogue and biogeographical analysis (Coleoptera, Chrysomelidae, Galerucinae, Alticini). ZooKeys 158: 1-158. doi:10.3897/zookeys.252.3414

Biondi M, De Nardis G (2001) I Coleoptera Chrysomelidae del Massiccio del Gran Sasso d'Italia: proposta per uno studio ecologico finalizzato al monitoraggio ambientale. In: Cicolani B (Ed) Monitoraggio Biologico del Gran Sasso. Andromeda Editrice, 36-54.

Boheman CH (1858) Coleoptera. Species novas descripsit. Pp 1–112, pls I-II. In: Virgin C (Ed) Kongliga Svenska Fregatten Eugenies Resa omkring jorden under befäl af C A Virgin, ären 1851–1853 Vetenskapliga Iakttagelser pa H M Konung Oscar den Förstes befallning utgifna af K Svenska Vetenskaps Akademien II Zoologi 1 Insecta P. A. Norstedt & Söner, Stockholm, Sweden.

Boheman CH (1859) Coleoptera. Species novas descripsit. Pp 113–218, pls III. In: Virgin C (Ed) Kongliga Svenska Fregatten Eugenies Resa omkring jorden under befäl af C A Virgin, ären 1851–1853 Vetenskapliga Iakttagelser pa H M Konung Oscar den Förstes befallning utgifna af K Svenska Vetenskaps Akademien II Zoologi 1 Insecta P. A. Norstedt & Söner, Stockholm, Sweden.

Böhme J (2005) Die Käfer Mitteleuropas, Band. K: Katalog (Faunistische Übersicht). Elsevier GmbH, Spektrum Akademischer Verlag München, 515 pp.

Brelih S, Döberl M, Drovenik B, Pirnat A (2003) Material for the Coleoptera fauna (Coleoptera) of Slovenia. 1st Contribution: Polyphaga: Chrysomeloidea (= Phytophaga): Chrysomelidae: Alticinae. Scopolia, Journal of the Slovenian Museum of Natural History, Ljubljana 50: 1-279

Bryant GE (1942) On the African species of *Phyllotreta* (Col., Halticinae). Bulletin of Entomological Research 32: 145-152. doi:10.1017/S0007485300005356

Bukejs A (2008) To the knowledge of flea beetles (Coleoptera: Chrysomelidae: Alticinae) in the fauna of Latvia. 2. Genus *Phyllotreta* Chevrolat, 1836. Acta Zoologica Lituanica 18: 198-206. doi:10.2478/v10043-008-0025-9

Chashchina O (2008) An annotated list of flea beetles (Coleoptera, Chrysomelidae, Halticinae) of the Southern Urals. Entomological Review 88: 164-177. doi:10.1134/S0013873808020048

Chen SH (1934) Revision of the Halticinae (Col. Chrysomelidae) of Yunnan and Tonkin. Sinensia 5: 225-416

Chittenden FH (1927) The species of *Phyllotreta* north of Mexico. Entomologica Americana (ns) 8: 1-63

Čížek P (2003) *Phyllotreta fornuseki* sp. n. (Coleoptera: Chrysomelidae) aus Mähren und aus der Slowakei. Klapalekiana 39: 63-65. doi:10.1360/zd-2013-43-6-1064

Clark SM, LeDoux DG, Seeno TN, Riley EG, Gilbert AJ, Sullivan JM (2004) Host plants of leaf beetle species occurring in the United States and Canada (Coleoptera: Orsodacnidae, Megalopodidae, Chrysomelidae exclusive of Bruchinae). Coleopterist Society, Special Publication no. 2, 476 pp.

Couilloud R (1993) Coléoptères déprédateurs du cotonnier en Afrique et à Madagascar. Supplément à Coton et fibres tropicales. CIRAD-CA, Paris, France, 92 pp.

Cox ML (2007) Atlas of the seed and leaf beetles of Britain and Ireland. Pisces Publications, Newbury, Berkshire, 334 pp.

Döberl M (2010) Subfamily Alticinae. In: Löbl I, Smetana A (Eds) Catalogue of Palearctic Coleoptera. Apollo Books, Stenstrup, Denmark, 491-563

Doguet S (1977) Description d'une nouvelle espèce de Phyllotreta d'Algérie (Col. Chrysomelidae). L’entomologiste 33: 185-187

Doguet S (1994) Coléoptère Chrysomelidae. Volume 2. Alticinae. Faune de France, France et Régions Limitrophes 80. Federation Francaise des Societes de Sciences naturelles, Paris, 694 pp.

Doguet S, Dufay C (1994) Alticinae rares ou nouveaux pour la France (Coleoptera Chrysomelidae). Bulletin mensuel de la Société Linnéenne de Lyon 63: 305-308

Fritzlar F (2009) Neue und interessante Blattkäfer-Nachweise aus Thüringen und anderen Bundesländern (Coleoptera, Chrysomelidae), Teil 5. Thüringer Faunistische Abhandlungen XIV: 181-210

Furth DG (1979) Zoogeography and host plant ecology of the Alticinae of Israel, especially *Phyllotreta*; with descriptions of three new species (Coleoptera: Chrysomelidae). Israel Journal of Entomology 28: 1-37

Furth DG (2006) The current status of knowledge of the Alticinae of Mexico (Coleoptera: Chrysomelidae). Bonner zoologische Beiträge 54: 209-237

Gajendra N, Prasad SK (2016) A Review of Coleoptera Diversity of Chhattisgarh: Updated Checklist 2015. International Journal of Science and Research 5: 710-714

Ghahari H (2017) New Records of Flea Beetles ( Coleoptera: Chrysomelidae: Galerucinae: Alticini) from Iran. Acta Zoologica Bulgarica 69: 501-506

Gruev B, Aslan I (1998) Four new species of the subfamily Alticinae (Coleoptera, Chrysomelidae) from Turkey. Türkiye Entomoloji Dergisi 22: 163-169

Gruev B, Döberl M (2005) General distribution of the flea beetles in the Palaearctic Subregion (Coleoptera, Chrysomelidae: Alticinae) Supplement. Pensoft Publishers, Sofia, Moscow, 241-241 pp.

Guyer GE, Adkisson PL, Chiang CH, Fox GW, Huffaker CB, Maxwell FG, Metcalf RL, Reynolds HT, Roelofs WL, Schwartz B, Weidhaas DE, Beemer HL (1977) Insect Control in the People's Republic of China. A trip report of the American Insect Control Delegation. CSCPRC Report No. 2. National Academy of Sciences, Washington, DC, 218 pp.

Heikertinger F (1941) Bestimmungstabellen europäischer Käfer. (7. Stück.) LXXXII. Fam. Chrysomelidae. 5. Subfam. Halticinae. 1. Gatt. *Phyllotreta* Steph. Bestimmungstabelle der paläarktischen *Phyllotreta*-Arten. Koleopterologische Rundschau 27: 15-64

Heikertinger F (1943) Die *Phyllotreta*-Arten des äthiopischen Faunengebietes. Arbeiten über morphologische und taxonomische Entomologie aus Berlin-Dahlem 10: 33-56

Hinz HL, Gerber E, Cristofaro M, Tronci C, Seier M, Korotyaev BA, Gültekin L, Williams L, Schwarzlaender M (2008) All against one: first results of a newly formed foreign exploration consortium for the biological control of perennial pepperweed. In: Julien MH, Sforza R, Bon MC, Evans HC, Hatcher PE, Hinz HL, Rector BG (Eds) Proceedings of the XII International Symposium on Biological Control of Weeds, La Grande Motte, France, 22-27 April 2007. C.A.B. International, Wallingford, Oxfordshire, UK 162-167

Horn GH (1889) A Synopsis of the Halticini of Boreal America. Transactions of the American Entomological Society and Proceedings of the Entomological Section of the Academy of Natural Sciences 16: 163-320

Iablokoff-Khnzorian SM (1978) Three new species of leaf-beetles from Middle Asia (Coleoptera, Chrysomelidae). Doklady Academii Nauk Armyanskoy SSR. 66: 119-123. [in Russian].

Jacoby M (1876) Harvard Entomology MCZ type Database. Specimen label "Nuova Guinea Fly River, L. M. D'Albertis 1876-77. 1st Jacoby Coll; nigricornis Jac". http://140.247.96.247/mcz/Species_record.php?id=16804 [accessed 02.01.2019]

Jacoby M (1885) Insecta, Coleoptera, Galerucidae. Halticinae. Phytophaga. Biologia Centrali-Americana 6: 263-625

Januš J (2004) Results of a survey of the beetles (Coleoptera) of family Chrysomelidae s. lat. in the territory of the Kĺivoklátsko Protected Landscape Area and Biosphere Reserve. Klapalekiana 40: 55-121 (in Czech)

Kalaichelvan T, Verma KK (2005) Checklist of leaf beetles (Coleoptera: Chrysomelidae) Of Bhilai-Durg. Zoos' Print Journal 20: 1838-1842

Kapp A (1994) *Phyllotreta ziegleri* Lohse, 1980 neu für die Steiermark (Col., Chrysomelidae, Alticinae). Mitteilungen der Abteilung für Zoologie und Botanik am Landesmuseum Joanneum 48: 63-64

Kofler A (1990) Zum Vorkommen von *Phyllotreta zeigkeri* Lohse und *Notaris sethiops* (F.) in Osttriol (Coleoptera: Chrysomelidae, Curculionidae). Koleopterologische Rundschau 60: 131-137

Krupnick GA, Weis AE (1998) Floral herbivore effect on the sex expression of an andromonoecious Plant, *Isomeris arborea* (Capparaceae). Plant Ecology 134: 151-162

Krupnick GA, Weis AE (1999) The effect of floral herbivory on male and female reproductive success in *Isomeris arborea*. Ecology 80: 135–149

Laboissiére V (1942) Halticinae (Coleoptera Phytophaga). Fam. Chrysomelidae. Exploration du Parc National Albert. Mission G.F. De Witte (1933-1935). Institut des Parcs Nationaux du Congo Belge. Fascicule 39, 1-132 pp.

Lee CF, Chang HY, Wang CL, Chen WS (2011) A review of *Phyllotreta* Chevrolat in Taiwan (Coleoptera: Chrysomelidae: Galerucinae: Alticini). Zoological Studies 50: 525-533. doi:10.1053/j.ctsap.2007.02.007

Lopatin IK (2010) Leaf beetles (Insecta, Coleoptera, Chrysomelidae) of Central Asia Minsk: BSU 511 pp. (in Russian)

Maina GD, Harrison W, Linnet G, Chimoita EL (2015) Influence of plant metabolites on flea beetle infestation in spider plant morphotypes. Universal Journal of Plant Science 3: 49-57. doi:10.13189/ujps.2015.030302

Medvedev LN (2009) Alticinae of Indochina. KMK Scientific Press, Moscow, 1-224 pp.

Mohr KH (1966) Chrysomelidae. In: Freude H, Harde KW, Lohse GA (Eds) Die Käfer Mitteleuropas Band 9. Goecke & Evers Verlag, Krefeld, 95-280

Özdikmen H, Coral Şahin D (2017) A new species, *Phyllotreta bilgeae* sp. nov., from Turkey (Chrysomelidae: Galerucinae: Alticini). Munis Entomology & Zoology 12: 175-179

Özdikmen H, Coral Şahin D, Bal N (2017a) *Phyllotreta* Chevrolat in Turkey with a new record (Chrysomelidae: Galerucinae: Alticini). Munis Entomology & Zoology 12: 199-216. doi:10.1097/PEC.0000000000000892

Özdikmen H, Şahin DC, Bal N (2017b) A new species of *Phyllotreta* Chevrolat from Turkey (Chrysomelidae: Galerucinae: Alticini). Munis Entomology & Zoology 12: 147-150

Petitpierre E (1999) Catàleg dels coleòpters crisomèlids de Catalunya IV. Alticinae. Butlletí de la Institució Catalana d'Historia Natural 67: 91-129

Petitpierre E, Bastazo G, Blasco-Zumeta J (2000) Crisomelidos (Coleoptera: Chrysomelidae) de un sabinar de *Juniperus thurifera* L. En Los Monegros (Zaragoza, ne Espana). Boletín Sociedad Entomológica Aragonesa 27: 53-61

Peyerimhoff DP (1915) Notes sur la biologie de quelques Coléoptères phytophages du Nord Africain (2e série). Annales de la Société Entomologique de France 84: 19-61

Ravi GB, Reji Rani OP, Sudharma K, Kavya MK (2014) Insect pests of cabbage and cauliflower and their natural enemies in agro ecosystem of Kerala. Entomon 39: 151-160

Reid C, Booth R, Döberl M (2012) Case 3575 *Haltica undulata* Kutschera, 1860 (currently *Phyllotreta undulata*, Insecta, Coleoptera, chrysomelidae): proposed precedence over *Haltica bivittata* Waterhouse, 1838 (currently *Phyllotreta bivittata*). The Bulletin of Zoological Nomenclature 69: 24-28. doi:10.21805/bzn.v69i1.a11

Rheinheimer J, Hassler M (2018) Die Blattkäfer Baden-Württembergs. Kleinsteuber Books, Karlsruhe, 928 pp.

Rozner I, Rozner G (2008) Data to the leaf-beetle fauna of Macedonia (Coleoptera, Chrysomelidae). Natura Somogyiensis 12: 111-131

Samuelson GA (1973) Alticinae of Oceania (Coleoptera: Chrysomelidae). Pacific Insects 30: 1-165

Scherer G (1963) Beitrag zur Kenntnis der Alticidenfauna Afrikas (Coleoptera, Chrysomelidae, Alticinae). Entomologische Arbeiten 14: 648-684

Scherer G (1969) Die Alticinae des Indischen Subkontinentes (Coleoptera - Chrysomelidae). Pacific Insects Monographs 22: 1-251

Scherer G (1970) Beitrag zur Kenntnis der Alticinae Afrikas (Coleoptera - Chrysomelidae - Alticinae). Entomologische Arbeiten 21: 298-304

Schmutterer H (1971) Contribution to the knowledge of the crop pest fauna in Ethiopia. Zeitschrift für Angewandte Entomologie 67: 371-389. doi:10.1111/j.1439-0418.1971.tb02136.x

Şen I, Gök A (2009) Leaf beetle communities (Coleoptera: Chrysomelidae) of two mixed forest ecosystems dominated by pine – oak – hawthorn in Isparta province, Turkey. Annales Zoologici Fennici 46: 217-232. doi:10.5735/086.046.0306

Sergeev ME (2008) New data to the fauna of Chrysomelidae beetles (Coleoptera) in Lugansk nature reserve. Scientific works of Lugansk nature reserve Vol 1 (Dedicated to the 40-th anniversary of Lugansk nature reserve). Flora and Fauna and their conservation, Lugansk, 131-163

Smith EH (1979) Genus *Tanygaster* Blatchley, a new Synonym of *Phyllotreta* Chevrolat (Coleoptera: Chrysomelidae: Alticinae). The Coleopterists Bulletin 33: 359-362

Smith EH (1985) Revision of the genus *Phyllotreta* Chevrolat of America North of Mexico. Part I. The maculate species (Coleoptera: Chrysomelidae: Alticinae). Fieldiana Zoology, 1-168 pp.

Vela JM, Bastazo G, Fritzlar F (2017) Inventario comentado de los crisomélidos (Coleoptera, Chrysomelidae) de las Sierras Tejeda y Almijara y los Acantilados de Maro (Sur de España , Málaga-Granada). Boletin de la Asociación Española de Entomologia 41: 29-73

Verdyck P (1998) Genetic differentiation and speciation among four *Phyllotreta* species (Coleoptera: Chrysomelidae). Biological Journal of the Linnean Society 64: 463-476. doi:10.1006/bijl.1998.0231

Vig K (1996) Host plant selection by *Phyllotreta vittula* (Redtenbacher, 1849). Proceedings of the Fourth International Symposium on the Chrysomelidae, Proceedings of XX ICE. Museo Regionale di Scienze Naturali, Torino, 233-251

Wollaston TV (1867) Coleoptera Hesperidum: being an enumeration of the Coleopterous insects of the Cape Verde Archipelago. J. Van Voorst, London, 1-336 pp. doi:10.5962/bhl.title.48651

Yadav D, Swaminathan R, Ameta OP (2010) Species diversity and population dynamics of flea beetles on mustard and radish. Indian Journal of Applied Entomology 24: 109-115

Yano S, Ohsaki N (1993) The phenology and intrinsic quality of wild crucifers that determine the community structure of their herbivorous insects. Researches on Population Ecology 35: 151-170. doi:10.1007/BF02513589
